# Novel idebenone derivatives attenuated oxidative stress injury and myocardial damage

**DOI:** 10.3389/fchem.2025.1544616

**Published:** 2025-02-24

**Authors:** Yuwei Peng, Yishan Guo, Xinyi Yang, Yulan Liu, Xun Xu, Junhong Chen, Xueyi Liu, Zhenrou Xie, Zhiqiang Yu, Dudu Wu, Zhi Chen

**Affiliations:** ^1^ Guangdong Provincial Key Laboratory of Research and Development of Natural Drugs, and School of Pharmacy, Guangdong Medical University, Dongguan, China; ^2^ Guangdong Provincial Key Laboratory of New Drug Screening, Southern Medical University, Guangzhou, China

**Keywords:** idebenone derivative, oxidative stress, HO-1 protein, Bax/Bcl-2 protein, cardiovascular disease

## Abstract

Oxidative stress-induced cardiomyocyte apoptosis was the primary causative factor of cardiovascular disease (CVD). However, the existing therapy drugs for oxidative stress were much less investigated, which underlined the necessity for new drug discovery and development. Herein, we aimed to synthesize several novel idebenone (IDE) derivatives and investigate the protective effect and mechanism of these derivatives against H_2_O_2_-induced oxidative stress injury in H9C2 cells by determining cell proliferation rate, detecting the reactive oxygen species (ROS) level, and the expression of related proteins. Additionally, the study also investigated the protective effect of IDE-1 pretreatment on Balb/c mice after hypoxia-reoxygenation. *In vivo* experiments, the damage to cardiomyocytes was assessed using hematoxylin-eosin (HE) staining and terminal deoxynucleotidyl transferase-mediated dUTP-biotin nick end labeling (TUNEL) staining. The results showed that IDE-1 possessed the highest antioxidant damage activity among all IDE derivatives, which could notably decrease the levels of intracellular ROS. Furthermore, the antioxidant mechanism was confirmed to be potentially linked to the expression levels of the oxidation-related pathway heme oxygenase-1 (HO-1) and the apoptosis-related pathway Bcl-2/Bax and caspase-3. Our results demonstrated that IDE derivatives could be a new research direction for the treatment of cardiovascular diseases associated with oxidative stress.

## 1 Introduction

Cardiovascular disease (CVD), which included myocardial infarction, heart failure, and coronary atherosclerosis, had emerged as a serious hazard to human health (Gargi et al., 2024; Gupta et al., 2023). Globally, the prevalence of COVID-19 and the trend of population aging were to blame for the ongoing rise in the morbidity and death rates of CVD (Salabei et al., 2022; Guerricchio et al., 2024). Oxidative stress was one of the primary factors contributing to CVD, including myocardial infarction, heart failure, ischemic heart disease, and cardiomyopathy (van der Pol et al., 2019). Oxidative stress occurred when the body generated an excess of oxidized reactive substances or experiences weakened antioxidant capacity, leading to insufficient scavenging of oxygen metabolites and reactive oxygen species (ROS) (Dubois-Deruy et al., 2020).

Recently, researchers devoted a significant amount of effort to developing preventive and therapeutic strategies against myocardial oxidative stress injury. Most studies have focused on reducing oxidative stress levels using natural compounds or clinical drugs with antioxidant properties, such as Coenzyme Q10, which served as an electron transfer carrier and antioxidant in the mitochondrial oxidative respiratory chain (Arenas-Jal et al., 2020; Hargreaves et al., 2020; Bhagavan and Chopra, 2006). However, the therapeutic efficacy of Coenzyme Q10 was limited by its poor water solubility, low bioavailability, and restricted targeting to mitochondria, which reduced its clinical effectiveness in treating oxidative stress-related cardiovascular diseases (Rabanal-Ruiz et al., 2021).

Idebenone (IDE), as a derivative of coenzyme Q10, contained a redox-active benzoquinone core that enabled it to participate in electron transfer and neutralize ROS (Gueven et al., 2021). Notably, IDE functioned as an antioxidant because it possessed the same redox-active benzoquinone fraction as coenzyme Q10 (Valduga et al., 2022). And IDE inhibited lipid peroxidation and protected cell membranes and mitochondria from oxidative stress damage (Villalaín, 2024; Li et al., 2023). Sonia Shastri et al. reported that IDE significantly reduced the colonic levels of MDA and nitric oxide (NO), while increased the expression of the redox factor NADPH dehydrogenase quinone-1 (NQO-1) in DSS-exposed mice (Shastri et al., 2020). Alvaro Mordente et al. reported that IDE could diphenylpicrylhydrazyl, peroxyl, and tyrosyl radicals, as well as peroxynitrite (Mordente et al., 1998).

**FIGURE 1:**
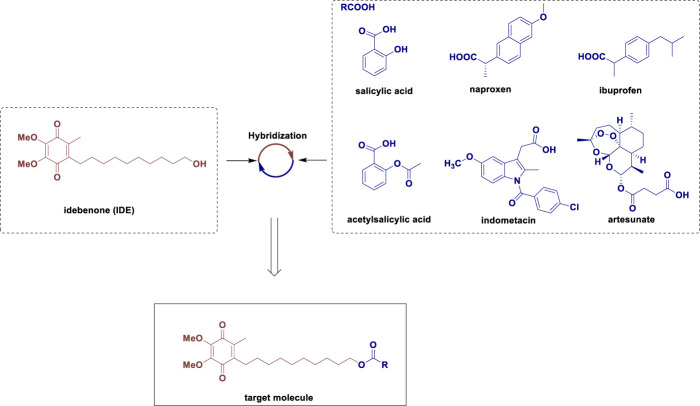
Design of the target molecule.

However, previous literature on IDE mainly focused on the study of the monomer derivatives, and its hybridization with other active structural motifs was much less investigated. Our group was engaged in the structural modification and medicinal studies for small molecule active compounds through the combinatorial chemistry approach, and developed a series of novel small molecule hybrids with excellent bioactivities of antitumor, anti-rheumatoid arthritis and so on (Chen et al., 2020; Tao et al., 2020; Chen et al., 2022; He et al., 2024), which promoted us to continue employing this promising combinatorial chemistry approach for the improvement of novel IDE-based hybrids. As shown in Figure 1, IDE derivatives were designed to combine the antioxidant properties of IDE with the anti-inflammatory activity of reactive organic acids such as salicylic acid, naproxen, ibuprofen, acetylsalicylic acid, indomethacin and artesunate (Shahzad et al., 2019; Shi et al., 2022). We envisaged that the introduction of these active organic acid moieties (RCOOH) to the hydroxy group of IDE through esterification could eventually result in the improved and complementary effects of IDE, as the resulting hybrids could not only possess both structural diversity and inherent biological activity of the parent compounds, but also potentially addressed drug resistance and some of the pre-existing side effects (de Oliveira Pedrosa et al., 2017; Hanikoglu et al., 2020).

In the present study, we prioritized investigating whether the antioxidant properties of IDE derivatives differ from those of the parent compound IDE, specifically in the context of oxidative stress and myocardial injury. Our primary objective was the preliminary validation of the antioxidant activity of the synthesized IDE derivatives (IDE-1, IDE-2, IDE-3, IDE-4, IDE-6, and IDE-7), alongside an initial mechanistic exploration. Specifically, we selected the most promising candidate for further in-depth studies, which included evaluating its protective effects against H2O2-induced oxidative damage in H9C2 cardiomyocytes and assessing its therapeutic potential in a myocardial injury model using Balb/c mice. And the anti-inflammatory activity of then will be systematically investigated in future studies.

## 2 Materials and methods

### 2.1 Chemistry

#### 2.1.1 General

NMR spectra were recorded on (^1^H at 400 MHz (or 500 MHz) and ^13^C at 100 MHz) spectrometers. Chemical shifts (δ) were given in ppm with reference to solvent signals [^1^H NMR: CDCl_3_ (7.26); ^13^C NMR: CDCl_3_ (77.0)]. Mass Spectrometry (MS) was conducted on Sciex (or High-resolution mass spectrometry (HRMS) was conducted on Bruker Apex IV RTMS). Synthetic productswere of >98% purity as analyzed by HPLC analysis (phenomenon luna C18, 5.0 μm, 150 mm*4.6 mm) on Neo-Novi. Thinlayer chromatography (TLC) was carried out on 0.25 mm Haiyang silica gel plates, visualized by exposure to UV light (254 nm). The sample injection was 20 μL. A flow rate of 1.0 mL/min was used with a mobile phase of MeCN in H_2_O. The gradient elution was as follows: 0–5 min, 20% MeCN in H_2_O, v/v; 5–10 min, the volume fraction of MeCN was increased to 40% at a constant rate; 10–15 min, the volume fraction of MeCN was increased to 65% at a constant rate; 15–25 min, the volume fraction of MeCN was increased to 100% at a constant rate; 25–28 min, 100% MeCN; 28–29 min, the volume fraction of MeCN was decreased to 20% at a constant rate; 29–33 min, 20% MeCN in H_2_O, v/v.

#### 2.1.2 10-(4,5-dimethoxy-2-methyl-3,6-dioxocyclohexa-1,4-dien-1-yl)decyl 2-hydroxybenzoate (IDE-1)

To a stirred solution of salicylic acid (41 mg, 0.30 mmol) in MeCN (2 mL) was added IDE (100 mg, 0.30 mmol) and DMAP (4 mg, 0.03 mmol). Then a solution of DCC (91 mg, 0.44 mmol) in MeCN (1 mL) was slowly added to the mixture, which was stirred at room temperature for 15 h. Then water (10 mL) was added to the mixture, which was then extracted with EtOAc (3 × 10 mL). The organic layers were combined, and then washed with brine (10 mL), and dried over Na_2_SO_4_. The solvent was filtered and removed *in vacuo*. The residue was purified by silica gel column chromatography (petroleum ether/EtOAc, 15:1) to afford yellow solid compound IDE-1 (107 mg, 79% yield). R_f_ = 0.5 (petroleum ether/EtOAc, 4:1).

^1^H NMR (400 MHz, CDCl_3_) δ 7.76 (dd, *J* = 8.0, 1.8 Hz, 1H), 7.36 (ddd, *J* = 8.6, 7.1, 1.8 Hz, 1H), 6.88 (dd, *J* = 8.3, 1.1 Hz, 1H), 6.79 (ddd, *J* = 8.1, 7.2, 1.1 Hz, 1H), 4.26 (t, *J* = 6.7 Hz, 2H), 3.91 (d, *J* = 1.9 Hz, 6H), 2.36 (t, *J* = 7.4 Hz, 2H), 1.93 (s, 3H), 1.78–1.62 (m, 2H), 1.37 (dtd, *J* = 9.2, 5.5, 4.3, 1.8 Hz, 2H), 1.32 (d, *J* = 7.4 Hz, 2H), 1.29–1.27 (m, 2H), 1.25 (m, 2H), 1.23 (m, 2H), 1.22 (m, 2H), 1.18 (m, 2H); ^13^C NMR (100 MHz, CDCl_3_) δ 184.35, 183.81, 169.93, 161.39, 144.00, 143.99, 142.71, 138.36, 135.29, 129.59, 118.80, 117.25, 112.33, 65.22, 60.85, 60.83, 29.55, 29.19, 29.15, 29.07, 28.95, 28.44, 28.29, 26.10, 25.69, 11.62. MS (ESI) *m/z* = 481.3 ([M + Na]^+^). HPLC purity (98.32%).

#### 2.1.3 10-(4,5-dimethoxy-2-methyl-3,6-dioxocyclohexa-1,4-dien-1-yl)decyl (S)-2-(6-methoxynaphthalen-2-yl)propanoate (IDE-2)

To a stirred solution of naproxen (68 mg, 0.30 mmol) in MeCN (2 mL) was added IDE (100 mg, 0.30 mmol) and DMAP (4 mg, 0.03 mmol). Then a solution of DCC (91 mg, 0.44 mmol) in MeCN (1 mL) was added to the mixture, which was stirred at room temperature for 2 h. Then water (10 mL) was added to the mixture, which was then extracted with EtOAc (3 × 10 mL). The organic layers were combined, and then washed with brine (10 mL), and dried over Na_2_SO_4_. The solvent was filtered and removed *in vacuo*. The residue was purified by silica gel column chromatography (petroleum ether/EtOAc, 6:1) to afford compound yellow solid IDE-2 (137 mg, 84% yield). R_f_ = 0.75 (petroleum ether/EtOAc, 2:1).

^1^H NMR (400 MHz, CDCl_3_) δ 7.71–7.63 (m, 3H), 7.40 (dd, *J* = 8.4, 1.9 Hz, 1H), 7.14–7.08 (m, 2H), 4.05 (t, *J* = 6.7 Hz, 2H), 3.97 (d, *J* = 1.1 Hz, 6H), 3.89 (s, 3H), 3.83 (q, *J* = 7.0 Hz, 1H), 2.46–2.37 (m, 2H), 1.99 (s, 3H), 1.57 (m, 2H), 1.55 (m, 3H), 1.36 (m, 2H), 1.29 (dd, *J* = 9.1, 6.5 Hz, 2H), 1.25 (m, 2H), 1.21 (m, 2H), 1.19–1.14 (m, 6H); ^13^C NMR (100 MHz, CDCl_3_) δ 184.64, 184.07, 174.68, 157.49, 144.19, 144.17, 142.97, 138.58, 135.76, 133.57, 129.18, 128.83, 126.99, 126.20, 125.82, 118.84, 105.44, 64.79, 61.07, 55.20, 45.43, 29.72, 29.32, 29.27, 29.23, 29.03, 28.64, 28.43, 26.30, 25.67, 18.42, 11.83. MS (ESI) *m/z* = 573.2 ([M + Na]^+^). HPLC purity (98.36%).

#### 2.1.4 10-(4,5-dimethoxy-2-methyl-3,6-dioxocyclohexa-1,4-dien-1-yl)decyl 2-(4-isobutylphenyl)propanoate(IDE-3)

To a stirred solution of ibuprofen (61 mg, 0.30 mmol) in MeCN (2 mL) was added IDE (100 mg, 0.30 mmol) and DMAP (4 mg, 0.03 mmol) Then a solution of DCC (91 mg, 0.44 mmol) in MeCN (1 mL) was added to the mixture, which was stirred at room temperature for 2 h. Then water (10 mL) was added to the mixture, which was then extracted with EtOAc (3 × 10 mL). The organic layers were combined, and then washed with brine (10 mL), and dried over Na_2_SO_4_. The solvent was filtered and removed *in vacuo*. The residue was purified by silica gel column chromatography (petroleum ether/EtOAc, 8:1) to afford yellow liquid compound IDE-3 (134 mg, 86% yield), R_f_ = 0.75 (petroleum ether/EtOAc, 4:1).

^1^H NMR (400 MHz, CDCl_3_) δ 7.19 (d, *J* = 8.1 Hz, 2H), 7.08 (d, *J* = 8.1 Hz, 2H), 4.04 (t, *J* = 6.6 Hz, 2H), 3.98 (d, *J* = 1.1 Hz, 6H), 3.67 (q, *J* = 7.2 Hz, 1H), 2.51–2.41 (m, 4H), 2.00 (s, 3H), 1.89–1.77 (m, 1H), 1.55 (t, *J* = 6.6 Hz, 2H), 1.47 (d, *J* = 7.2 Hz, 3H), 1.39–1.34 (m, 2H), 1.33 (d, *J* = 6.0 Hz, 2H), 1.25 (m, 2H), 1.22 (m, 8H), 0.89 (s, 3H), 0.87 (s, 3H); ^13^C NMR (100 MHz, CDCl_3_) δ 184.69, 184.12, 174.81, 144.22, 143.02, 140.38, 138.62, 137.85, 129.21, 127.10, 64.72, 61.12, 45.14, 44.98, 30.15, 29.79, 29.40, 29.36, 29.30, 29.09, 28.70, 28.46, 26.35, 25.70, 22.34, 18.43, 11.88. MS (ESI) *m/z* = 527.4 ([M + H]^+^). HPLC purity (99.36%).

#### 2.1.5 10-(4,5-dimethoxy-2-methyl-3,6-dioxocyclohexa-1,4-dien-1-yl)decyl 2-acetoxybenzoate(IDE-4)

To a stirred solution of acetylsalicylic acid (53 mg, 0.30 mmol) in MeCN (2 mL) was added IDE (100 mg, 0.30 mmol) and DMAP (4 mg, 0.03 mmol). Then a solution of DCC (91 mg, 0.44 mmol) in MeCN (1 mL) was added to the mixture, which was stirred at room temperature for 2.5 h. Then water (10 mL) was added to the mixture, which was then extracted with EtOAc (3 × 10 mL). The organic layers were combined, and then washed with brine (10 mL), and dried over Na_2_SO_4_. The solvent was filtered and removed *in vacuo*. The residue was purified by silica gel column chromatography (petroleum ether/EtOAc, 8:1) to afford yellow liquid compound IDE-4 (101 mg, 68% yield). R_f_ = 0.5 (petroleum ether/EtOAc, 4:1).

^1^H NMR (400 MHz, CDCl_3_) δ 8.00 (dd, *J* = 7.8, 1.7 Hz, 1H), 7.57–7.49 (m, 1H), 7.29 (td, *J* = 7.6, 1.2 Hz, 1H), 7.08 (dd, *J* = 8.1, 1.2 Hz, 1H), 4.24 (t, *J* = 6.8 Hz, 2H), 3.97 (d, *J* = 1.2 Hz, 6H), 2.42 (t, *J* = 7.4 Hz, 2H), 2.33 (s, 3H), 1.99 (s, 3H), 1.75–1.67 (m, 2H), 1.41–1.37 (m, 2H), 1.34 (d, *J* = 8.6 Hz, 2H), 1.31 (s, 2H), 1.28 (s, 2H), 1.25 (d, *J* = 13.7 Hz, 6H); ^13^C NMR (100 MHz, CDCl_3_) δ 184.64, 184.08, 169.62, 164.47, 150.57, 144.19, 142.98, 138.59, 133.67, 131.66, 125.93, 123.71, 123.40, 65.22, 61.07, 29.73, 29.38, 29.33, 29.25, 29.15, 28.64, 28.54, 26.30, 25.85, 20.97, 11.84. MS (ESI) *m/z* = 523.1 ([M + Na]^+^). HPLC purity (99.13%).

#### 2.1.6 10-(4,5-dimethoxy-2-methyl-3,6-dioxocyclohexa-1,4-dien-1-yl)decyl 2-(1-(4-chlorobenzoyl)-5-methoxy-2-methyl-1H-indol-3-yl)acetate (IDE-6)

To a stirred solution of indometacin (106 mg, 0.30 mmol) in MeCN (2 mL) was added IDE (100 mg, 0.30 mmol) and DMAP (4 mg, 0.03 mmol). Then a solution of DCC (91 mg, 0.44 mmol) in MeCN (1 mL) was added to the mixture, which was stirred at room temperature for 2.5 h. Then water (10 mL) was added to the mixture, which was then extracted with EtOAc (3 × 10 mL). The organic layers were combined, and then washed with brine (10 mL), and dried over Na_2_SO_4_. The solvent was filtered and removed *in vacuo*. The residue was purified by silica gel column chromatography (petroleum ether/EtOAc, 8:1) to afford yellow solid compound IDE-6 (147 mg, 73% yield). R_f_ = 0.5 (petroleum ether/EtOAc, 2:1).

^1^H NMR (400 MHz, CDCl_3_) δ 7.65 (d, *J* = 8.5 Hz, 2H), 7.46 (d, *J* = 8.5 Hz, 2H), 6.96 (d, *J* = 2.5 Hz, 1H), 6.86 (d, *J* = 9.0 Hz, 1H), 6.66 (dd, *J* = 9.0, 2.5 Hz, 1H), 4.08 (t, *J* = 6.7 Hz, 2H), 3.98 (d, *J* = 1.1 Hz, 6H), 3.83 (s, 3H), 3.65 (s, 2H), 2.43 (dd, *J* = 8.5, 6.5 Hz, 2H), 2.38 (s, 3H), 2.00 (s, 3H), 1.65 (m, 2H), 1.62–1.56 (m, 2H), 1.39–1.33 (m, 2H), 1.29 (m, 2H), 1.27 (m, 2H), 1.25 (m, 2H), 1.23 (m, 2H), 1.22 (m, 2H); ^13^C NMR (100 MHz, CDCl_3_) δ 184.69, 184.12, 170.93, 168.25, 156.01, 144.30, 144.26, 143.03, 139.20, 138.64, 135.83, 133.92, 131.14, 130.78, 130.65, 129.08, 114.90, 112.73, 111.62, 101.29, 65.14, 61.10, 55.65, 30.40, 29.77, 29.42, 29.36, 29.29, 29.14, 28.68, 28.56, 26.34, 25.82, 13.31, 11.86. MS (ESI) *m/z* = 700.1 ([M + Na]^+^). HPLC purity (99.41%).

#### 2.1.7 10-(4,5-dimethoxy-2-methyl-3,6-dioxocyclohexa-1,4-dien-1-yl)decyl ((3R,5aS,6R,8aS,9R,10S,12R,12aR)-3,6,9-trimethyldecahydro-12H-3,12-epoxy [1,2] dioxepino [4,3-i]isochromen-10-yl) succinate (IDE-7)

To a stirred solution of artesunate (114 mg, 0.30 mmol) in MeCN (2 mL) was added IDE (100 mg, 0.30 mmol) and DMAP (4 mg, 0.03 mmol). Then a solution of DCC (91 mg, 0.44 mmol) in MeCN (1 mL) was added to the mixture, which was stirred at room temperature for 2 h. Then water (10 mL) was added to the mixture, which was then extracted with DCM (3 × 10 mL). The organic layers were combined, and then washed with brine (10 mL), and dried over Na_2_SO_4_. The solvent was filtered and removed *in vacuo*. The residue was purified by silica gel column chromatography (petroleum ether/EtOAc, 10:1) to afford yellow solid compound IDE-7 (192 mg, 92% yield). R_f_ = 0.52 (petroleum ether/EtOAc, 3:1).

^1^H NMR (500 MHz, CDCl_3_) δ 5.79 (d, *J* = 9.8 Hz, 1H), 5.43 (s, 1H), 4.06 (t, *J* = 6.7 Hz, 2H), 3.98 (s, 6H), 2.75–2.69 (m, 2H), 2.69–2.61 (m, 2H), 2.61–2.52 (m, 2H), 2.44 (t, *J* = 7.5 Hz, 2H), 2.37 (td, *J* = 14.0, 4.0 Hz, 1H), 2.05–2.03 (m, 1H), 2.01 (s, 3H), 1.88 (dq, *J* = 10.1, 3.2 Hz, 1H), 1.80–1.74 (m, 1H), 1.71 (dd, *J* = 13.3, 3.3 Hz, 1H), 1.64–1.61 (m, 1H), 1.59 (m, 1H), 1.52–1.45 (m, 1H), 1.43 (s, 3H), 1.38 (m, 1H), 1.36 (d, *J* = 3.2 Hz, 2H), 1.33 (m, 2H), 1.32 (m, 1H), 1.31 (m, 2H), 1.30 (d, *J* = 3.2 Hz, 2H), 1.29 (m, 2H), 1.28 (m, 2H), 1.27 (m, 2H), 1.25 (m, 2H), 0.96 (d, *J* = 6.0 Hz, 3H), 0.85 (d, *J* = 7.1 Hz, 3H); ^13^C NMR (100 MHz, CDCl_3_) δ 184.33, 183.79, 171.84, 170.83, 143.99, 142.70, 138.33, 104.10, 91.83, 91.18, 79.79, 64.58, 60.84, 53.35, 51.25, 44.93, 36.94, 35.93, 33.81, 31.51, 29.52, 29.39, 29.15, 29.04, 28.92, 28.59, 28.42, 28.26, 26.08, 25.62, 24.31, 21.69, 19.94, 11.75, 11.61. MS (ESI) *m/z* = 727.3 ([M + Na]^+^). HPLC purity (98.14%).

### 2.2 Experimental materials for biological study

H_2_O_2_ (Jiangmen Hengjian Pharmaceutical), H9C2 cell (Cell Culture Center, Shanghai, China), DMEM medium and DMSO (Beijing Solarbio Science and Technology), FBS (Life Technologies), CCK-8 kit (Zeta life), RIPA, BCA protein concentration determination kit and SDS-PAGE gel configuration kit (Shanghai Beyotime Biotechnology), Bcl-2 rabbit polyclonal antibody, Bax rabbit polyclonal antibody, caspase-3 rabbit polyclonal antibody, and HO-1 polyclonal antibody (Proteintech), Signal Up™ Primary Antibody Dilution Buffer for Western Blot and Immunol Fluorescence Staining Secondary Antibody Dilution Buffer (Beyotime).

### 2.3 Cell culture and grouping

PC12 and H9C2 cells were cultured at 37°C and 5% CO₂ using a high glucose medium (DMEM) containing 10% fetal bovine serum (FBS). Cells were inoculated into 96-well plates at a density of 10000 cells/well. The oxidative stress injury model was established by treating H9C2 cells with 500 µM H_2_O_2_. H9C2 was divided into control group, model group (H_2_O_2_), the low concentration group (1 μM IDE derivatives + H_2_O_2_), the medium concentration group (5 μM IDE derivatives + H_2_O_2_), and the high concentration group (10 μM IDE derivatives + H_2_O_2_).

### 2.4 Assay of antioxidant damage activity

H9C2 cells in the logarithmic growth phase were inoculated into 96-well plates at 10000 cells/well and cultured at 37°C with 5% CO_2_ for 24 h. After grouping treatment according to 2.3, 10 μL of CCK-8 reagent was added, and the incubation was continued for 2 h. Optical density (OD) value was measured at 450 nm using an enzyme marker to calculate the cell survival rate.

Cell survival rate = [(As-Ab)/(Ac-Ab)]×100% (As: experimental group; Ac: control group; Ab: blank group).

### 2.5 Cell activity assay

PC12 and H9C2 cells in the logarithmic growth phase were inoculated into 96-well plates at 10000 cells/well and cultured in a 37°C, 5% CO_2_ cell culture incubator for 24 h. After that, the medium was changed and the cells were treated with IDE-1 (1, 10, 50, 100 μM) for 24 h. After treatment, 10 μL of CCK-8 was added to each well, incubated at 37°C for 2 h, and Optical density (OD) value was measured at 450 nm with a microplate reader.

### 2.6 Reactive oxygen species (ROS) assay

H9C2 cells in the logarithmic growth phase were cultured in 6-well plates at 15 0000 cells/well, divided into control group, model group (H_2_O_2_) and IDE-1 group (5 μM IDE-1+ H_2_O_2_), with three parallel wells in each group. After being incubated in 37°C, 5% CO_2_ cell culture incubator for 24 h, the cells were washed twice with PBS, and stained with 10 μM DCFH dissolved in serum-free medium at 37°C, protected from light for 20 min, then washed three times with PBS, and added to serum-free medium. The cells were stained with 10 μM DCFH dissolved in serum-free medium for 20 min at 37°C under light protection.

### 2.7 Detection of oxidation-related indexes SOD, MDA and CAT

H9C2 cells in the logarithmic growth phase were cultured in 6-well plates at 15 0000 cells/well, divided into control group, model group (H_2_O_2_) and IDE-1 group (5 μM IDE-1+ H_2_O_2_), with three parallel wells in each group. After being incubated in 37°C, 5% CO_2_ cell culture incubator for 24 h, the cells were processed using an ultrasonic cell breaker (3 s/time, 10 s interval, 30 times), centrifuged (12000 r/min, 4°C, 10 min), and the levels of MDA, CAT and SOD were detected according to the instructions of the kit.

### 2.8 Detection of Caspase-3, Bcl-2, Bax and HO-1 protein expression

H9C2 cells of each group were inoculated in 10 cm dishes at 1 × 10^7^ cells/well, cultured in 37°C, 5% CO_2_ cell incubator for 24 h. After rinsing with PBS for twice, 98 μL PIPA lysate, 1 μL PMSF and 1 μL protease inhibitor were added to each well. After lysis on ice for 30 min, the cell lysates were collected by scraper and centrifuged (centrifugation radius 5.5 cm, 12,000 r/min, 4°C for 15 min), and the supernatant was collected for protein quantification by the BCA method. Then the protein samples of each group were taken for 10% SDS-polypropylene gel electrophoresis, and the separated proteins were electrotransferred to the PVDF membrane. The membrane was covered in skimmed milk powder for 2 h. The antibody was diluted by adding the primary antibody dilution (1:1000) and incubated overnight at 4°C. After the membrane was washed with PBST, fluorescent secondary antibody (1:2000) was added and incubated for 2 h at room temperature, and developed by a visualizer. The gray value of protein bands was analyzed by ImageJ using β-actin as the internal reference protein reference.

### 2.9 Animal experiments

The experimental animals were 5-week-old female Balb/c mice of Specific Pathogen Free (SPF) grade, with an average body mass of 20 g. The mice were randomly divided into four groups: control group, model [isoproterenol (ISO)] group, IDE-1 group, and treatment group (ISO + IDE-1). The study began by administering 20 mg/kg of IDE-1 to mice in the IDE-1 and treatment groups via gavage, while mice in the control and model groups received an equal amount of distilled water via gavage once a day for 10 days. Subsequently, 20 mg/kg of ISO was subcutaneously injected into the backs of mice in the model groups and treatment for 3 consecutive days starting from day 7, while mice in the control group received an equal amount of saline subcutaneously (de Oliveira Pedrosa et al., 2017). Ten days later, all mice were anesthetized and euthanized. The hearts, livers, spleens, lungs, and kidneys of the control group and IDE-1 group were stained with H&E for *in vivo* toxicity testing. Myocardial damage was examined through H&E and Tunel staining of the hearts of mice in the control, model, and treatment groups.

### 2.10 Statistical analysis

SPSS24.0 statistical software was applied to analyze the data, and the measurement data were expressed by (x ± s), one-way ANOVA was performed between multiple groups, and two-by-two comparisons were made by LSD test, and the difference was regarded as statistically significant at P < 0.05.

## 3 Results

### 3.1 Synthesis of IDE derivatives

**SCHEME 1:**
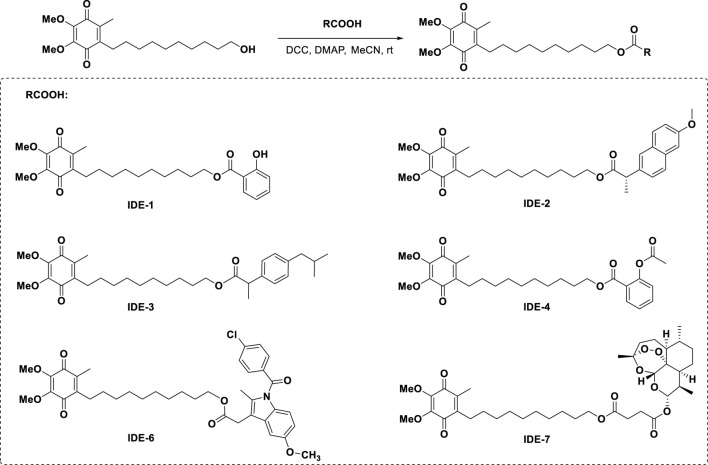
Synthesis of the target molecules.

As shown in Scheme 1, we synthesized a series of IDE-acid hybrids through esterification. Under the condition of DCC and DMAP, IDE underwent esterification with the organic acid (salicylic acid, naproxen, ibuprofen, acetylsalicylic acid, indometacin, and artesunate, respectively) to generate the IDE-acid hybrids IDE-1-IDE-7 in good yields. The structure of the target compounds was confirmed by ^1^H NMR, ^13^CNMR and ESI-MS spectra.

### 3.2 Assay of antioxidant damage activity

**FIGURE 2:**
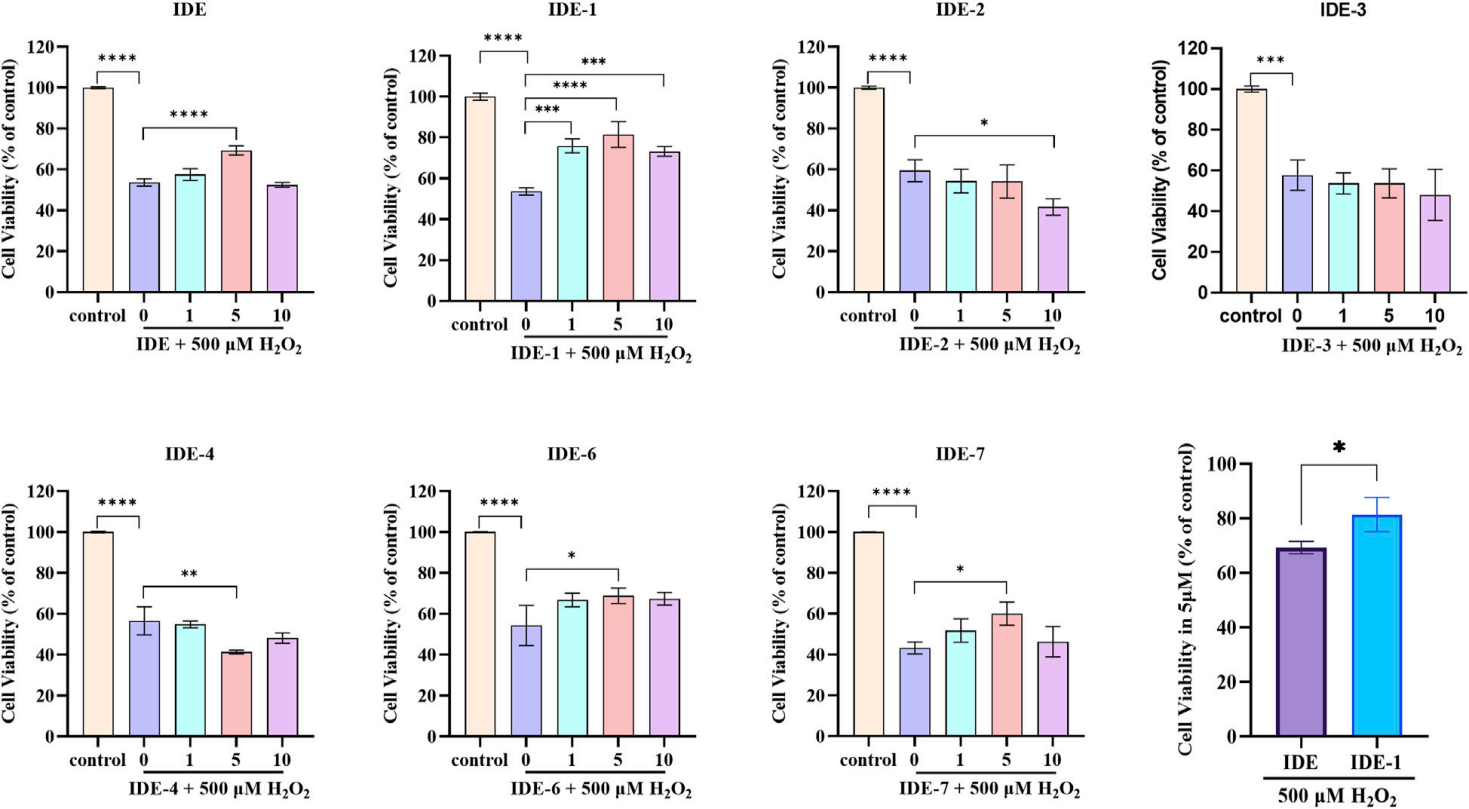
Survival of H9C2 cells after stimulation with idebenone derivatives (1, 5, and 10 µM) for 24 h, CCK-8 assay showed the cell viability of H9C2s n = 3, *P < 0.05,* *P < 0.01, ***P < 0.001 and ****P < 0.0001 vs. Control.

The antioxidative damage activity of IDE derivatives was investigated. As shown in Figure 2, each H_2_O_2_-injured group was significantly different from the control groups with 40%–60% cell survival, indicating that our oxidative damage model was successfully established. The groups IDE-1, IDE-6, and IDE-7 exhibited higher cellular activity following drug administration in comparison to the H_2_O_2_-injured group. These findings suggested that IDE-1, IDE-6, and IDE-7 could effectively prevent oxidative damage in cells. However, the administration of IDE-2, IDE-3, and IDE-4 did not significantly increase the activity of the H9C2 cells, indicating a lack of anti-myocardial oxidative damage activity. Among all the derivatives, the IDE-1 group showed the highest cardiomyocyte activity when administered at 5 μM, indicating that IDE-1 exhibited the best antioxidant activity. Therefore, subsequent mechanistic experiments would focus on IDE-1.

### 3.3 Cytotoxicity assay

**FIGURE 3:**
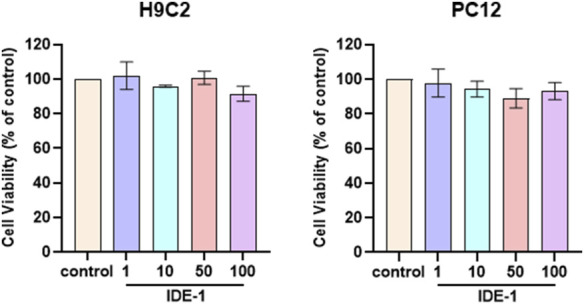
H9C2 and PC12 were stimulated with 1, 10, 50, and 100 µM of IDE-1 for 24 h, CCK-8 assay showing the cell viability of H9C2 and PC12. n = 3, *P < 0.05.

The toxicity experiments of IDE-1 were conducted on H9C2 and PC12 cells. As shown in Figure 3, there was no significant change in the activity of the H9C2 and PC12 cells after the administration of IDE-1. These results indicated that low toxicity of IDE-1.

### 3.4 Reactive oxygen species (ROS) detection

**FIGURE 4:**
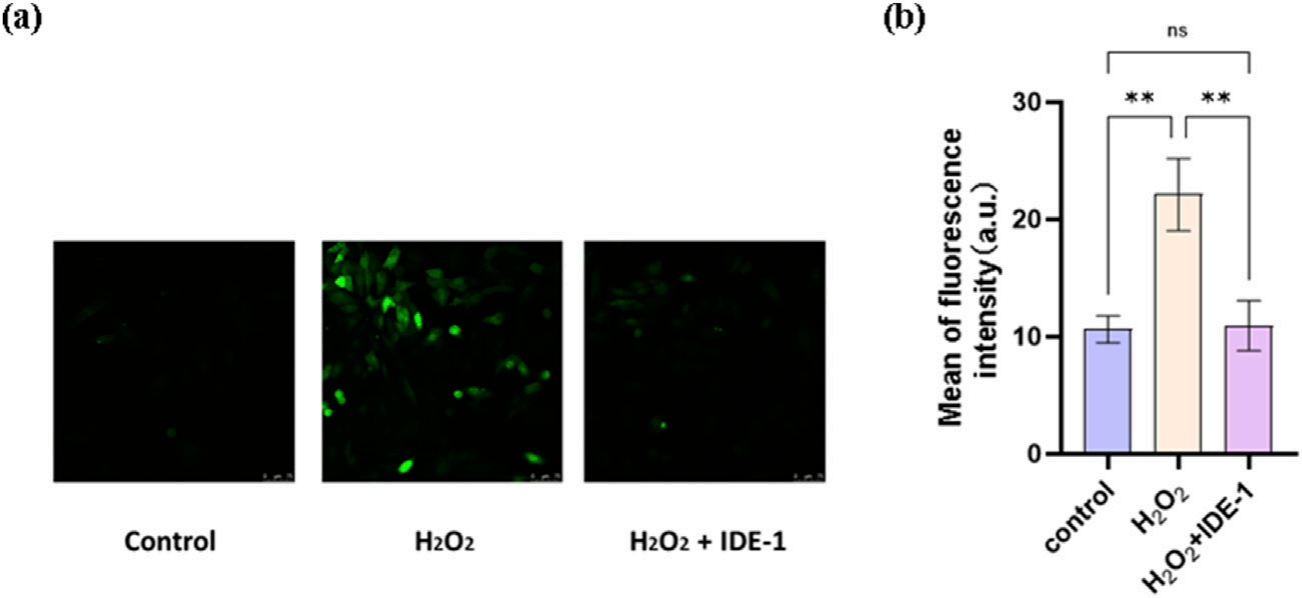
ROS were detected by H2DCFDA staining, **(A)** representative photographs showing IDE-1 on ROS levels in oxidatively damaged H9C2 cells, **(B)** ImageJ analysis of the levels of the groups (control, H_2_O_2_, H_2_O_2_ + IDE-1). n = 3, ^ns^P > 0.05, **P < 0.01.

The ROS level in H9C2 cells was measured using the fluorescent probe H2DCFDA. As shown in Figure 4, the H_2_O_2_ group exhibited a noteworthy rise in fluorescence intensity in comparison to the control group, suggesting an increase in ROS production in H9C2 cells following oxidative damage. However, in the group that received IDE-1 treatment, the fluorescence intensity decreased significantly, indicating that IDE-1 effectively inhibited the generation of ROS. These results suggested that IDE-1 possessed antioxidant properties by reducing ROS generation in cells damaged by oxidation.

### 3.5 Detection of SOD, MDA and CAT levels as oxidation-related indexes

**FIGURE 5:**
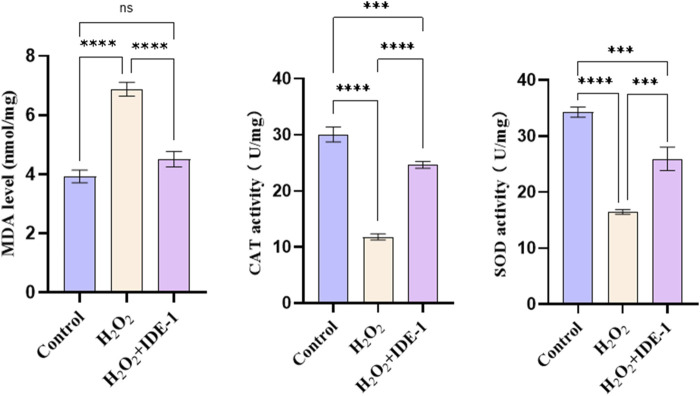
Effect of IDE-1 on the activity of endogenous antioxidant enzymes MDA, CAT and SOD in H9C2 cells. n = 3, ^ns^P > 0.05, ***P < 0.001, ****P < 0.0001.

Figure 5 showed the effect of IDE-1 on MDA levels and CAT, SOD activities in oxidatively damaged H9C2 cells. The treatment of H9C2 cells with IDE-1 resulted in increased activity of endogenous antioxidant enzymes CAT and SOD compared to the H_2_O_2_ group. This result suggested that IDE-1 might protect the cells from ROS-induced damage by enhancing the activities of antioxidant enzymes. Additionally, MDA was a product of lipid peroxidation commonly used to measure levels of lipid peroxidation. In comparison to the H_2_O_2_ group, the level of MDA in the H9C2 cells of the IDE-1 group decreased, indicating that IDE-1 might decrease the lipid peroxidation level of the cells.

### 3.6 Effect of IDE-1 on apoptosis-related and oxidation-related proteins in oxidatively damaged H9C2 cells

**FIGURE 6:**
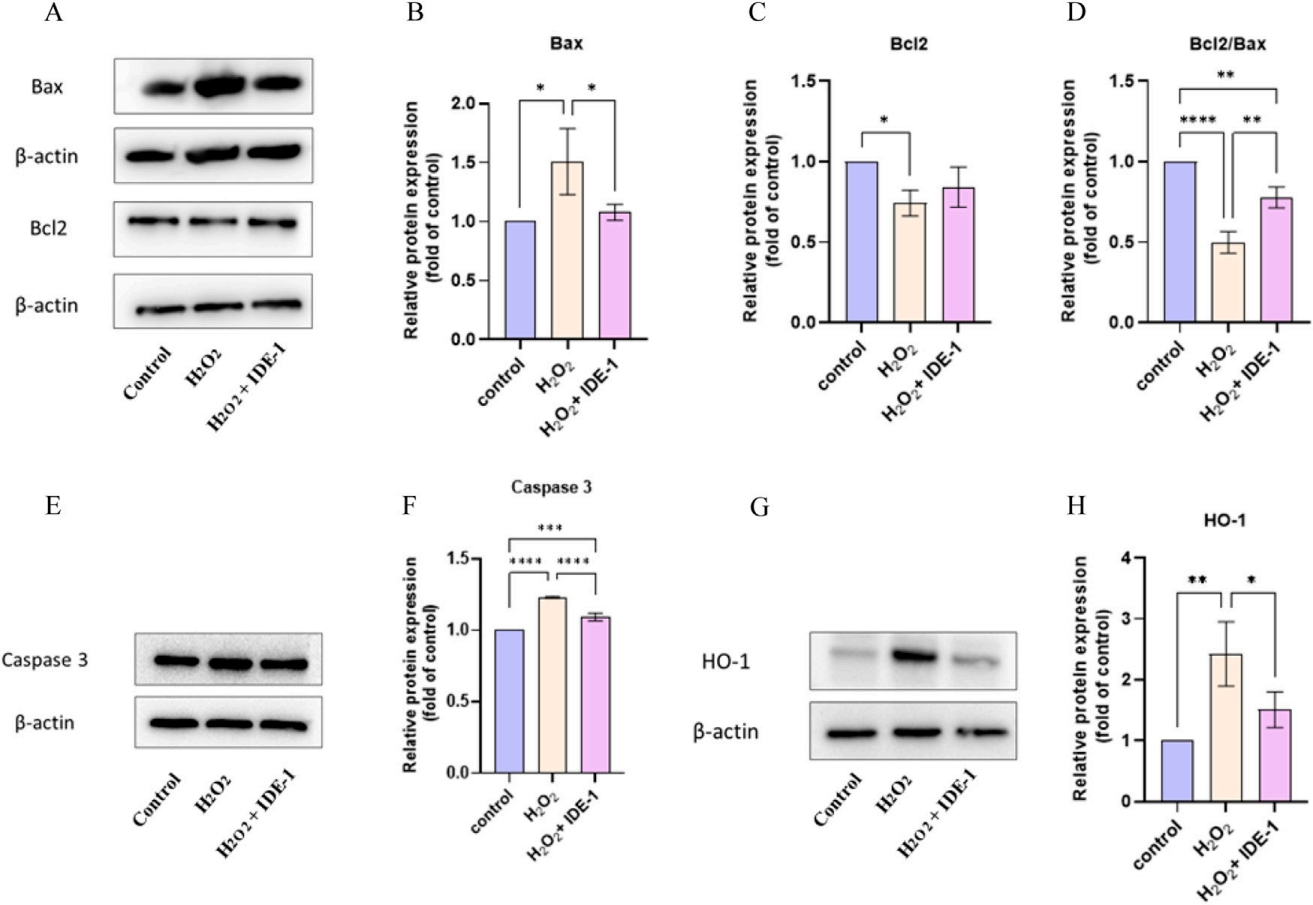
Western blot and quantification analysis showed the protein expression of Bax, Bcl-2, Caspase 3, and HO-1 in IDE-1 treated H9C2 cells. **(A)** Representative Western blot images of Bax and Bcl-2 alongside the loading control β-actin. **(B)** Quantitative analysis of relative Bax protein expression normalized to control. **(C)** Relative Bcl-2 expression analysis showing significant differences across treatment groups. **(D)** The ratio of Bcl2 to Bax, indicating the pro-apoptotic and anti-apoptotic balance. **(E)** Western blot images for Caspase 3 and β-actin as a loading control. **(F)** Quantitative analysis of relative Caspase 3 expression, illustrating significant changes between groups. **(G)** Representative Western blot images for HO-1 with corresponding β-actin control. **(H)** Quantitative analysis of HO-1 protein expression relative to control conditions. *n* = 3, *P < 0.05, **P < 0.01, ***P < 0.001, ****P < 0.0001.

The effect of IDE-1 on apoptosis-related and oxidation-related proteins in oxidatively damaged H9C2 cells was investigated. Figures 6A–D displays the expression of Bax and Bcl-2 proteins in H9C2 cells. Bax protein expression was significantly increased, and Bcl-2 protein expression was significantly downregulated in the H_2_O_2_ group compared to the control group. In the IDE-1 administration group, Bax protein expression was significantly downregulated compared to the H_2_O_2_ group. It was worth noting that a significant decrease in Bcl-2/Bax values after oxidative damage was observed, but the decrease was significantly inhibited by IDE-1 administration. Figures 6E, F shows the expression of Caspase-3 protein in H9C2 cells. It was significantly increased in the model group compared to the control group after oxidative damage. However, in the IDE-1 group, the expression of Caspase-3 protein was significantly downregulated compared with the model group. These results suggested that the mechanism by which IDE-1 inhibited oxidative damage might be related to its interference with the expression of Bcl-2/Bax and Caspase-3 proteins, which inhibited cardiomyocyte apoptosis.

Figures 6G, H shows the expression of the HO-1 protein. It was seen that the relative expression level of HO-1 protein was significantly upregulated in H9C2 cells with H_2_O_2_ damage, and the relative expression level of HO-1 protein was reduced in the IDE-1 group compared with the H_2_O_2_ group. This result indicated that mechanism of IDE-1 against oxidative stress might be linked to the regulation of the expression level of the oxidation-related protein HO-1.

### 3.7 Pathological changes of myocardial tissue detected

**FIGURE 7:**
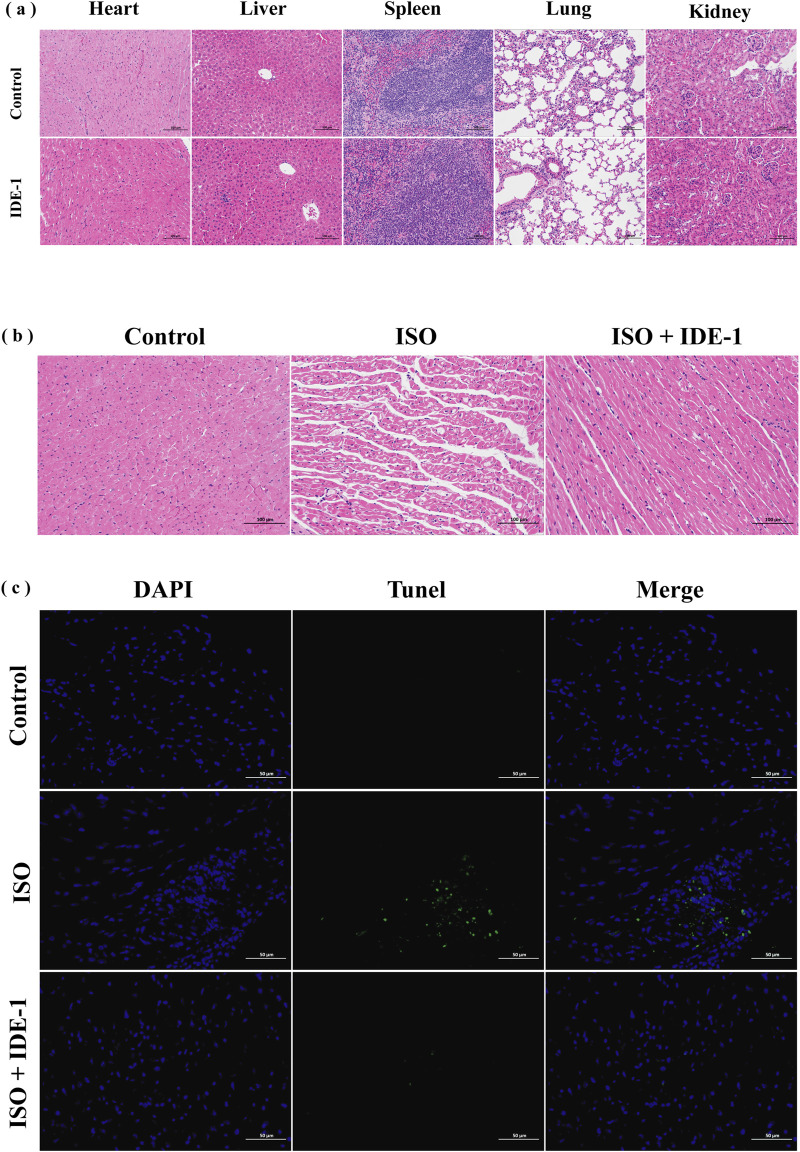
*In vivo* systemic toxicity study and therapy of IDE-1 (20 mg/kg), n = 6. **(A)** Histological analysis of main organs after treatment with IDE-1 (200×). **(B)** Histological analysis of IDE-1 treated myocardial tissue (200×). **(C)** Tunel immunofluorescence staining of IDE-1 treated myocardial tissue (400×). (scale bar: 100 μm).

The toxicity and therapeutic effects of IDE-1 *in vivo* were investigated. Figure 7A shows HE-stained sections of the major organs of mice. The pathological section analysis of the major organs (heart, liver, spleen, lung and kidney) of the mice showed no significant changes, suggesting that IDE-1 has favorable preliminary biosafety. Figure 7B shows HE-stained sections of cardiac tissue after myocardial injury in mice treated with IDE-1. The control group showed clear morphology and structure of myocardial tissue cells, with dense cell arrangement, abundant cytoplasm, and clear nuclei. In contrast, the ISO group showed myocardial fiber breakage, blurred myocardial transverse stripe, interstitial edema, erythrocyte exudation, and inflammatory infiltration, indicating the success of the myocardial injury model. After administration of IDE-1, the arrangement and structure of myocardial cells were still disorganized, with slight hemorrhage, and there were still some broken myocardial fibers, but the damage to myocardial tissue cells was significantly improved. Figure 7C shows the results of Tunel staining to detect apoptosis in myocardial tissue. The ISO group exhibited significant green fluorescence and increased apoptosis compared to the control group. However, after IDE-1 administration, there was a significant decrease in green fluorescence compared to the ISO group. These findings suggested that IDE-1 might be effective in combating myocardial injury, primarily through its anti-apoptotic effects.

## 4 Discussion

Cardiomyocytes were highly susceptible to oxidative stress, which was closely related to the development of cardiovascular diseases (Saadaoui et al., 2022). Hydrogen peroxide (H_2_O_2_) was a common ROS in cardiomyocytes. When oxidative stress occurred in cardiomyocytes, the excessive accumulation of H_2_O_2_ produced toxic effects on cells, damaged cellular DNA and proteins, and induced cell lesions, necrosis, and apoptosis. Consequently, H_2_O_2_ was a commonly used inducer in models of myocardial oxidative stress. In this paper, we established a model of oxidative stress injury by using H_2_O_2_ as an oxidative inducer to damage commonly used myocardial H9C2 cells (Yu et al., 2024).

In addition, IDE was regarded as one of the most effective antioxidants due to its ability to prevent oxidative damage and improve mitochondrial function (Lin et al., 2015). To discover more effective drugs, we synthesized a series of novel IDE hybrids with high antioxidant activity through chemical splicing. Among these compounds, IDE-1 was found to be the most effective in increasing the survival of H9C2 cells subjected to oxidative damage, indicating that IDE-1 showed potential as a novel cardioprotectant and its pharmacology was further investigated.

The Bcl-2 family regulated apoptosis and was primarily located in the mitochondrial and nuclear membranes, as well as the rough endoplasmic reticulum (Hussain et al., 2022). It played a significant role in regulating apoptosis in H9C2 cells when they were damaged by underwent pathological changes (Ren et al., 2023). The Bcl-2 family was divided into two categories based on their functions: pro-apoptotic members, such as Bax, and anti-apoptotic members, such as Bcl-2 (Sovilj et al., 2024). The strength of the inhibitory effect of apoptosis was affected by the ratio between the two proteins, Bcl-2 and Bax (Faridvand et al., 2018). The experiments showed that adding IDE-1 to H9C2 cells increased Bcl-2/Bax expression and decreased Caspase-3 expression, which are key regulators of apoptosis. These findings suggest that IDE-1 protected cardiomyocytes from oxidative damage by modulating apoptosis-related pathways. However, to further elucidate the precise mechanisms, additional pathway inhibition studies targeting the Bcl-2/Bax and Caspase-3 pathways, as well as transcriptomic analyses, could be conducted.

HO-1 was a noticeable rate-limiting enzyme in heme catabolism and inhibited oxidative stress and lipid peroxidation by reducing ros production (Cui et al., 2024; Li et al., 2020). Following the administration of IDE-1, ROS production was significantly suppressed, as demonstrated by ROS fluorescence experiments. Thus, the mechanism of action against oxidative stress might be linked to its capacity to decrease the production of ROS and regulate the expression of the oxidation-related protein HO-1.

Finally, an isoproterenol-induced cardiomyocyte injury model was established in Balb/c mice. Female mice were chosen as the animal model for modeling based on the findings of Wexler et al. that there were sex differences in the ISO-induced myocardial injury model, and that females have the advantages of lower mortality and less cardiac fibrosis, making them a good model for assessing antioxidant damage (Wexler et al., 1974). The results of HE staining and Tunel staining confirmed that IDE-1 improved the injury status of myocardial tissue. The study results indicated that cardiomyocyte apoptosis were reduced in the cardiomyocytes of mice after administration of IDE-1, suggesting that it had protective effects on myocardial tissues. Potential mechanisms of IDE-1 protection might include reduction of oxidative stress and cardiomyocyte apoptosis.Admittedly, the current study was primarily based on preliminary histological analysis, and comprehensive evaluations such as serum biomarker assays and long-term toxicity studies were not performed. Additionally, the potential role of IDE-1 in other cardiovascular disease mechanisms such as its anti-inflammatory effects, vascular function improvement, and other related aspects was not explored. To address these limitations, we plan to conduct more systematic toxicity assessments and in-depth mechanistic studies in future research. In summary, IDE derivatives might be a promising and viable option for research against oxidative stress in cardiomyocytes.

## 5 Conclusion

**FIGURE 8:**
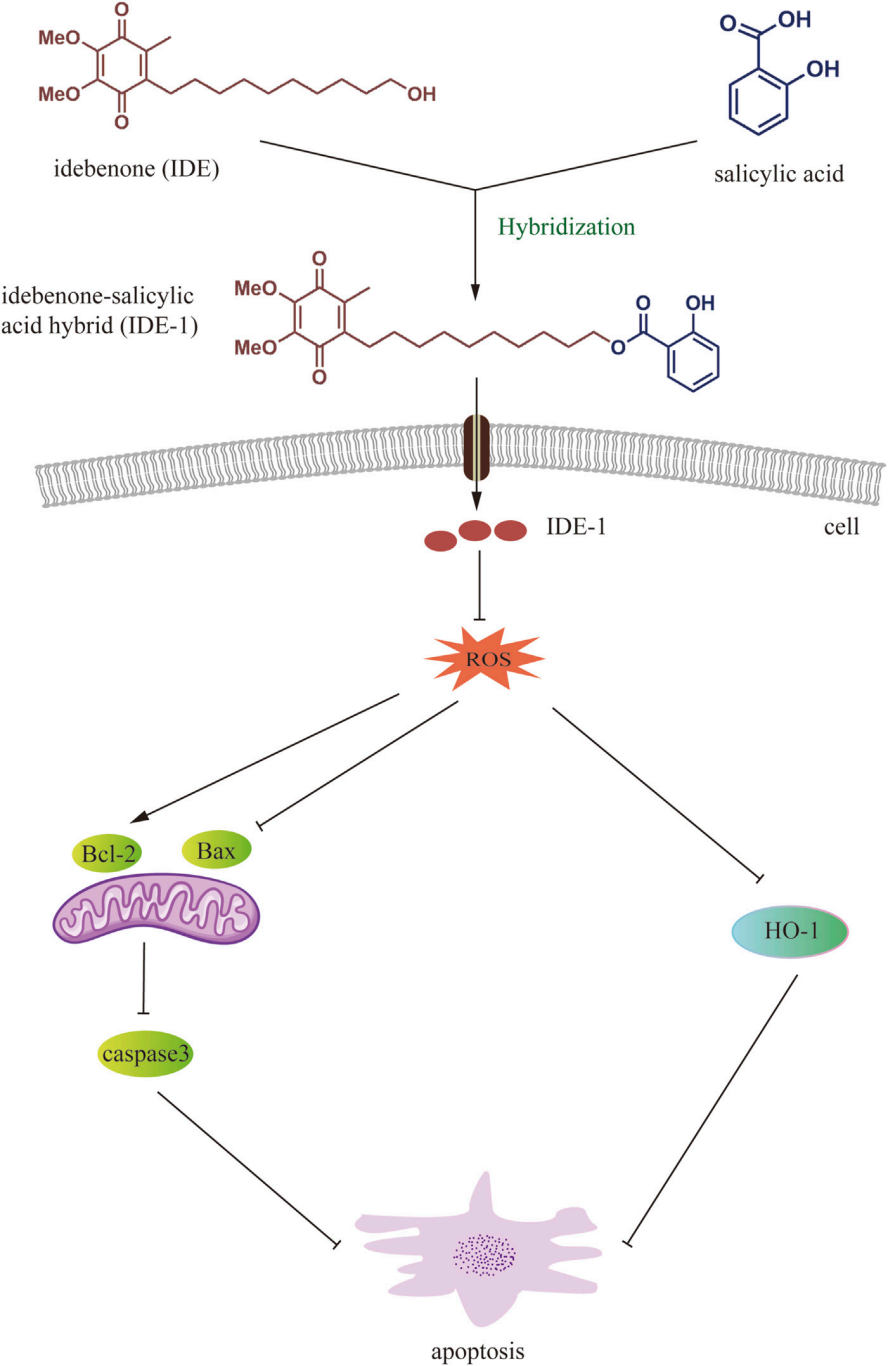
Working model of IDE-1 in exerting antioxidant effects through ROS-mediated anti-apoptotic pathway and oxidation-related HO-1 pathway.

In this study, we successfully synthesized a series of novel IDE hybrids, which significantly enhanced the activity of H9C2 cells subjected to oxidative stress by H_2_O_2_. The results showed that IDE-1 might play an antioxidant role by decreasing the amount of ROS production. This mechanism might also be related to the regulation of the apoptosis-related pathways Bcl-2/Bax and Caspase-3, and the oxidation-related pathway HO-1 (Figure 8). Our findings demonstrated that IDE-1 had potent antimyocardial damage effects *in vivo* without toxicity to major organs. Our results demonstrated that IDE derivatives could be a new research direction for the treatment of cardiovascular diseases associated with oxidative stress and would also and provide valuable insight for expanding the scope of the combinatorial chemistry approach in novel drug discovery associated with oxidative stress.

## Data Availability

The original contributions presented in the study are included in the article/Supplementary Material, further inquiries can be directed to the corresponding author.
