# Supplementary figures and images for: Novel idebenone derivatives attenuated oxidative stress injury and myocardial damage
Source: Front Chem. 2025 Feb 24;13:1544616. doi: 10.3389/fchem.2025.1544616 (PMC11891201; doi:10.3389/fchem.2025.1544616)

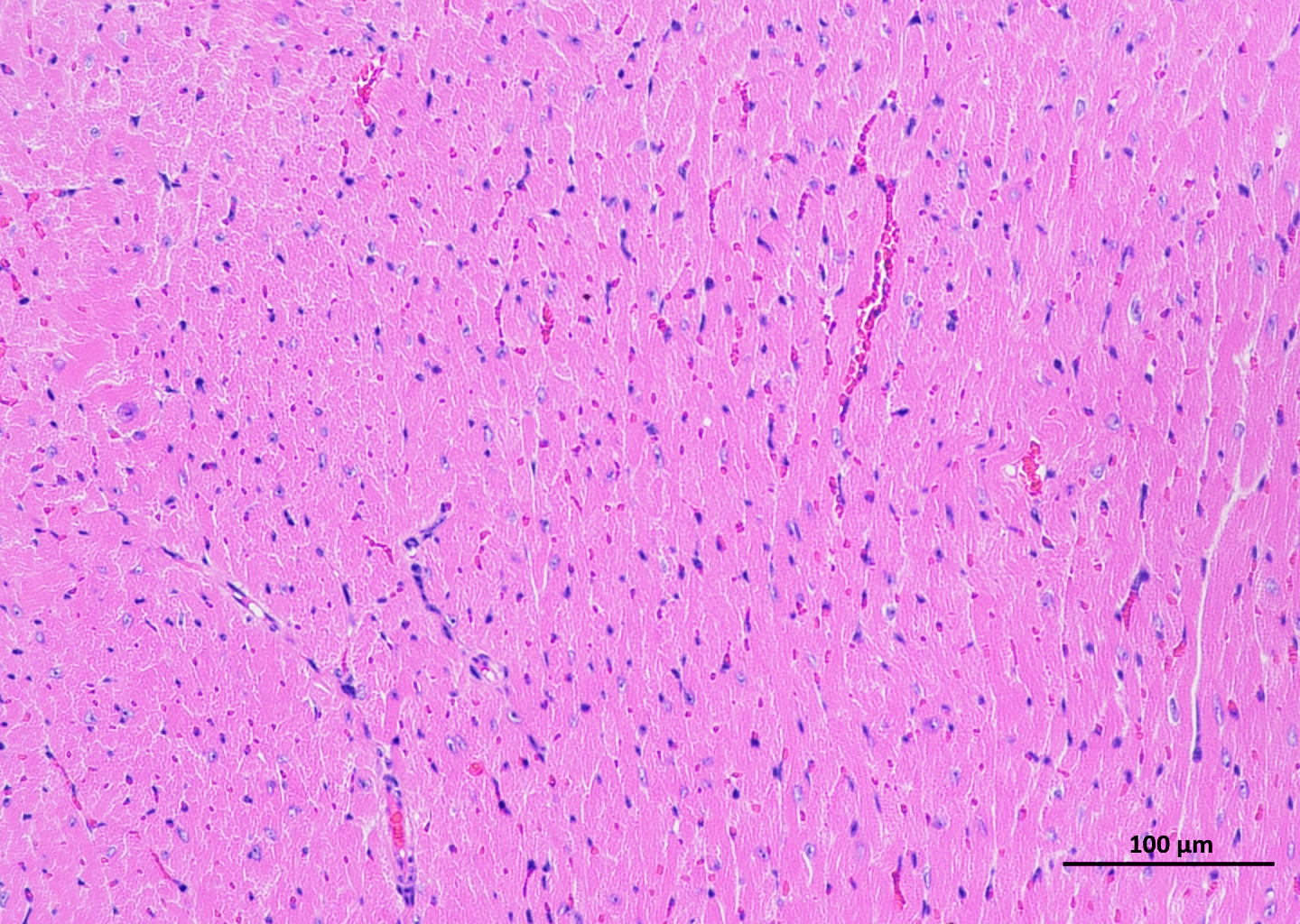

Supplement: Supplementary file 1 [file DataSheet3.zip › HE organ control Original data/control-heart.tif]

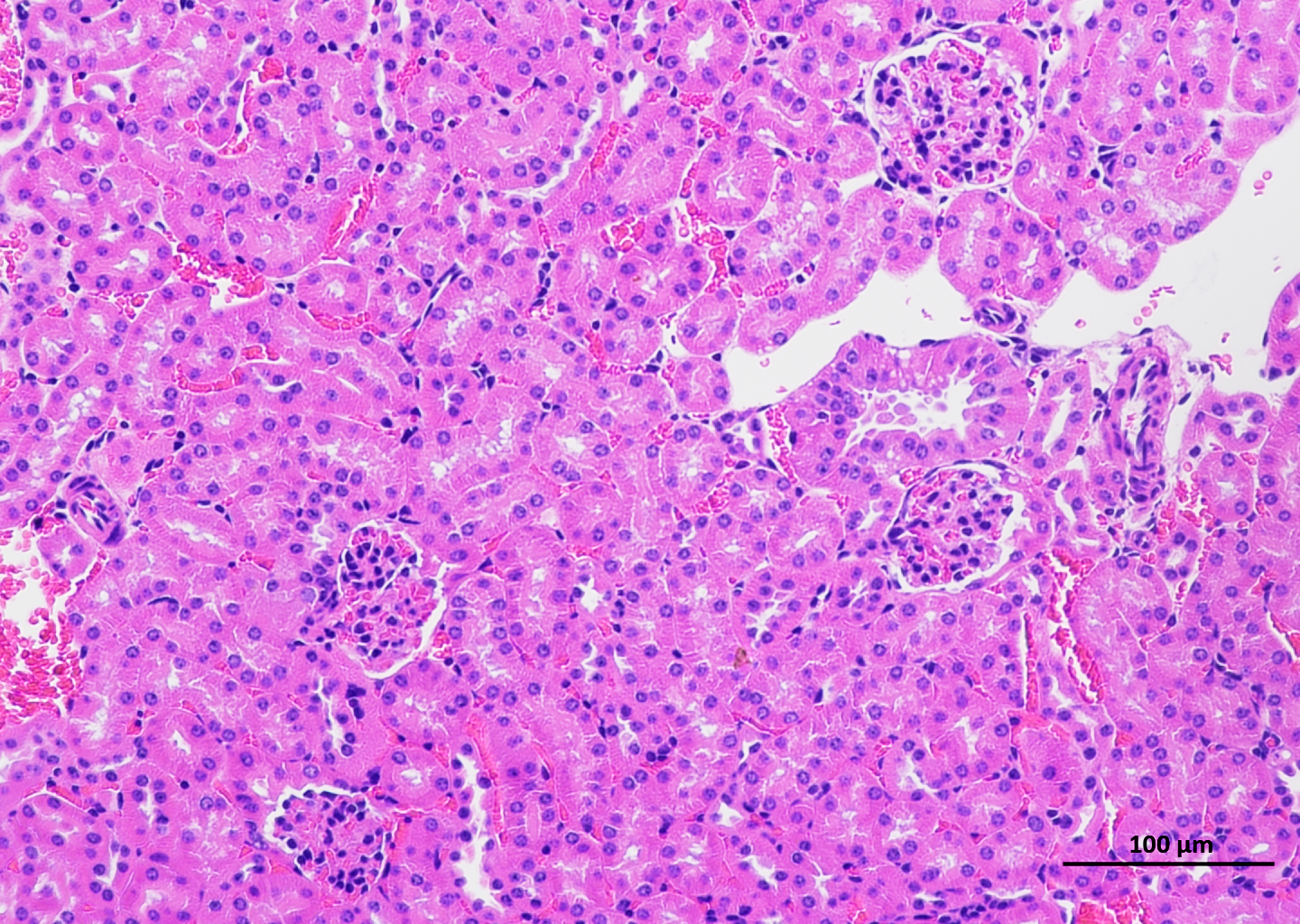

Supplement: Supplementary file 1 [file DataSheet3.zip › HE organ control Original data/control-kidney.tif]

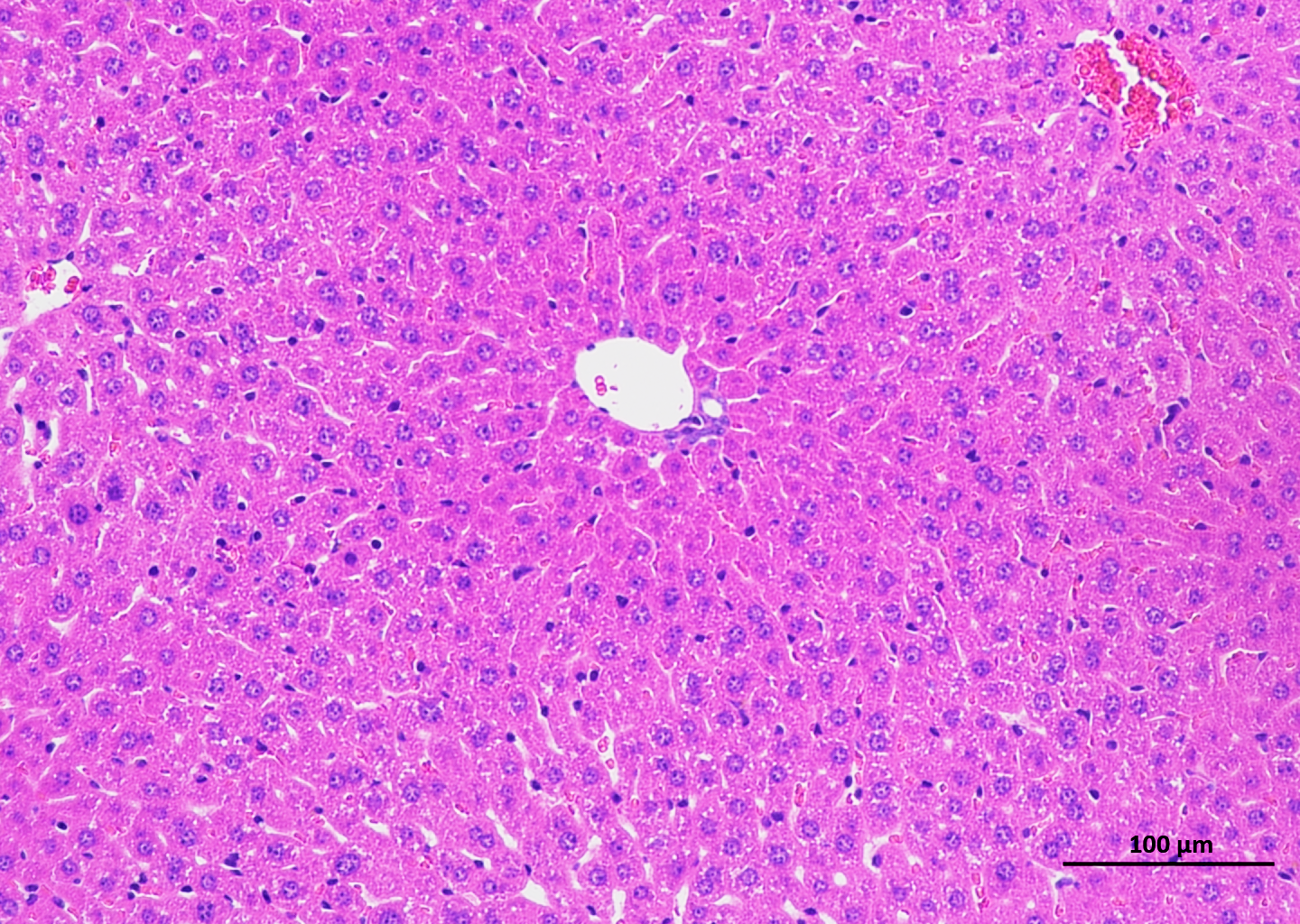

Supplement: Supplementary file 1 [file DataSheet3.zip › HE organ control Original data/control-liver.tif]

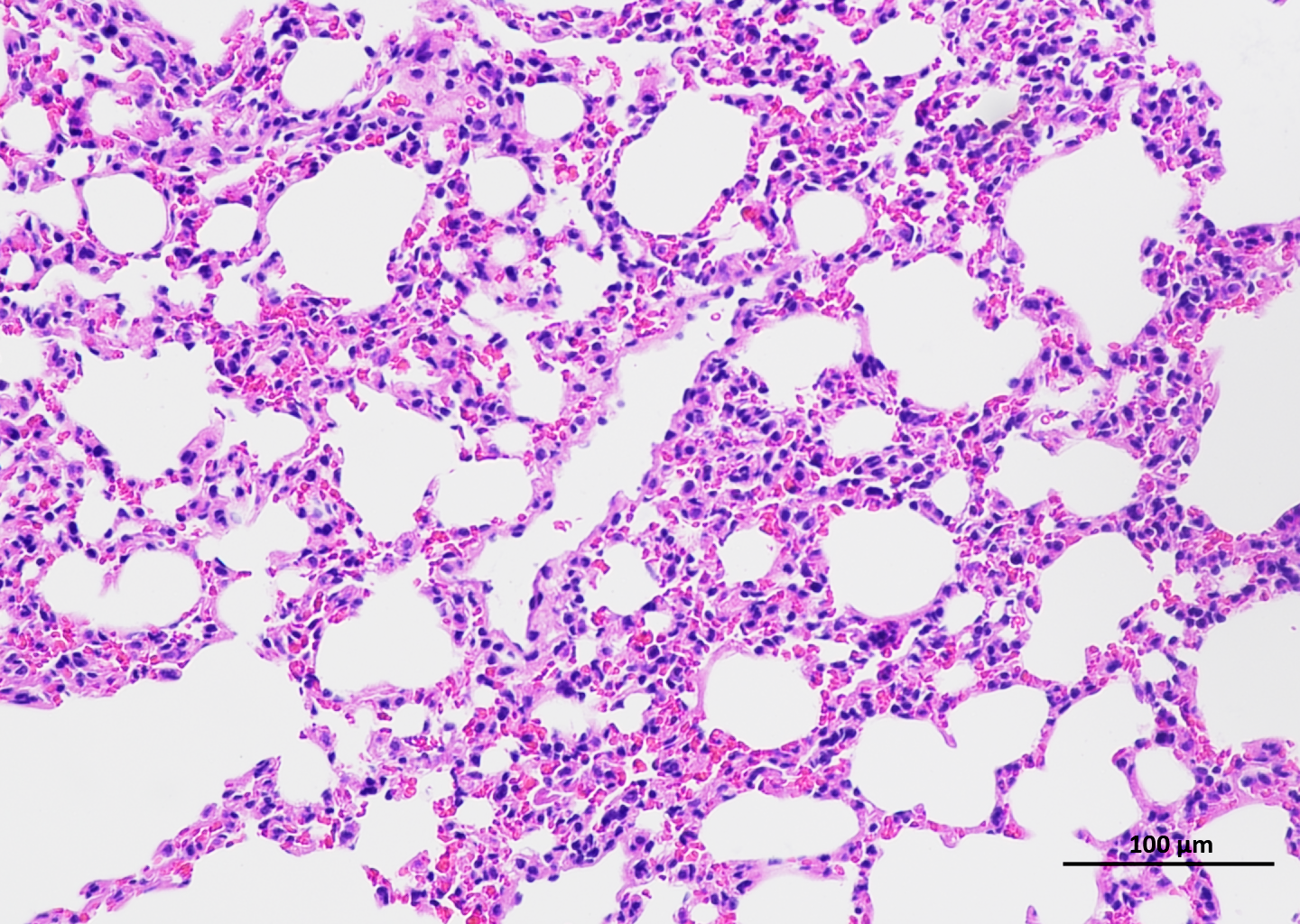

Supplement: Supplementary file 1 [file DataSheet3.zip › HE organ control Original data/control-lung.tif]

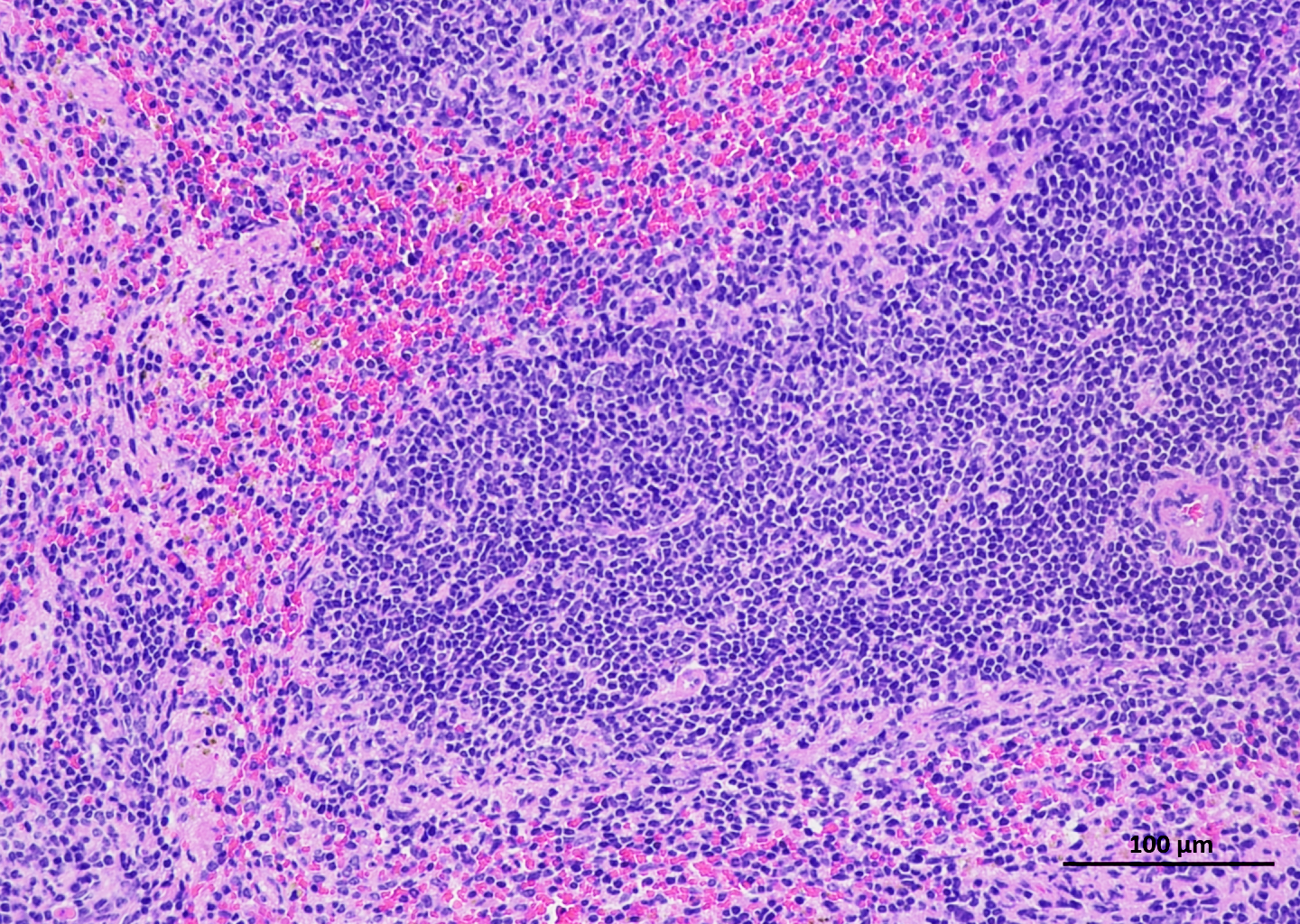

Supplement: Supplementary file 1 [file DataSheet3.zip › HE organ control Original data/control-spleen.tif]

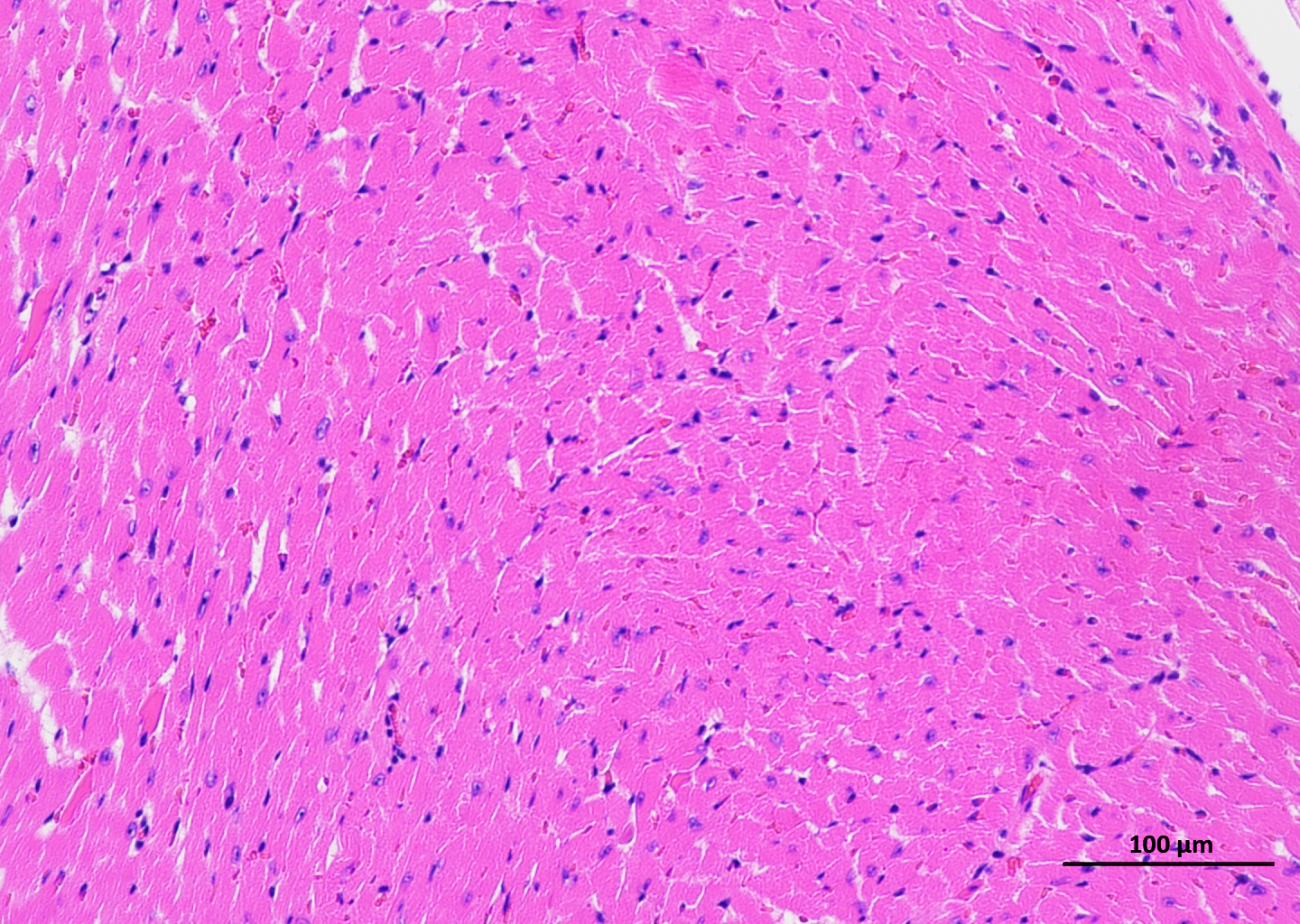

Supplement: Supplementary file 3 [file DataSheet4.zip › HE organ IDE-1 Original data/IDE-1-heart.tif]

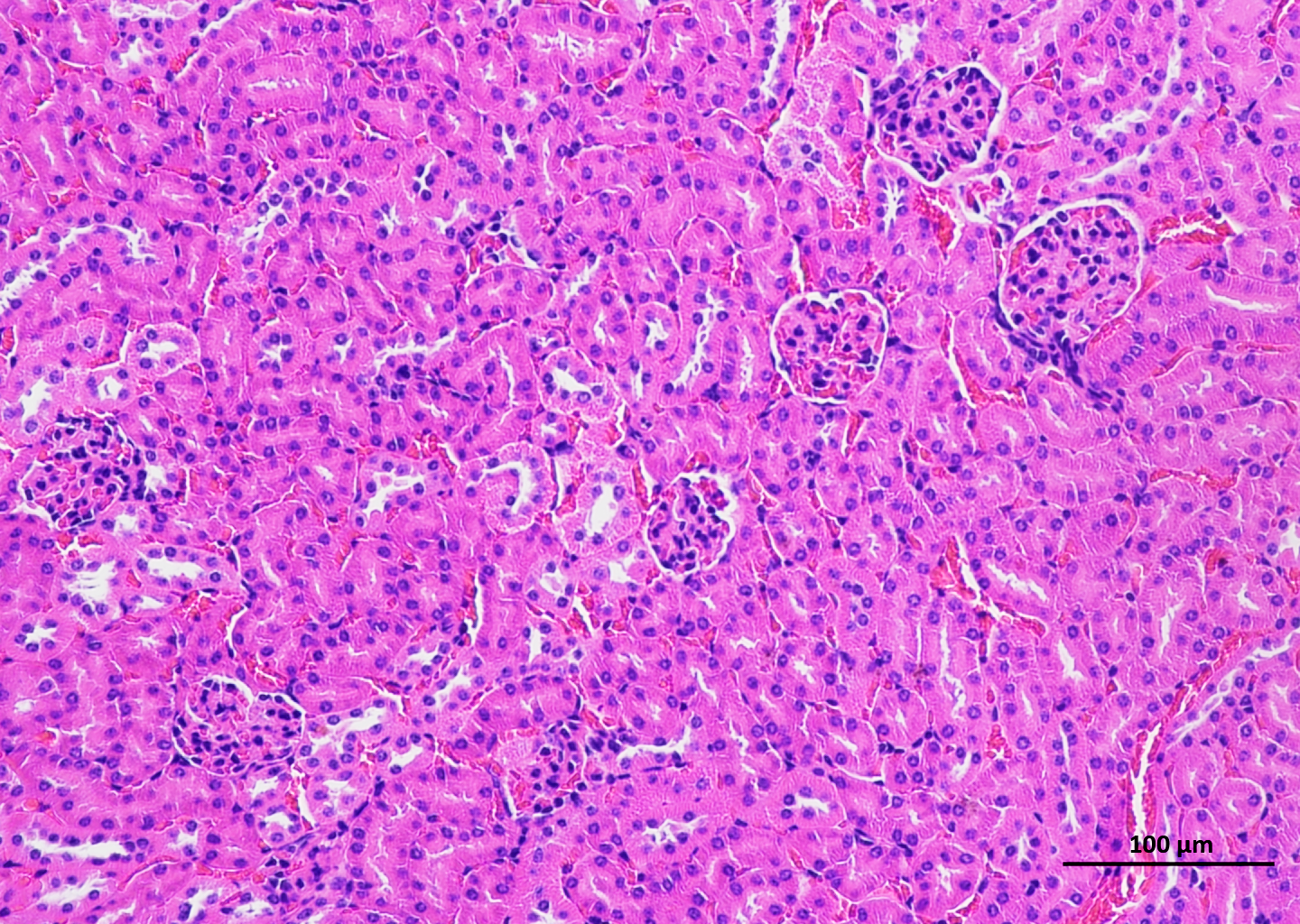

Supplement: Supplementary file 3 [file DataSheet4.zip › HE organ IDE-1 Original data/IDE-1-kidney.tif]

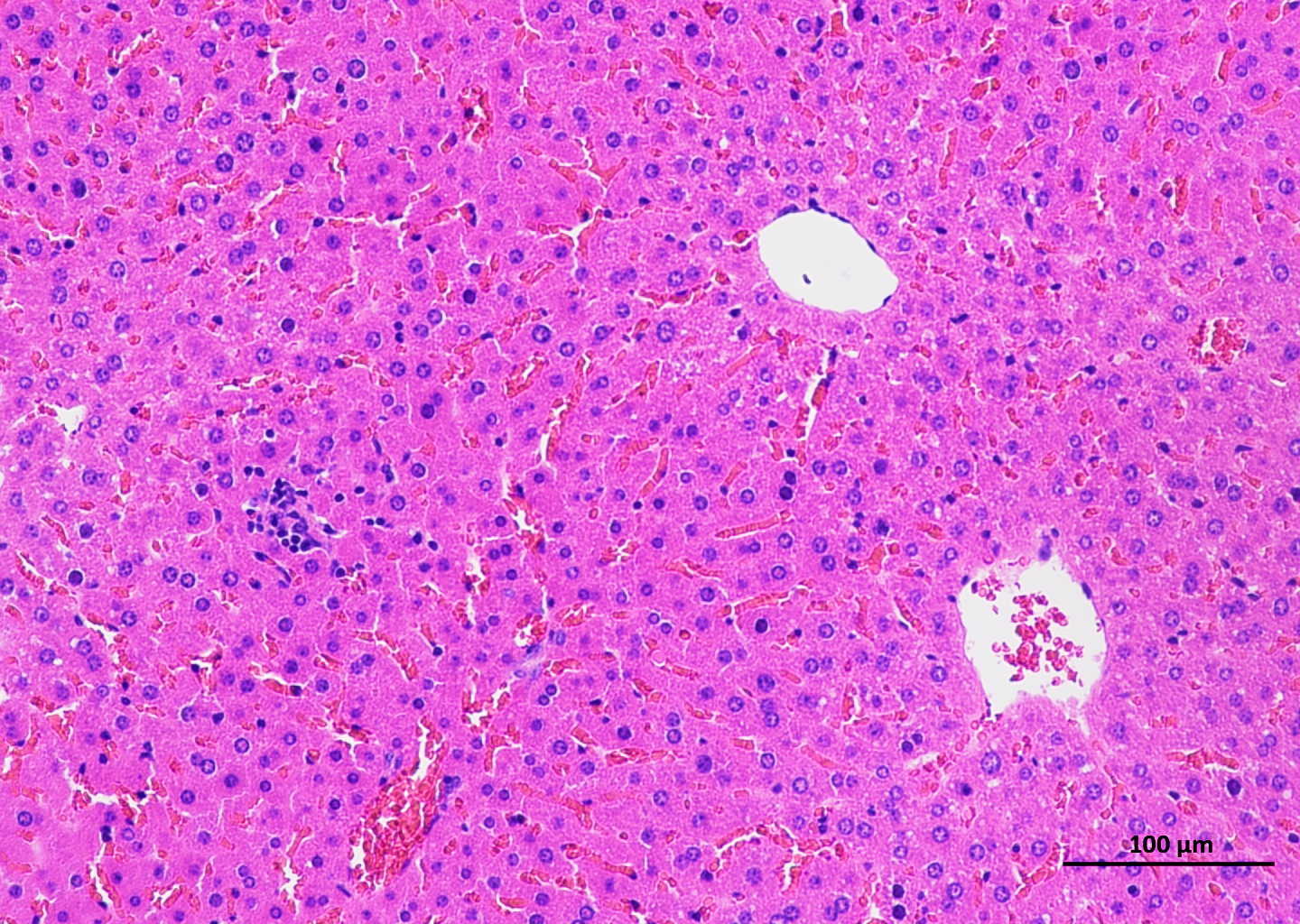

Supplement: Supplementary file 3 [file DataSheet4.zip › HE organ IDE-1 Original data/IDE-1-liver.tif]

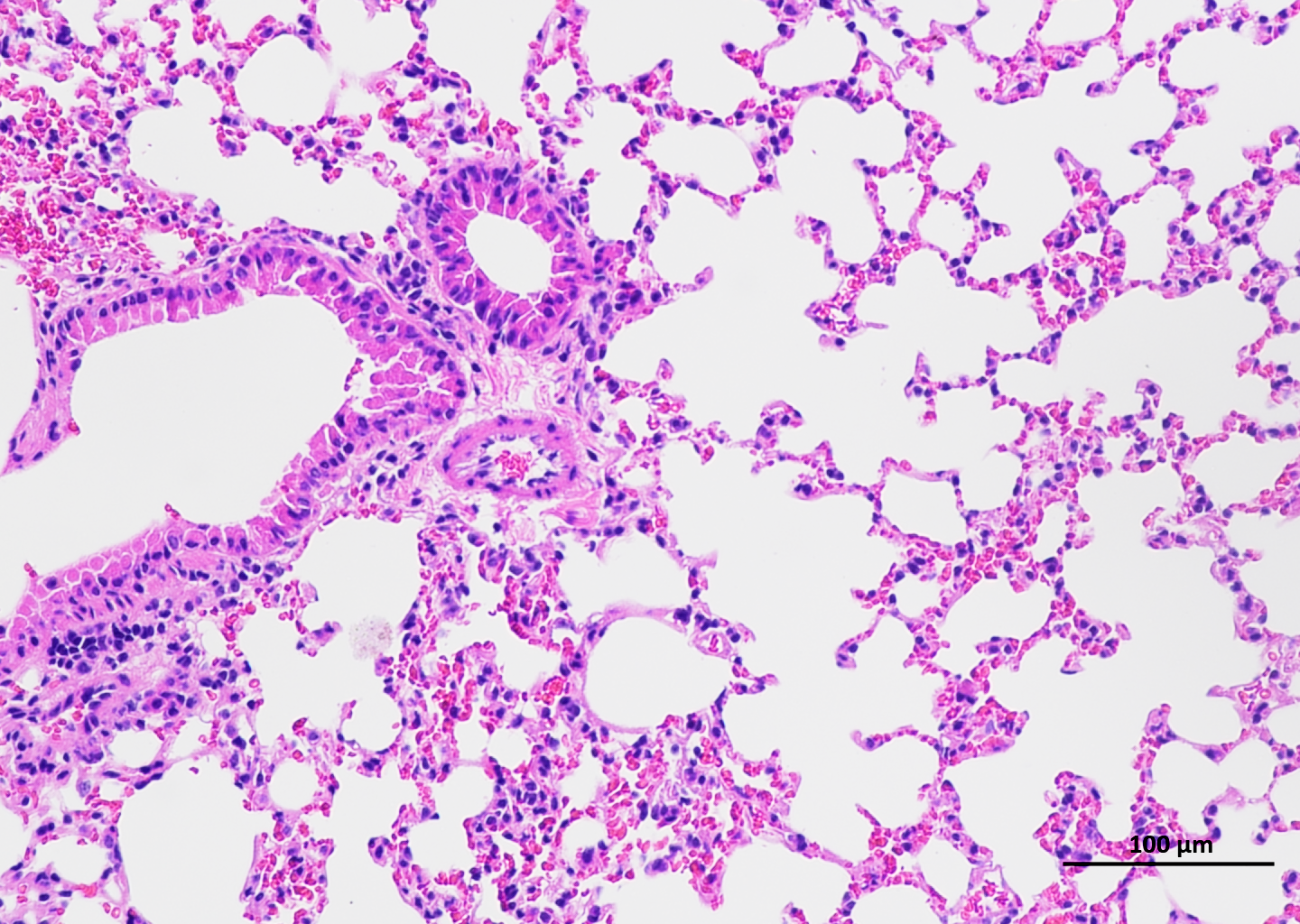

Supplement: Supplementary file 3 [file DataSheet4.zip › HE organ IDE-1 Original data/IDE-1-lung.tif]

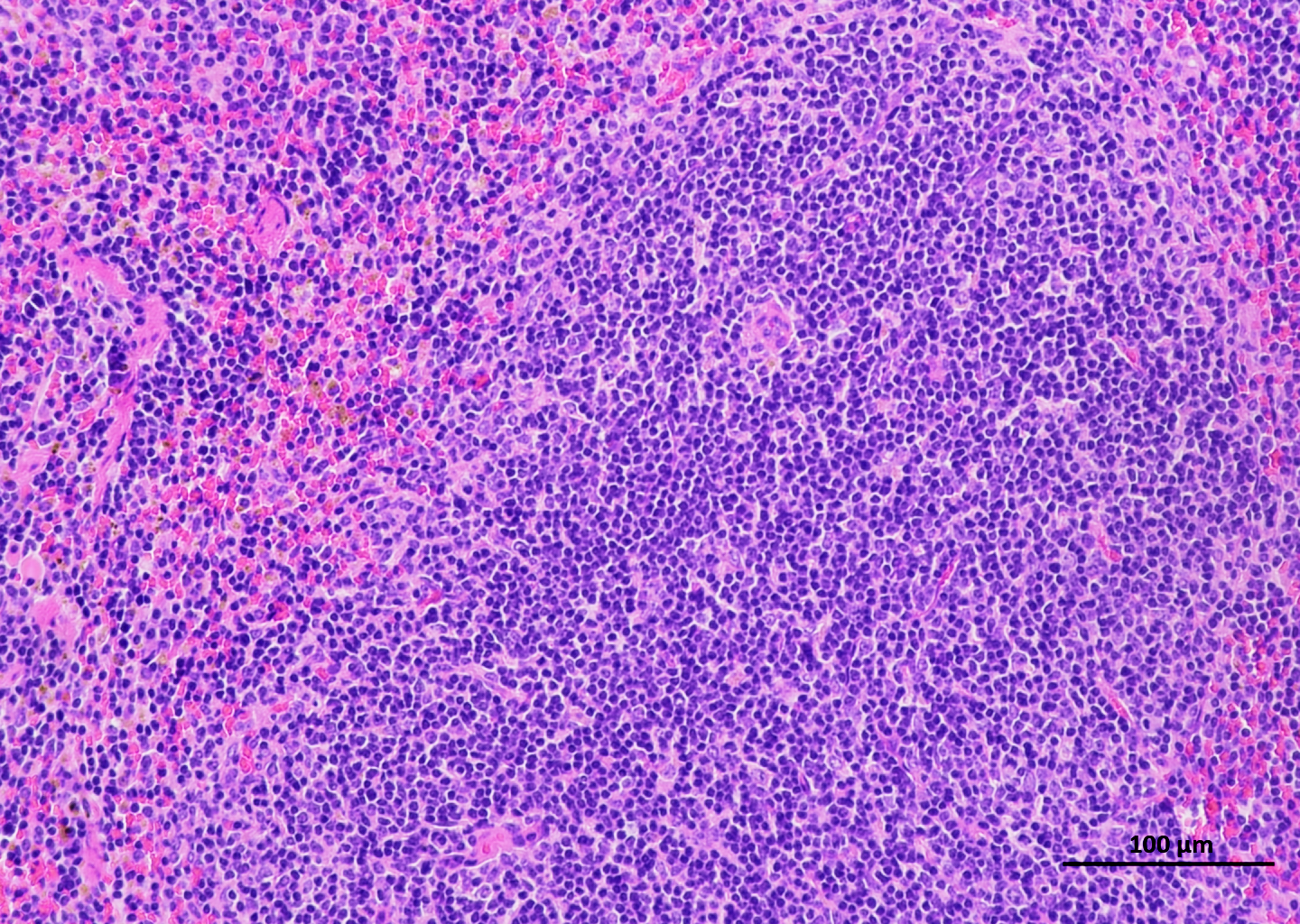

Supplement: Supplementary file 3 [file DataSheet4.zip › HE organ IDE-1 Original data/IDE-1-spleen.tif]

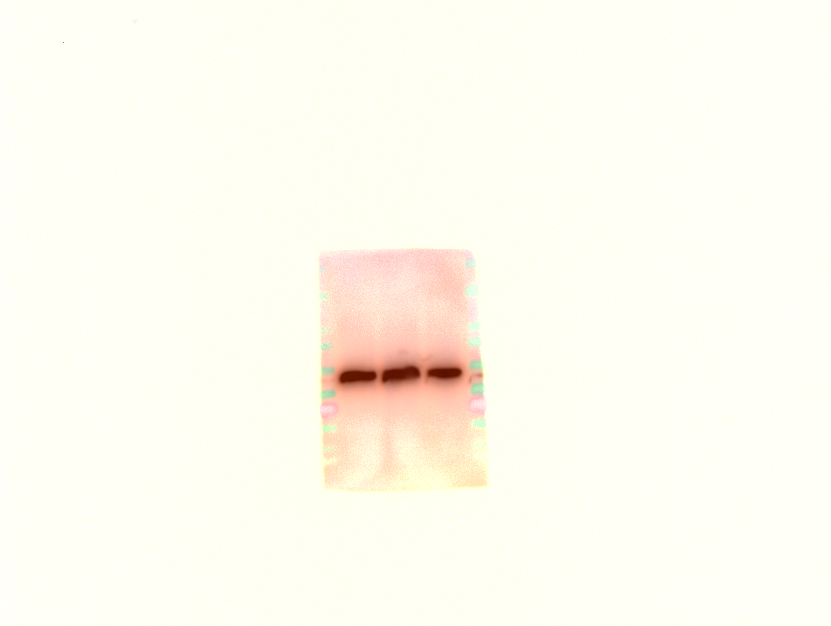

Supplement: Supplementary file 4 [file DataSheet1.zip › WB Original data/Bax/1 Actin with maker.png]

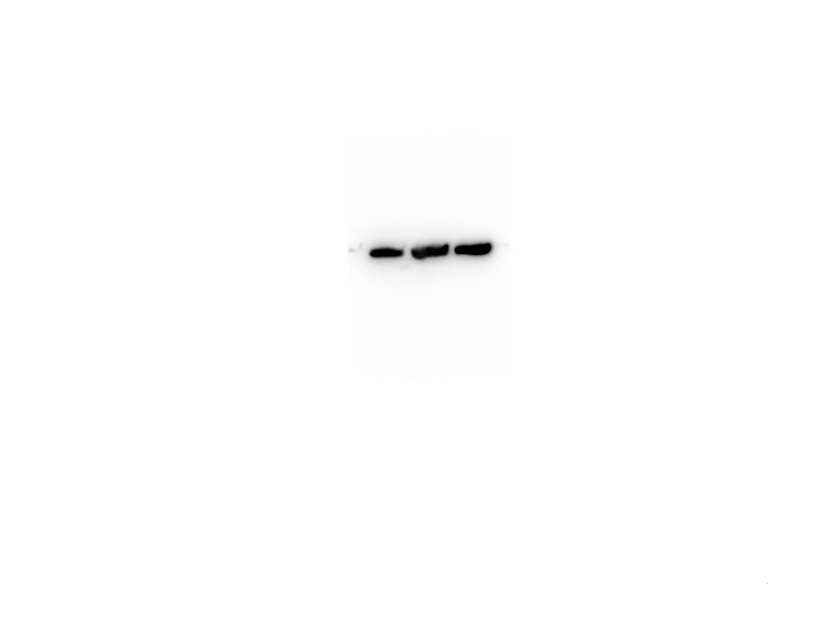

Supplement: Supplementary file 4 [file DataSheet1.zip › WB Original data/Bax/1 Actin.png]

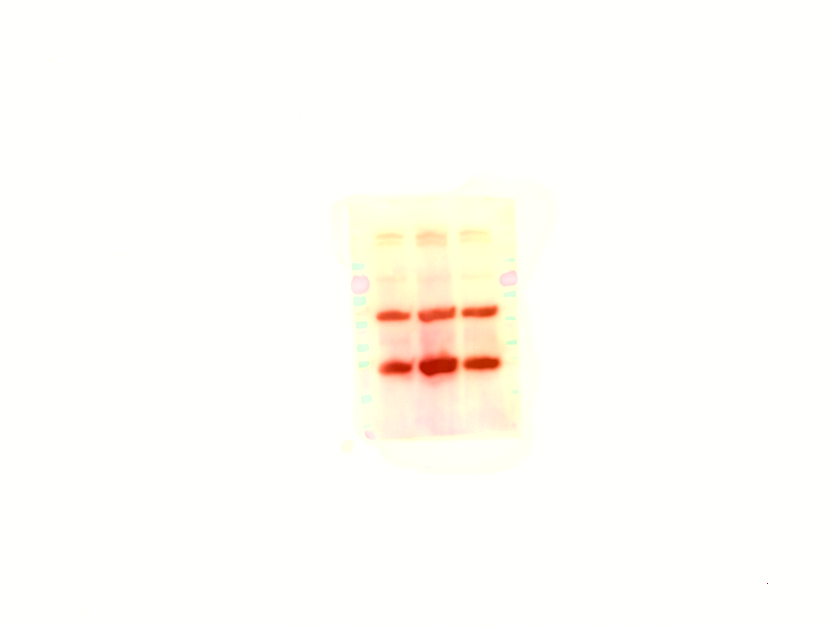

Supplement: Supplementary file 4 [file DataSheet1.zip › WB Original data/Bax/1 BAX with maker.png]

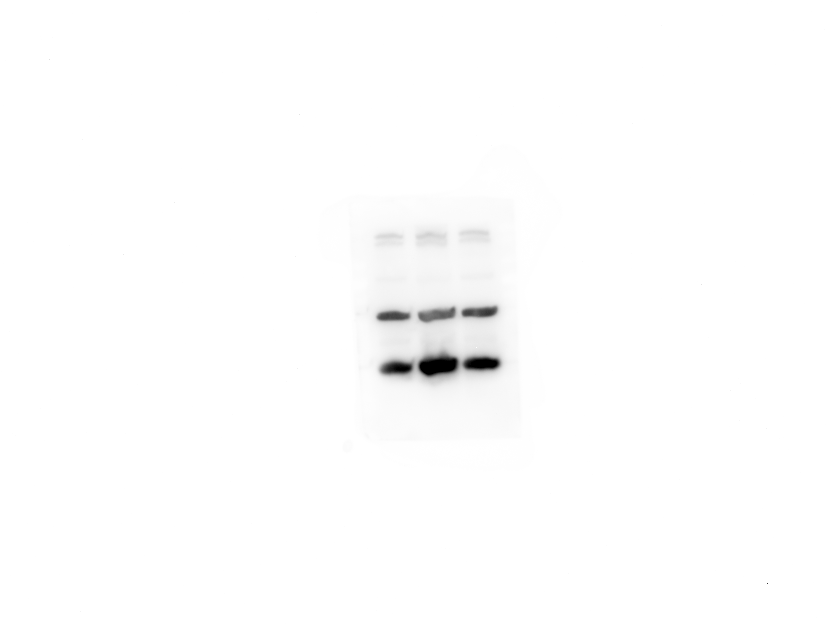

Supplement: Supplementary file 4 [file DataSheet1.zip › WB Original data/Bax/1 BAX.png]

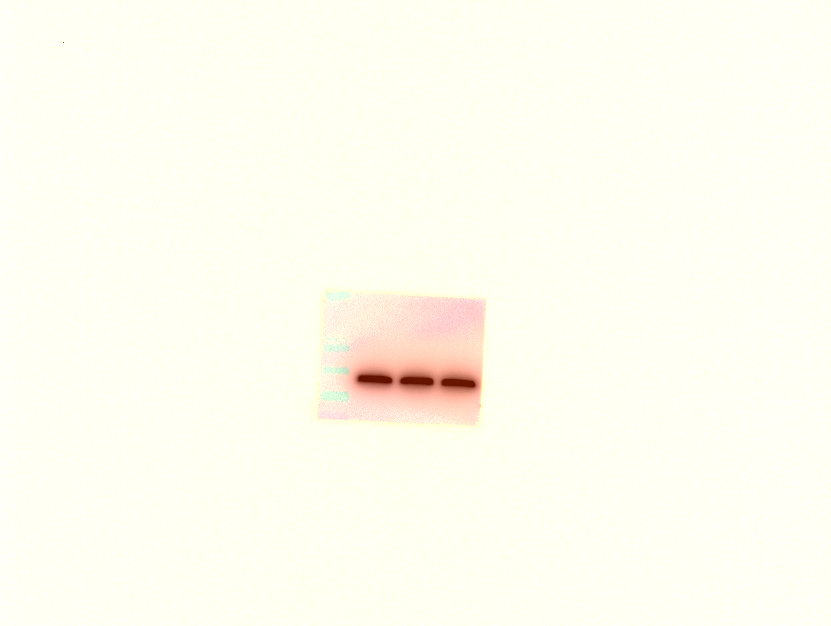

Supplement: Supplementary file 4 [file DataSheet1.zip › WB Original data/Bax/2 Actin with maker.png]

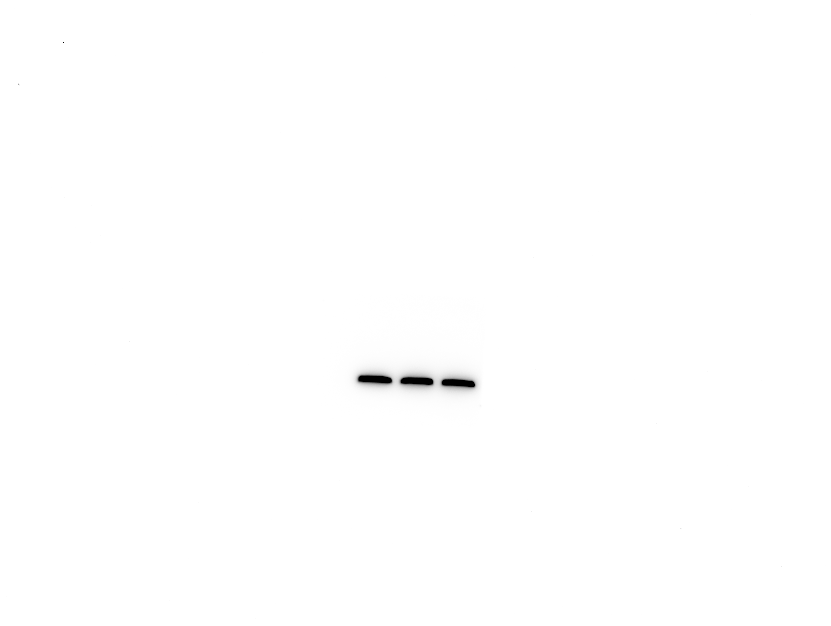

Supplement: Supplementary file 4 [file DataSheet1.zip › WB Original data/Bax/2 Actin.png]

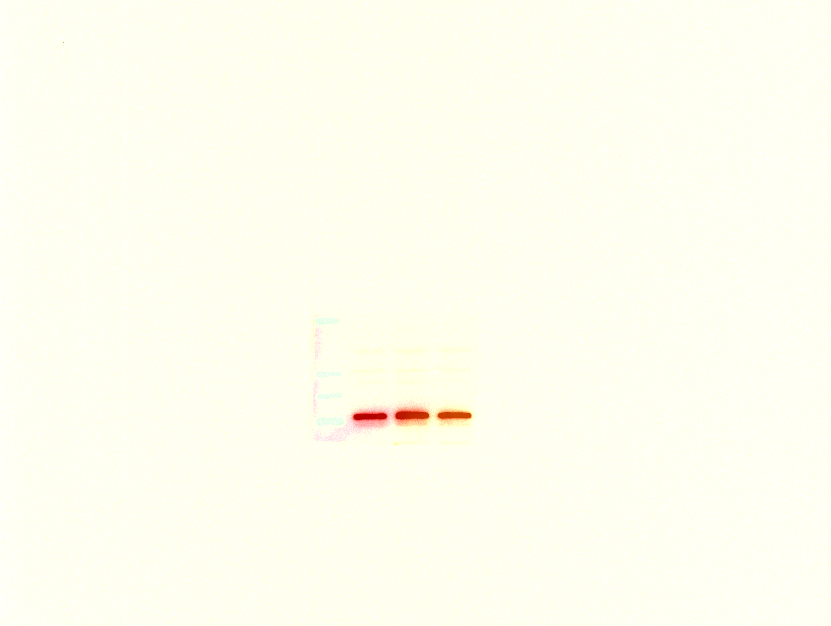

Supplement: Supplementary file 4 [file DataSheet1.zip › WB Original data/Bax/2 BAX with maker.png]

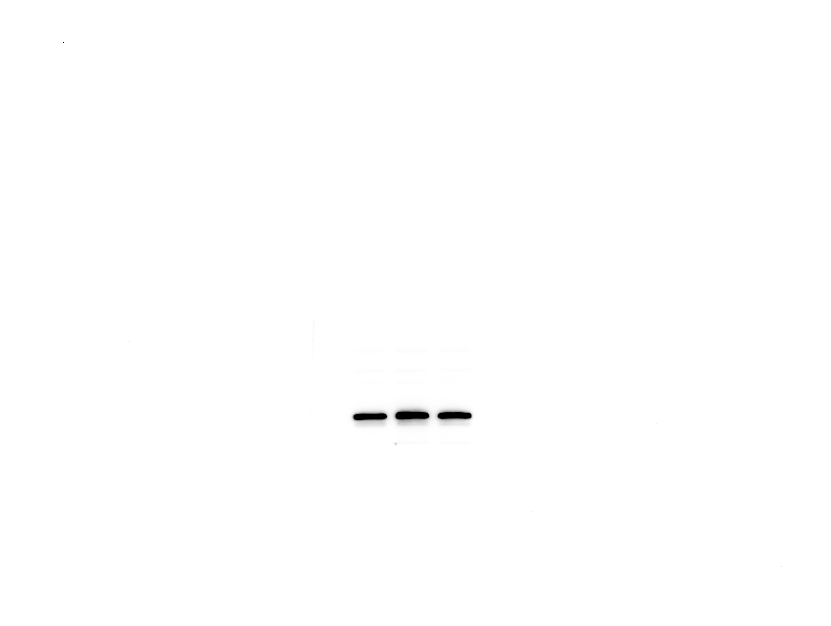

Supplement: Supplementary file 4 [file DataSheet1.zip › WB Original data/Bax/2 BAX.png]

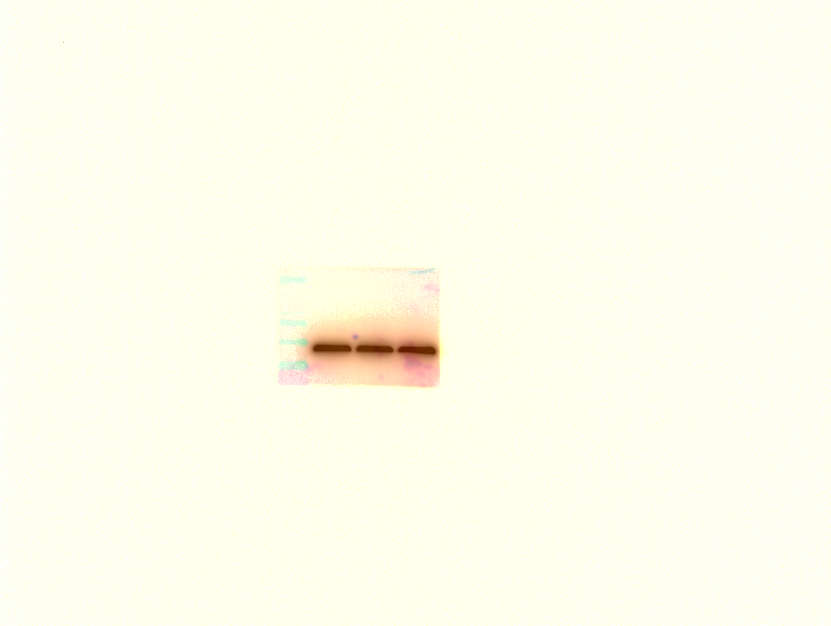

Supplement: Supplementary file 4 [file DataSheet1.zip › WB Original data/Bax/3 Actin with maker.png]

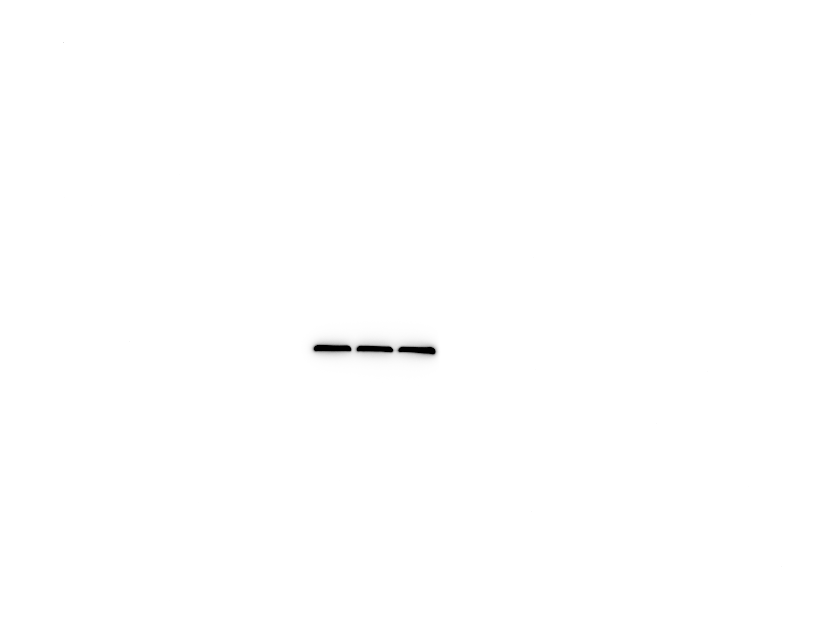

Supplement: Supplementary file 4 [file DataSheet1.zip › WB Original data/Bax/3 Actin.png]

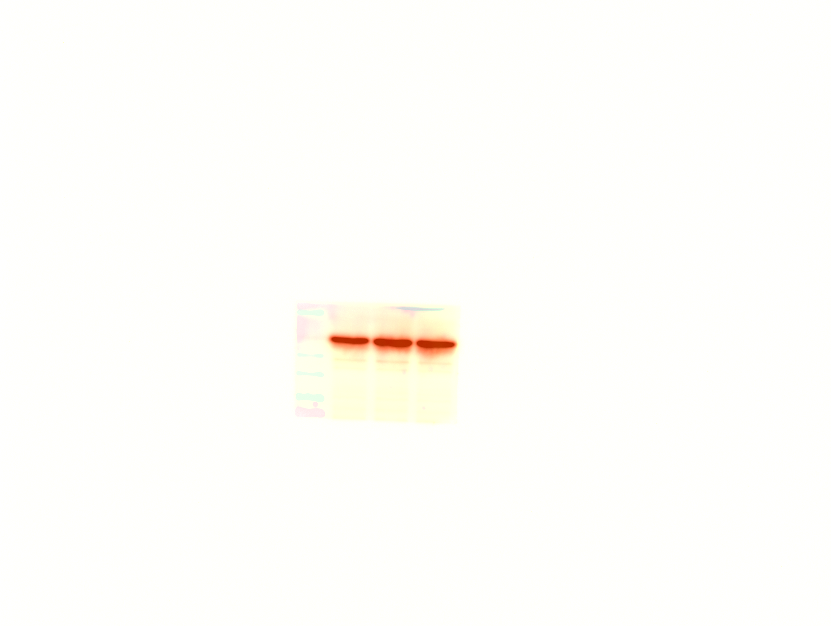

Supplement: Supplementary file 4 [file DataSheet1.zip › WB Original data/Bax/3 BAX with maker.png]

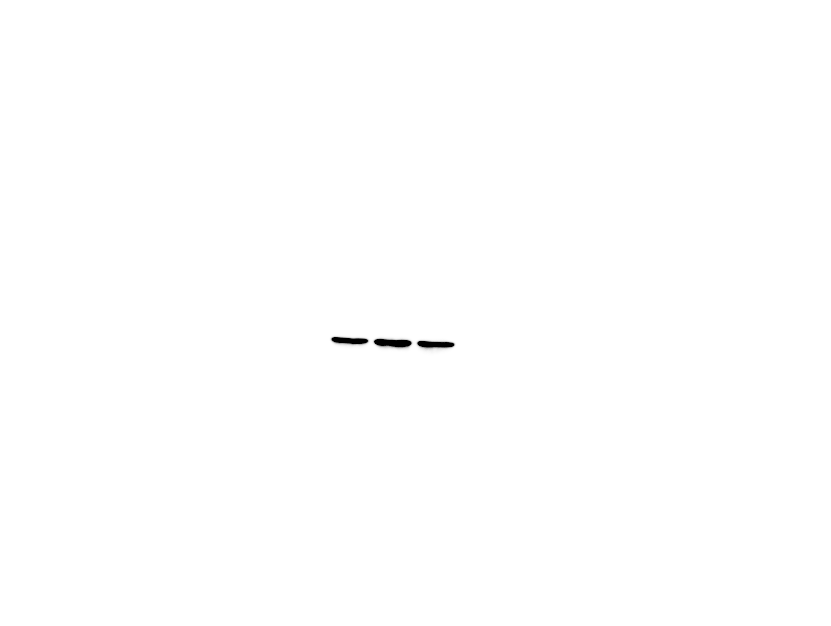

Supplement: Supplementary file 4 [file DataSheet1.zip › WB Original data/Bax/3 BAX.png]

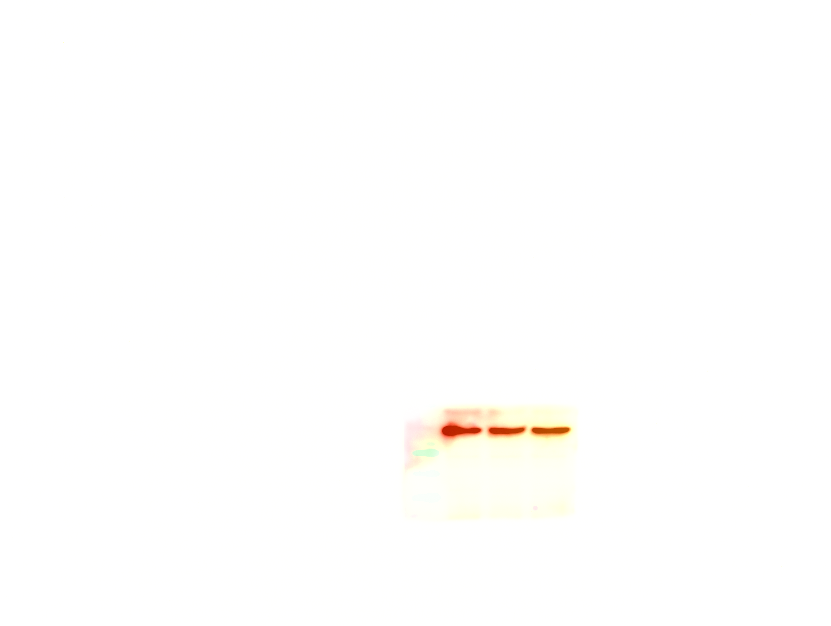

Supplement: Supplementary file 4 [file DataSheet1.zip › WB Original data/BCL-2/1 Actin with maker.png]

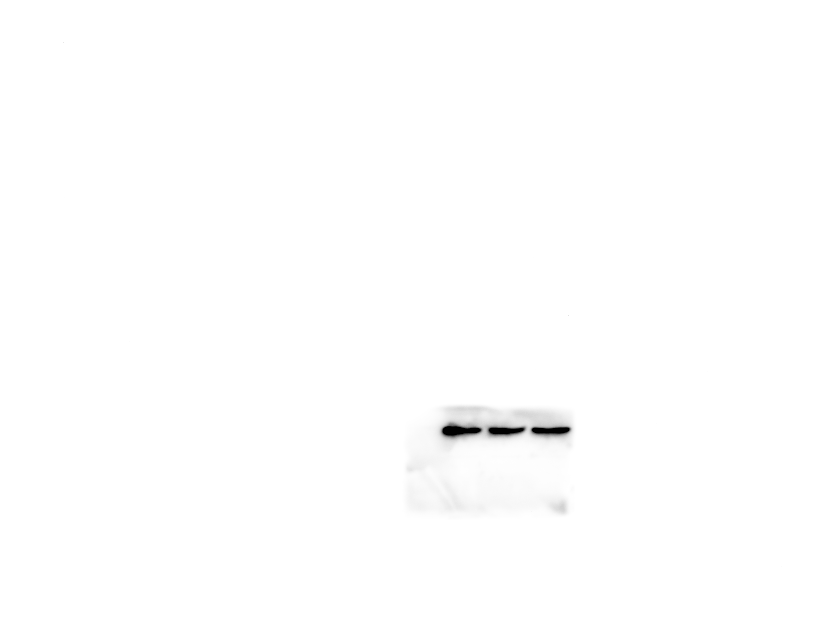

Supplement: Supplementary file 4 [file DataSheet1.zip › WB Original data/BCL-2/1 Actin.png]

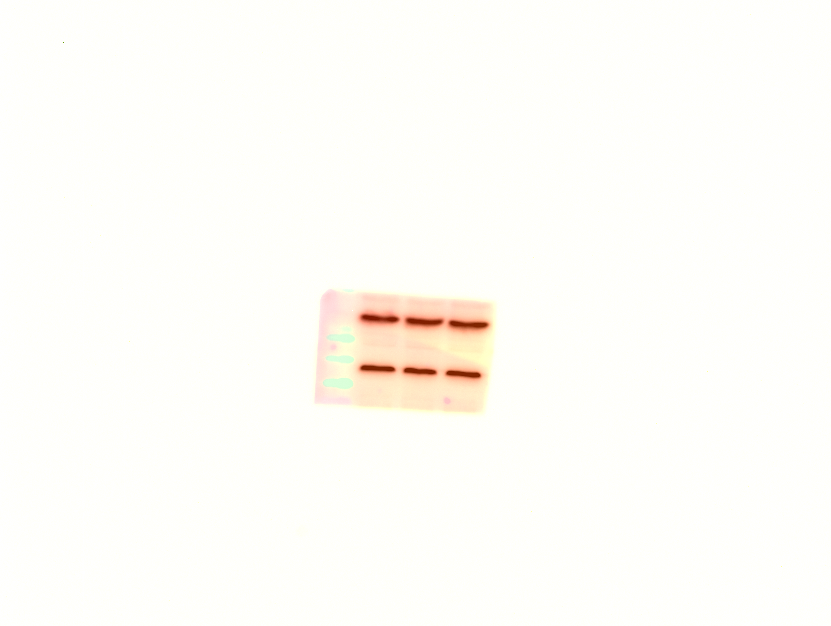

Supplement: Supplementary file 4 [file DataSheet1.zip › WB Original data/BCL-2/1 Bcl-2 with maker.png]

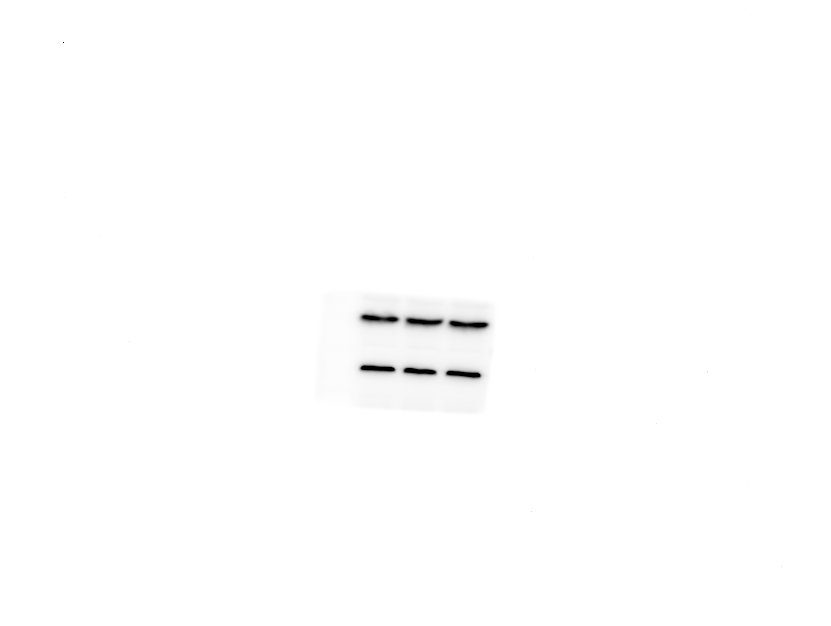

Supplement: Supplementary file 4 [file DataSheet1.zip › WB Original data/BCL-2/1 Bcl-2.png]

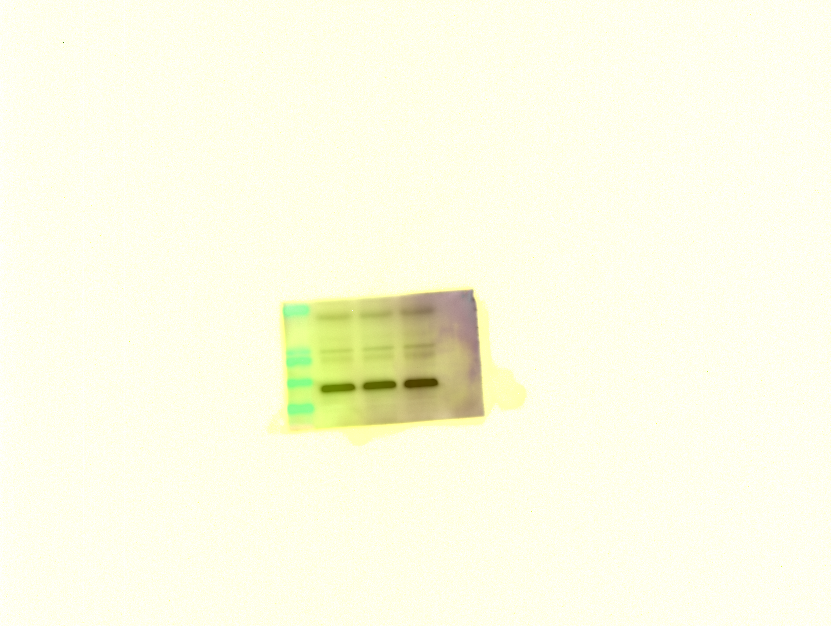

Supplement: Supplementary file 4 [file DataSheet1.zip › WB Original data/BCL-2/2 Actin with maker.png]

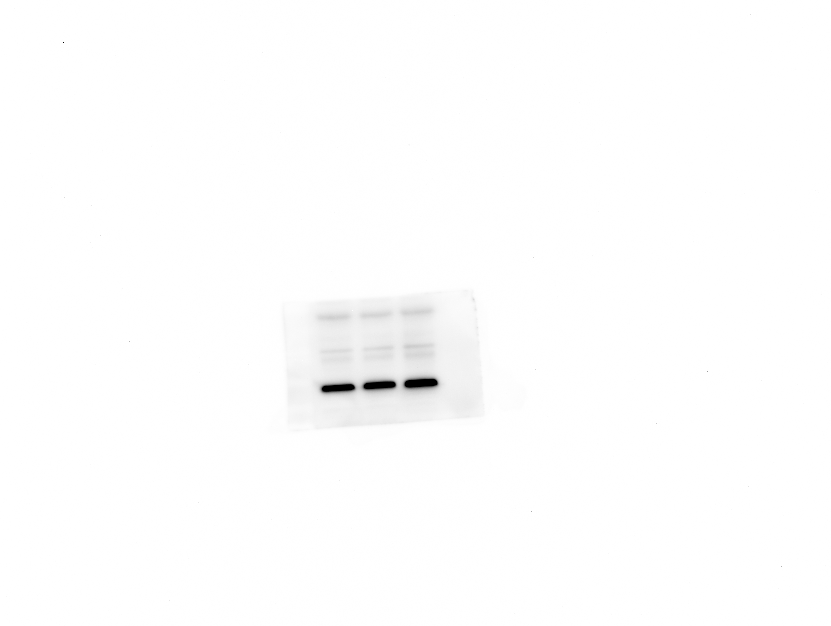

Supplement: Supplementary file 4 [file DataSheet1.zip › WB Original data/BCL-2/2 Actin.png]

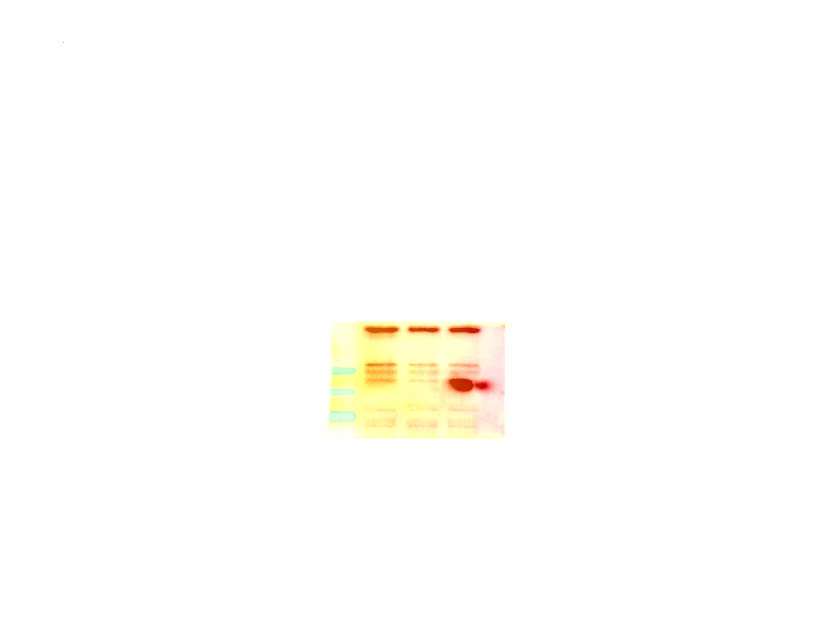

Supplement: Supplementary file 4 [file DataSheet1.zip › WB Original data/BCL-2/2 Bcl-2 with maker.png]

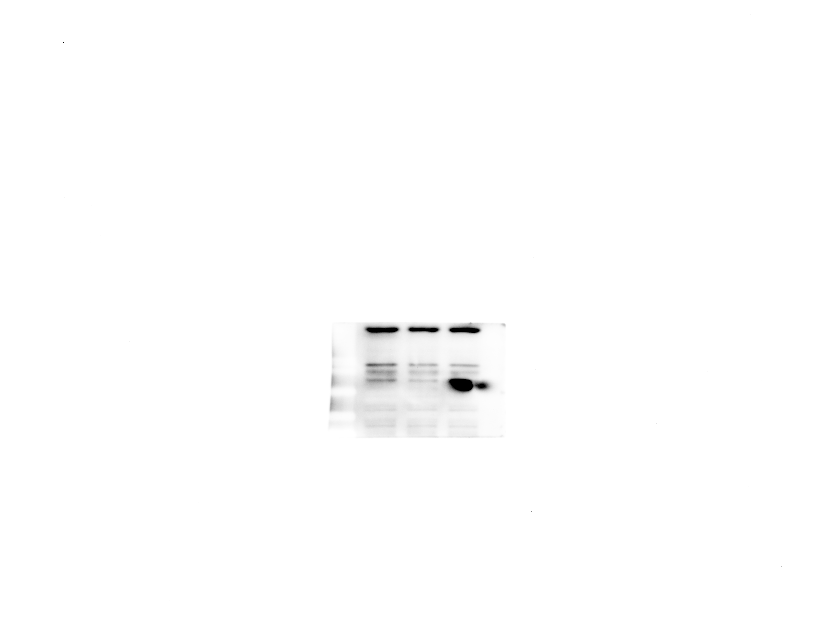

Supplement: Supplementary file 4 [file DataSheet1.zip › WB Original data/BCL-2/2 Bcl-2.png]

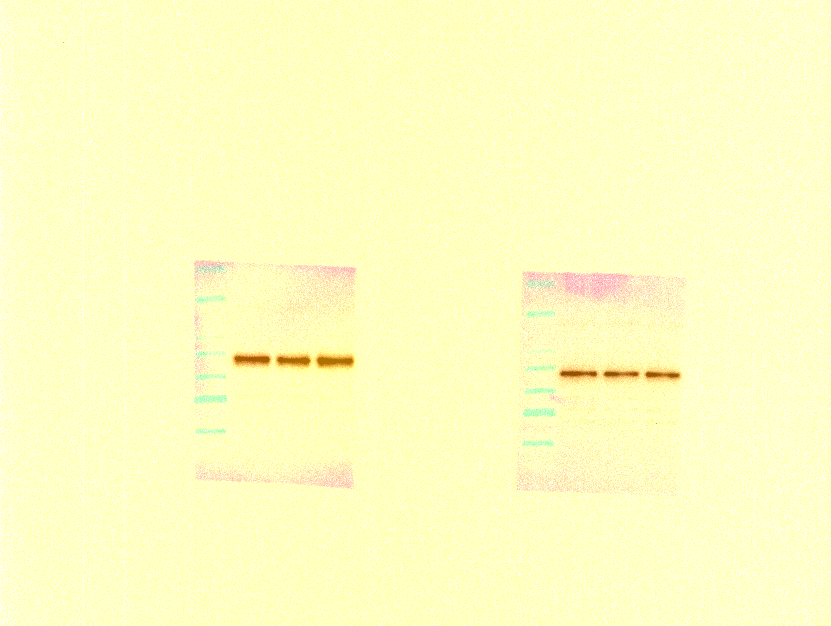

Supplement: Supplementary file 4 [file DataSheet1.zip › WB Original data/BCL-2/3 Actin and Bcl-2 with maker.png]

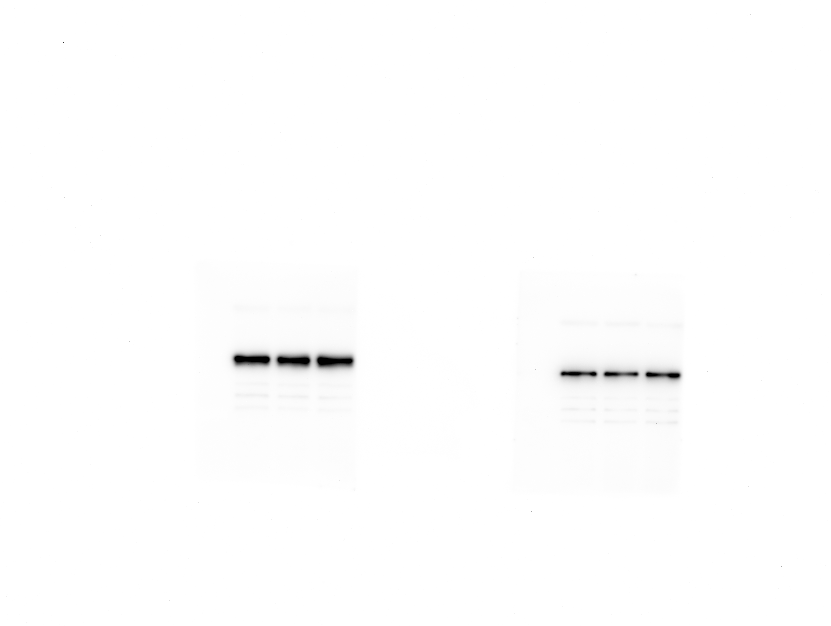

Supplement: Supplementary file 4 [file DataSheet1.zip › WB Original data/BCL-2/3 Actin and Bcl-2.png]

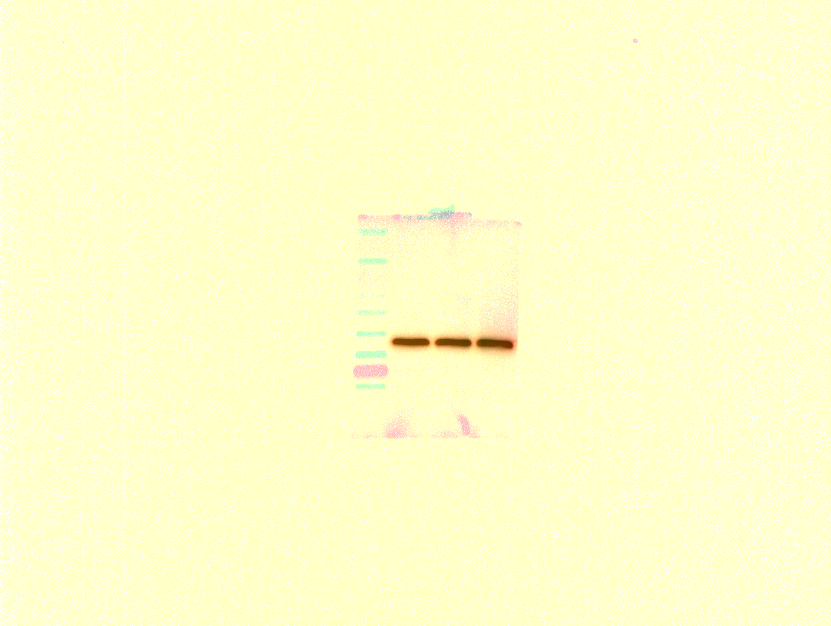

Supplement: Supplementary file 4 [file DataSheet1.zip › WB Original data/Caspase 3/1 Actin with maker.png]

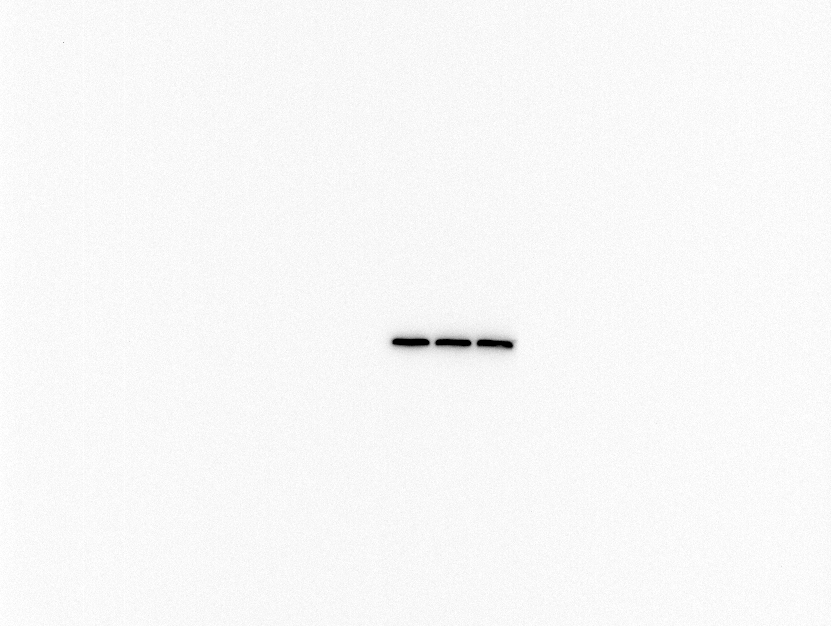

Supplement: Supplementary file 4 [file DataSheet1.zip › WB Original data/Caspase 3/1 Actin.png]

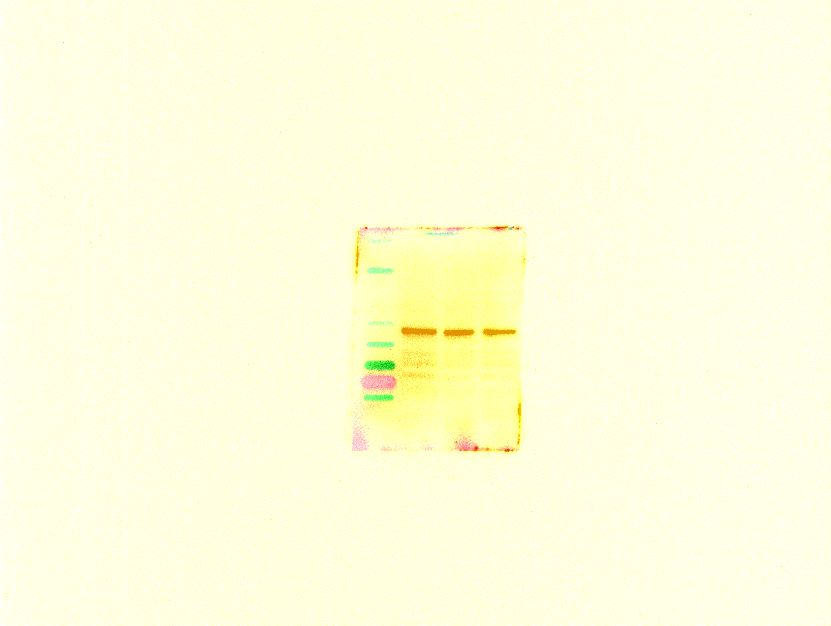

Supplement: Supplementary file 4 [file DataSheet1.zip › WB Original data/Caspase 3/1 caspase 3 with maker.png]

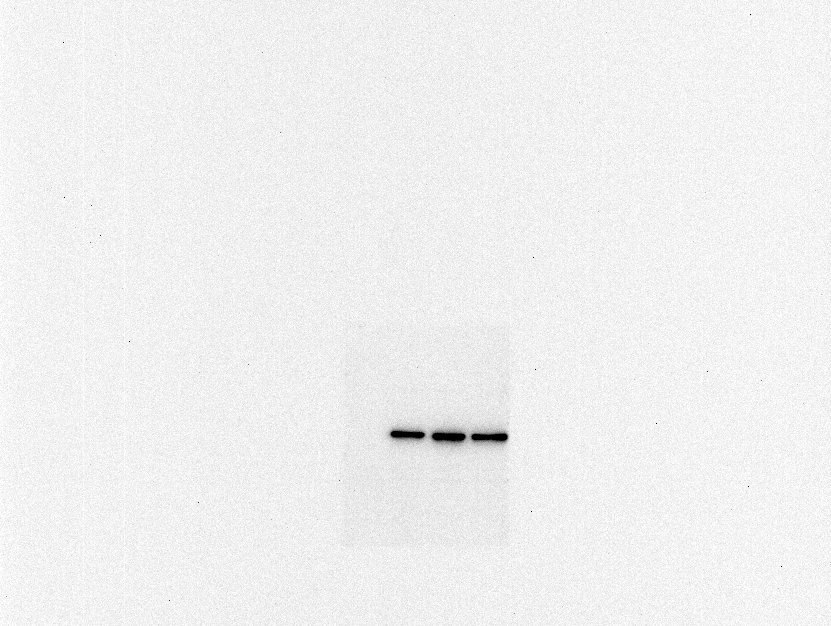

Supplement: Supplementary file 4 [file DataSheet1.zip › WB Original data/Caspase 3/1 caspase 3.png]

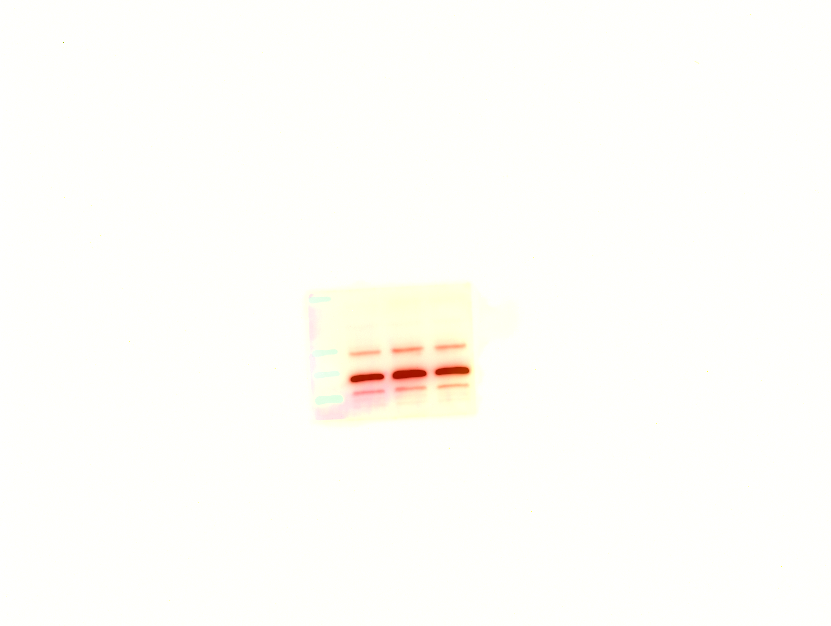

Supplement: Supplementary file 4 [file DataSheet1.zip › WB Original data/Caspase 3/2 Actin with maker.png]

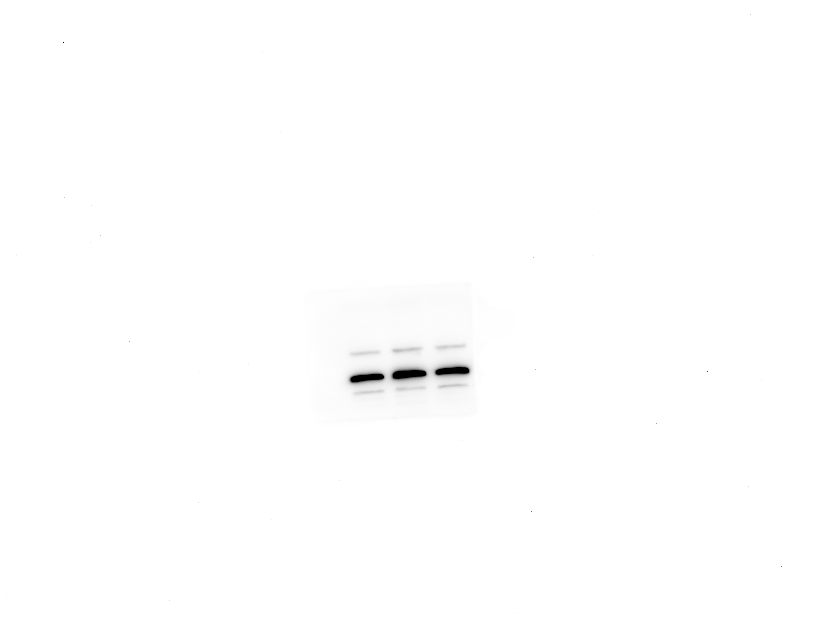

Supplement: Supplementary file 4 [file DataSheet1.zip › WB Original data/Caspase 3/2 Actin.png]

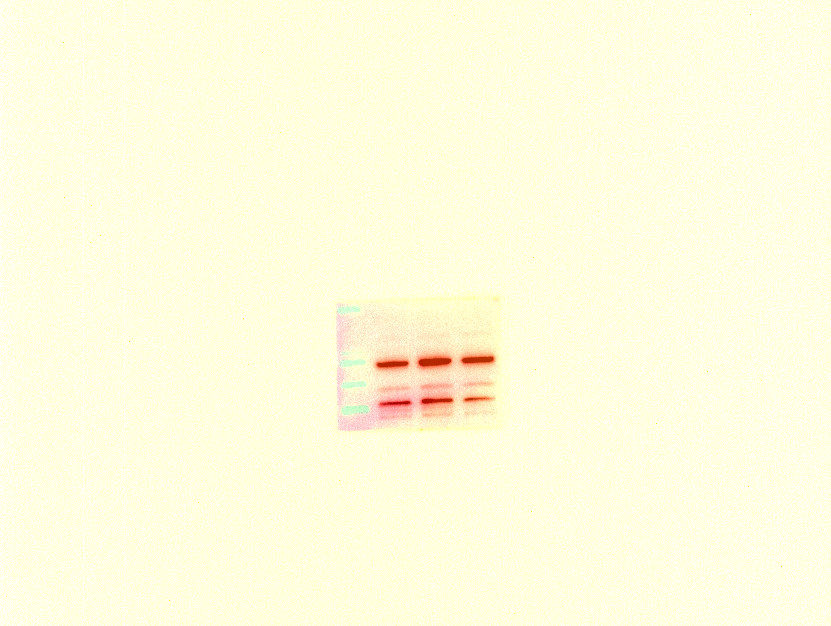

Supplement: Supplementary file 4 [file DataSheet1.zip › WB Original data/Caspase 3/2 caspase 3 with maker.png]

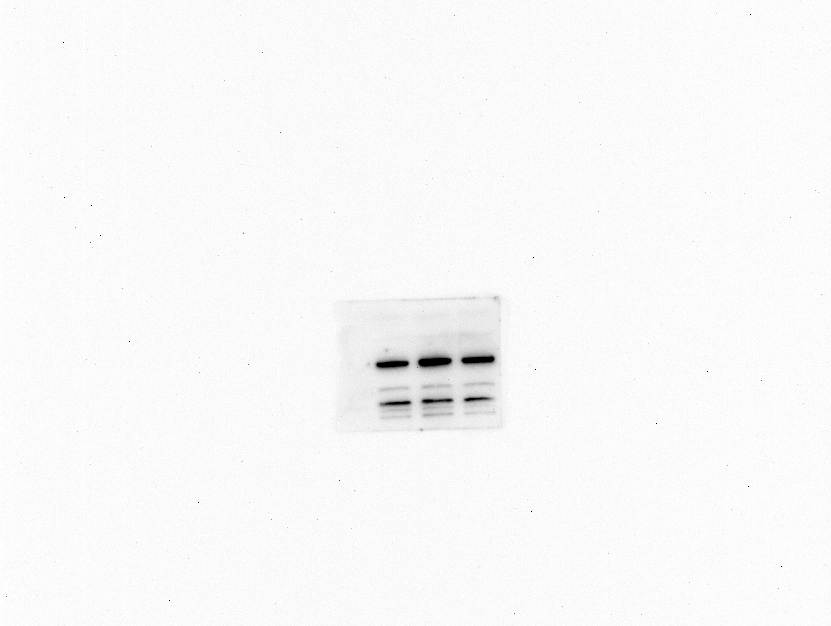

Supplement: Supplementary file 4 [file DataSheet1.zip › WB Original data/Caspase 3/2 caspase 3.png]

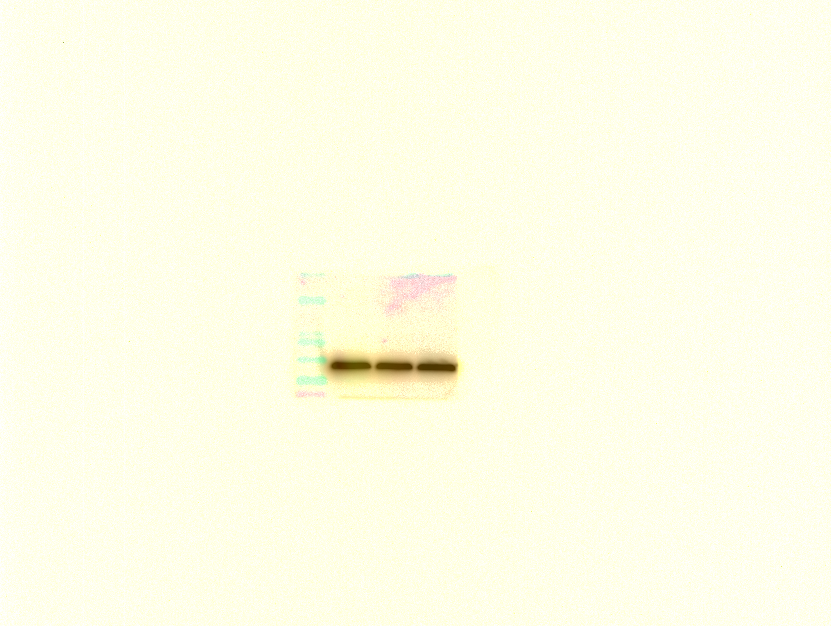

Supplement: Supplementary file 4 [file DataSheet1.zip › WB Original data/Caspase 3/3 Actin with maker.png]

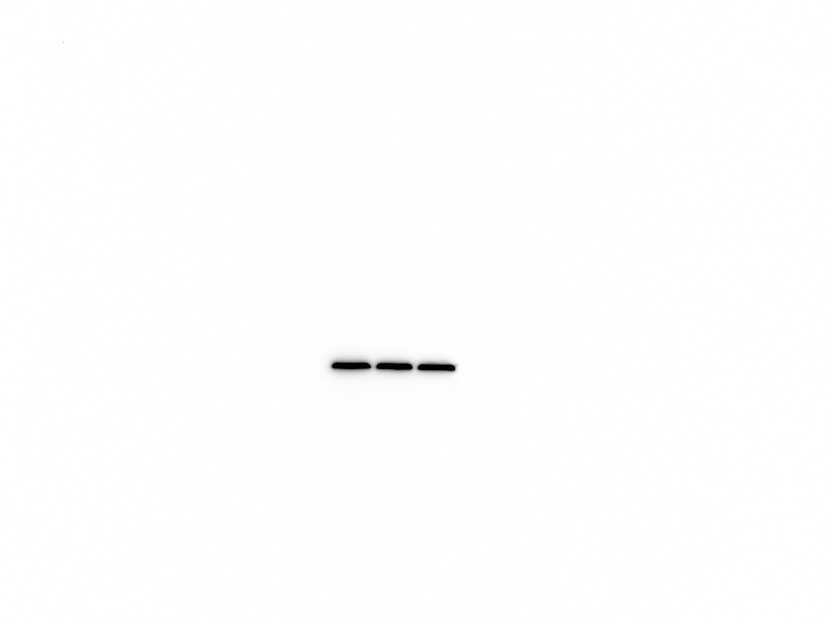

Supplement: Supplementary file 4 [file DataSheet1.zip › WB Original data/Caspase 3/3 Actin.png]

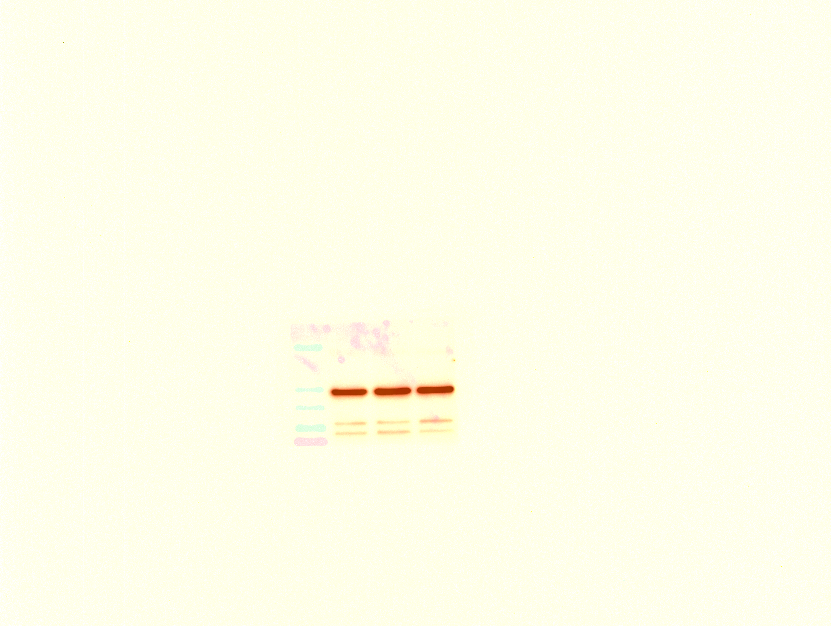

Supplement: Supplementary file 4 [file DataSheet1.zip › WB Original data/Caspase 3/3 caspase 3 with maker.png]

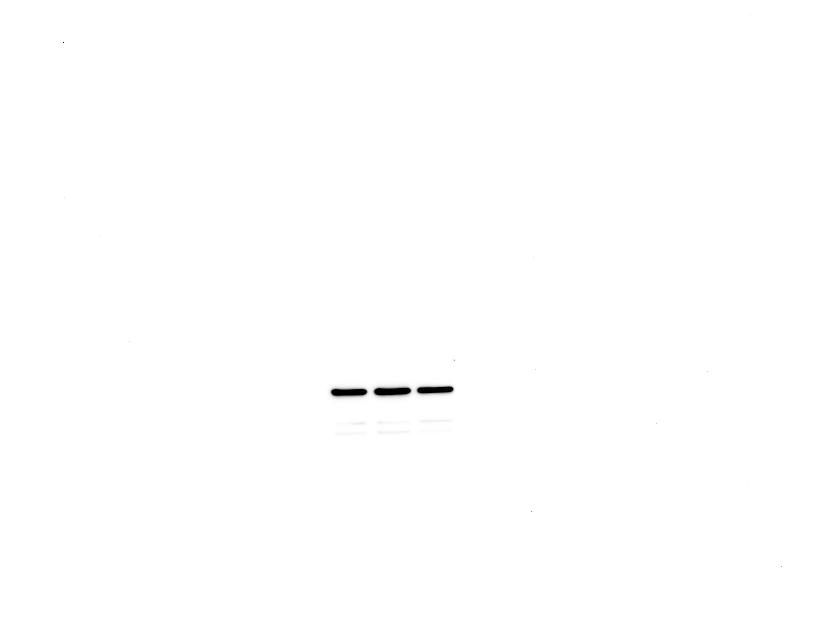

Supplement: Supplementary file 4 [file DataSheet1.zip › WB Original data/Caspase 3/3 caspase 3.png]

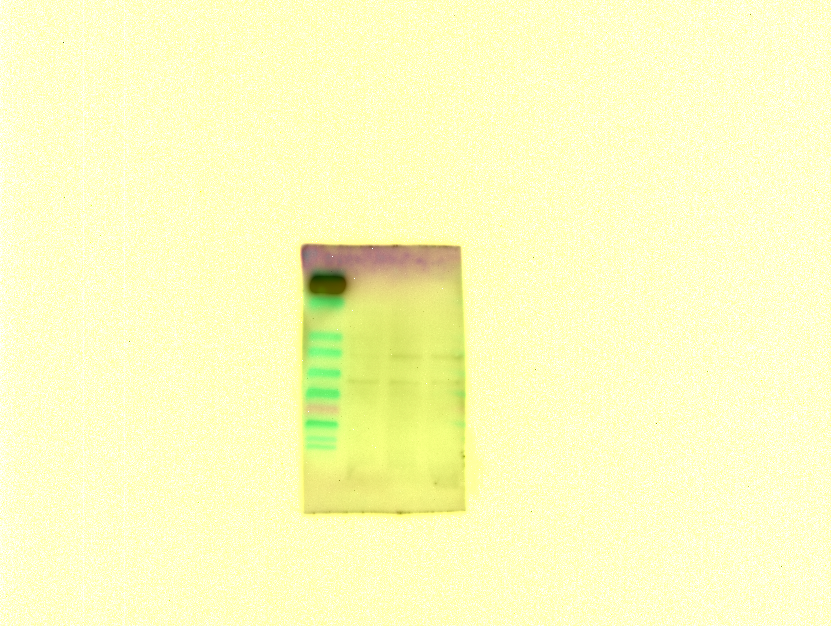

Supplement: Supplementary file 4 [file DataSheet1.zip › WB Original data/HO-1/1 Actin with maker.png]

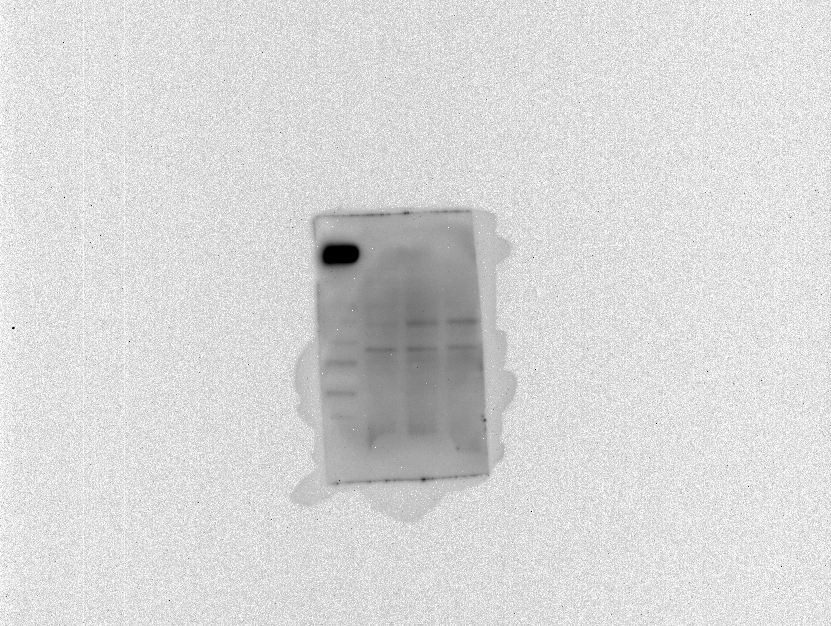

Supplement: Supplementary file 4 [file DataSheet1.zip › WB Original data/HO-1/1 Actin.png]

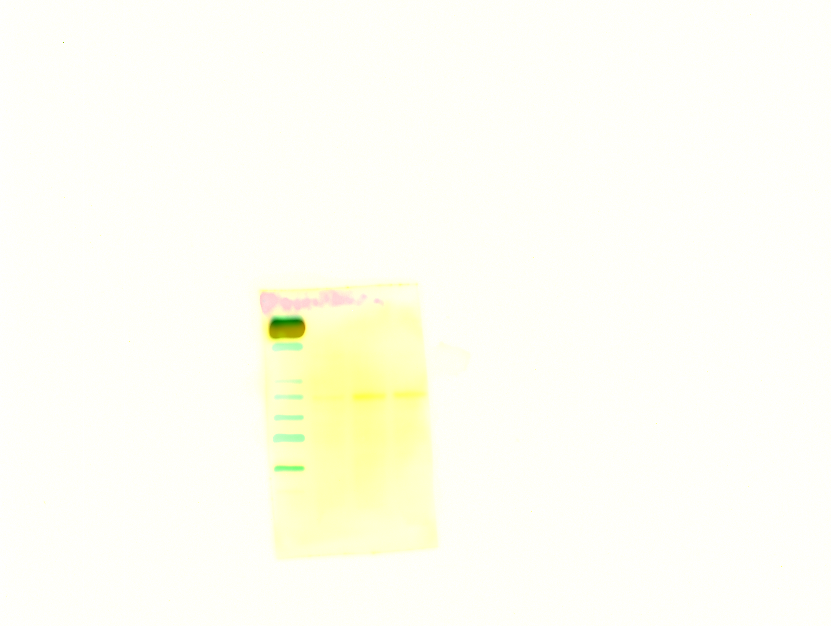

Supplement: Supplementary file 4 [file DataSheet1.zip › WB Original data/HO-1/1 HO-1 with maker.png]

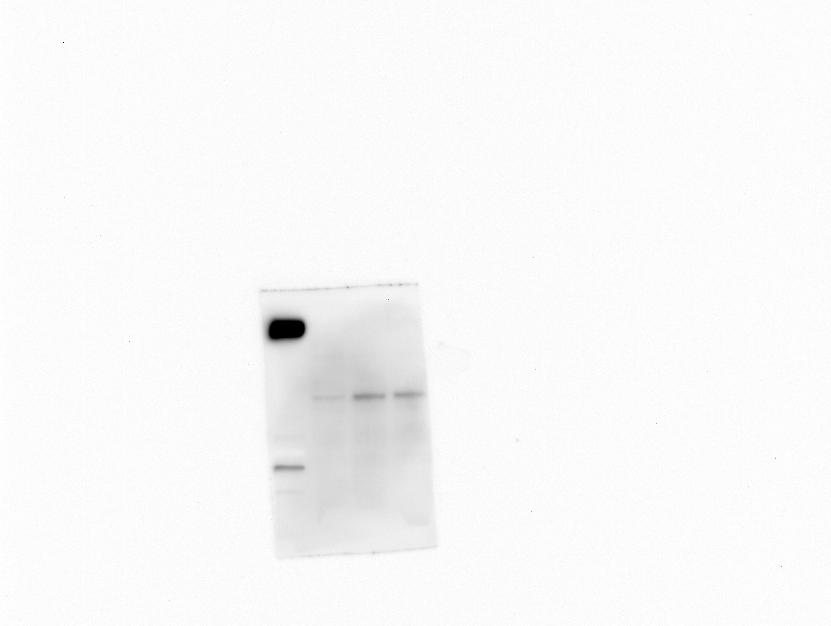

Supplement: Supplementary file 4 [file DataSheet1.zip › WB Original data/HO-1/1 HO-1.png]

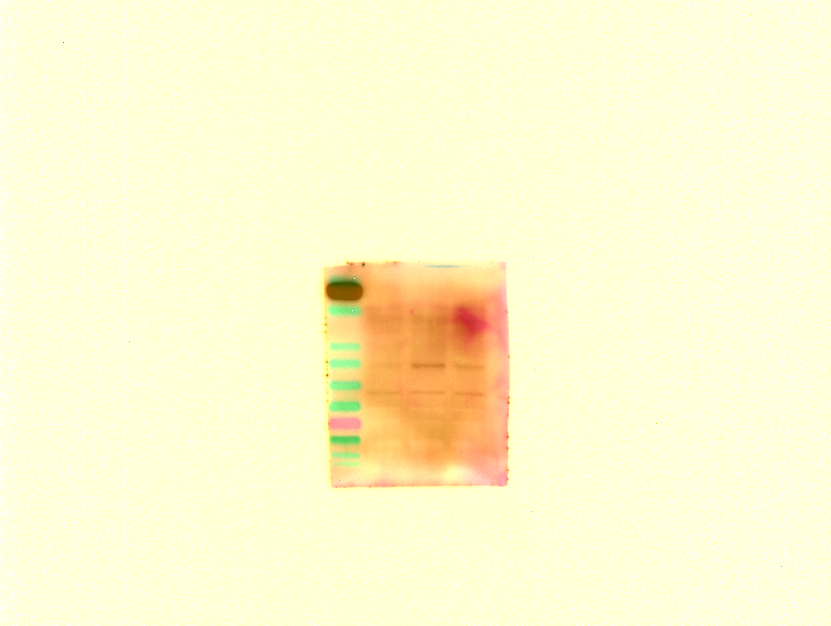

Supplement: Supplementary file 4 [file DataSheet1.zip › WB Original data/HO-1/2 Actin with maker.png]

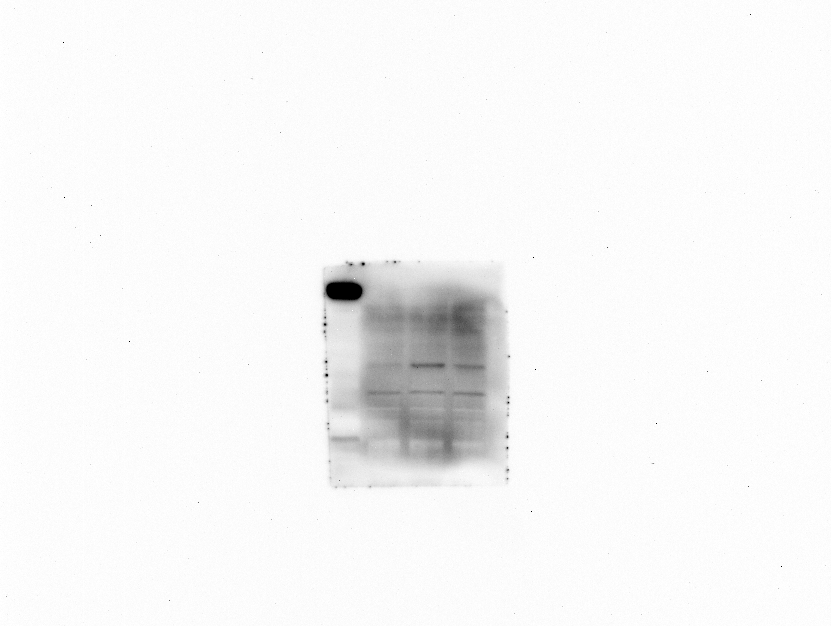

Supplement: Supplementary file 4 [file DataSheet1.zip › WB Original data/HO-1/2 Actin.png]

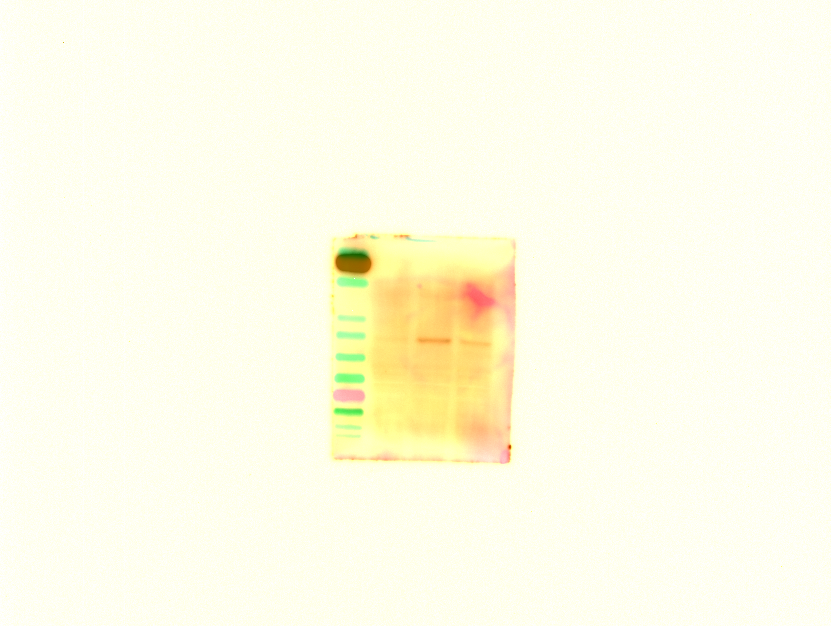

Supplement: Supplementary file 4 [file DataSheet1.zip › WB Original data/HO-1/2 HO-1 with maker.png]

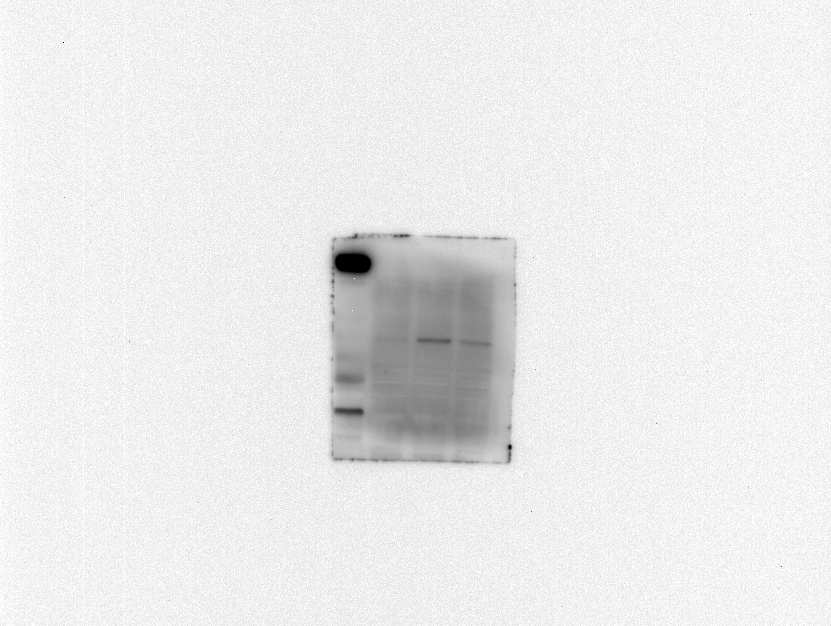

Supplement: Supplementary file 4 [file DataSheet1.zip › WB Original data/HO-1/2 HO-1.png]

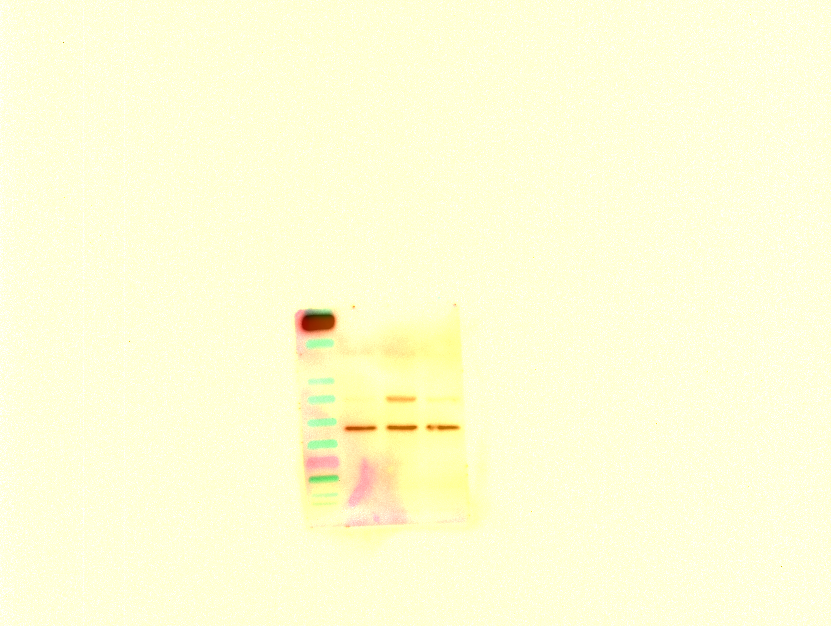

Supplement: Supplementary file 4 [file DataSheet1.zip › WB Original data/HO-1/3 Actin with maker.png]

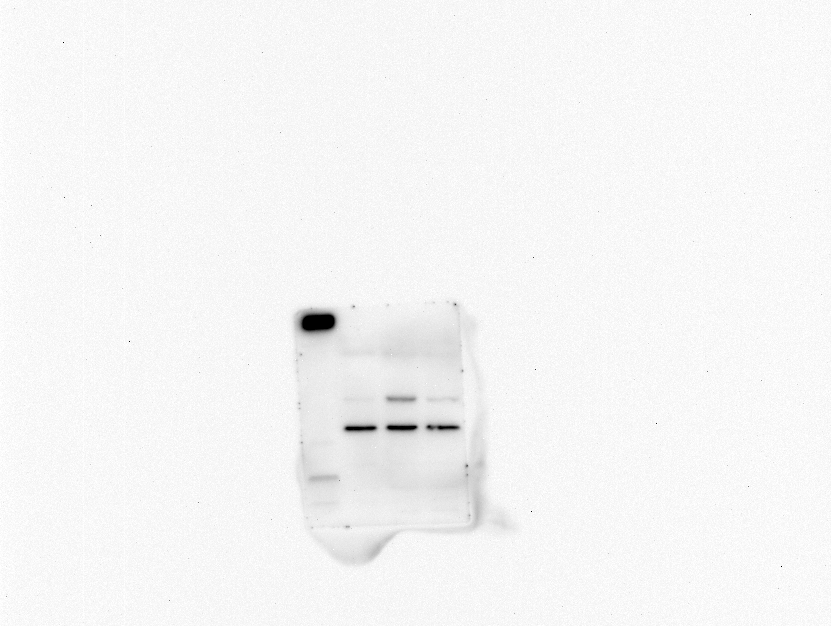

Supplement: Supplementary file 4 [file DataSheet1.zip › WB Original data/HO-1/3 Actin.png]

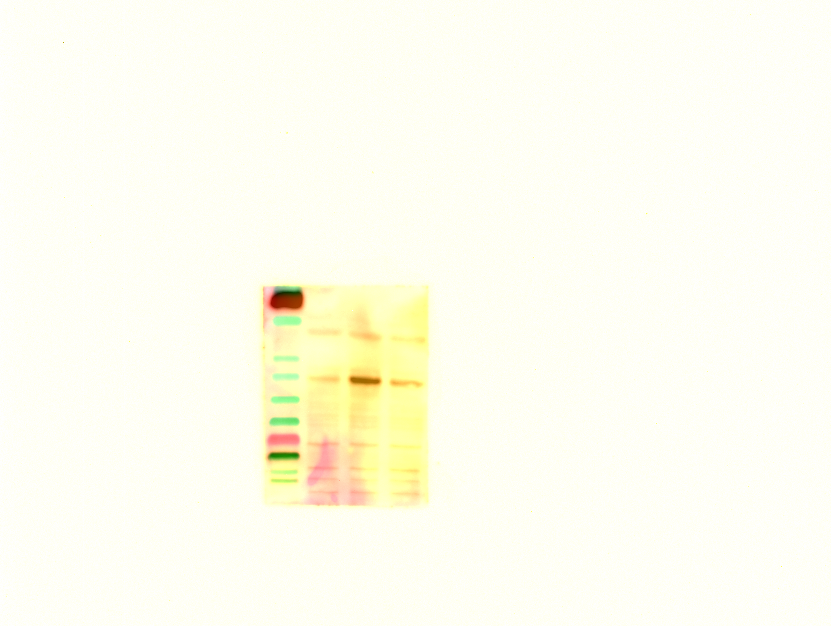

Supplement: Supplementary file 4 [file DataSheet1.zip › WB Original data/HO-1/3 HO-1 with maker.png]

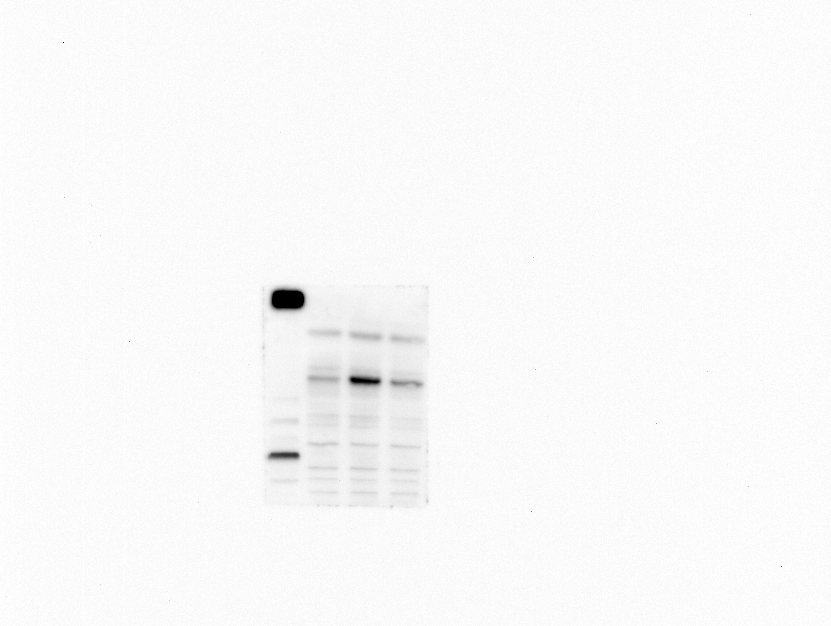

Supplement: Supplementary file 4 [file DataSheet1.zip › WB Original data/HO-1/3 HO-1.png]

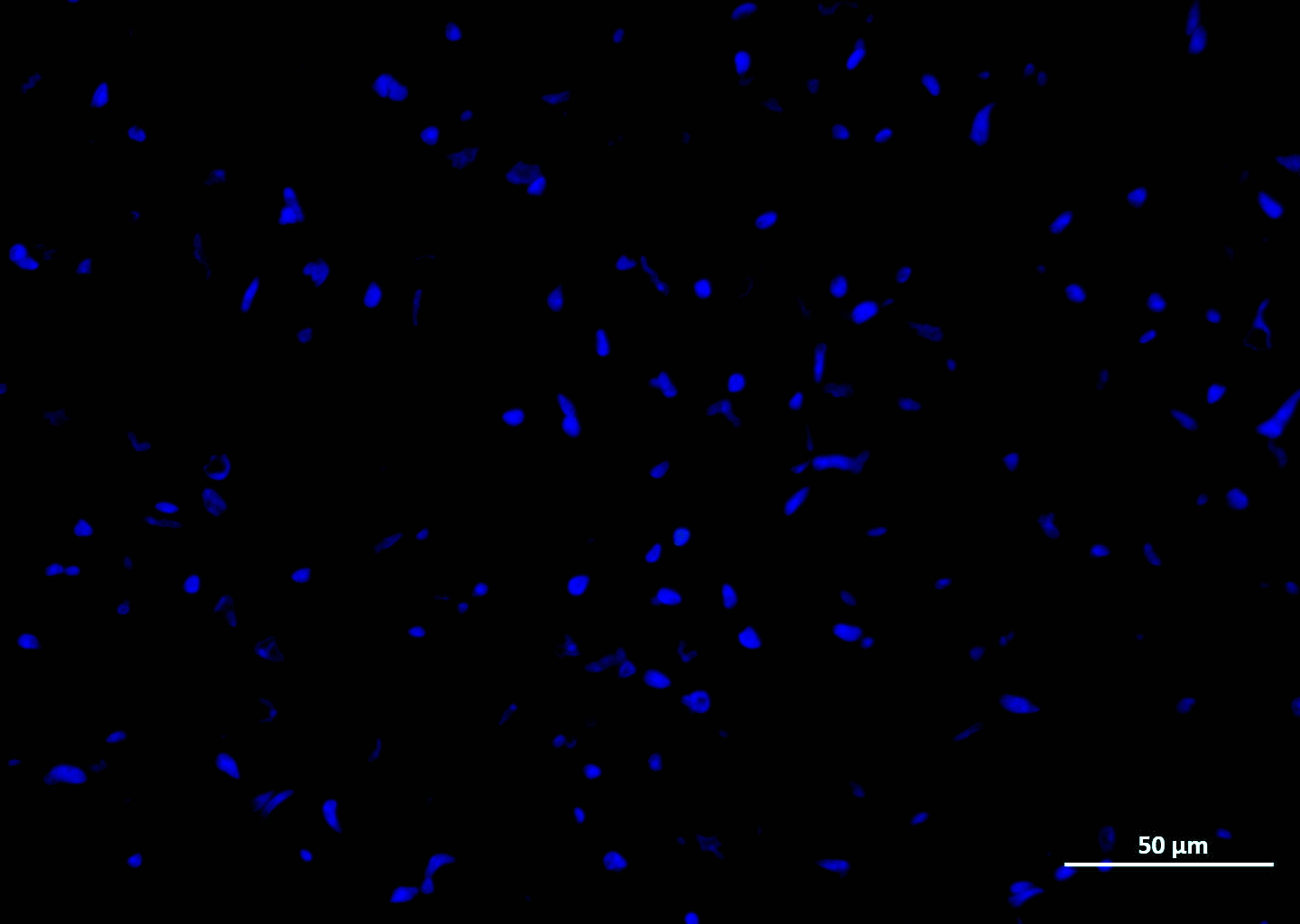

Supplement: Supplementary file 5 [file DataSheet6.zip › Tunel tissue/Control/1-1.jpg]

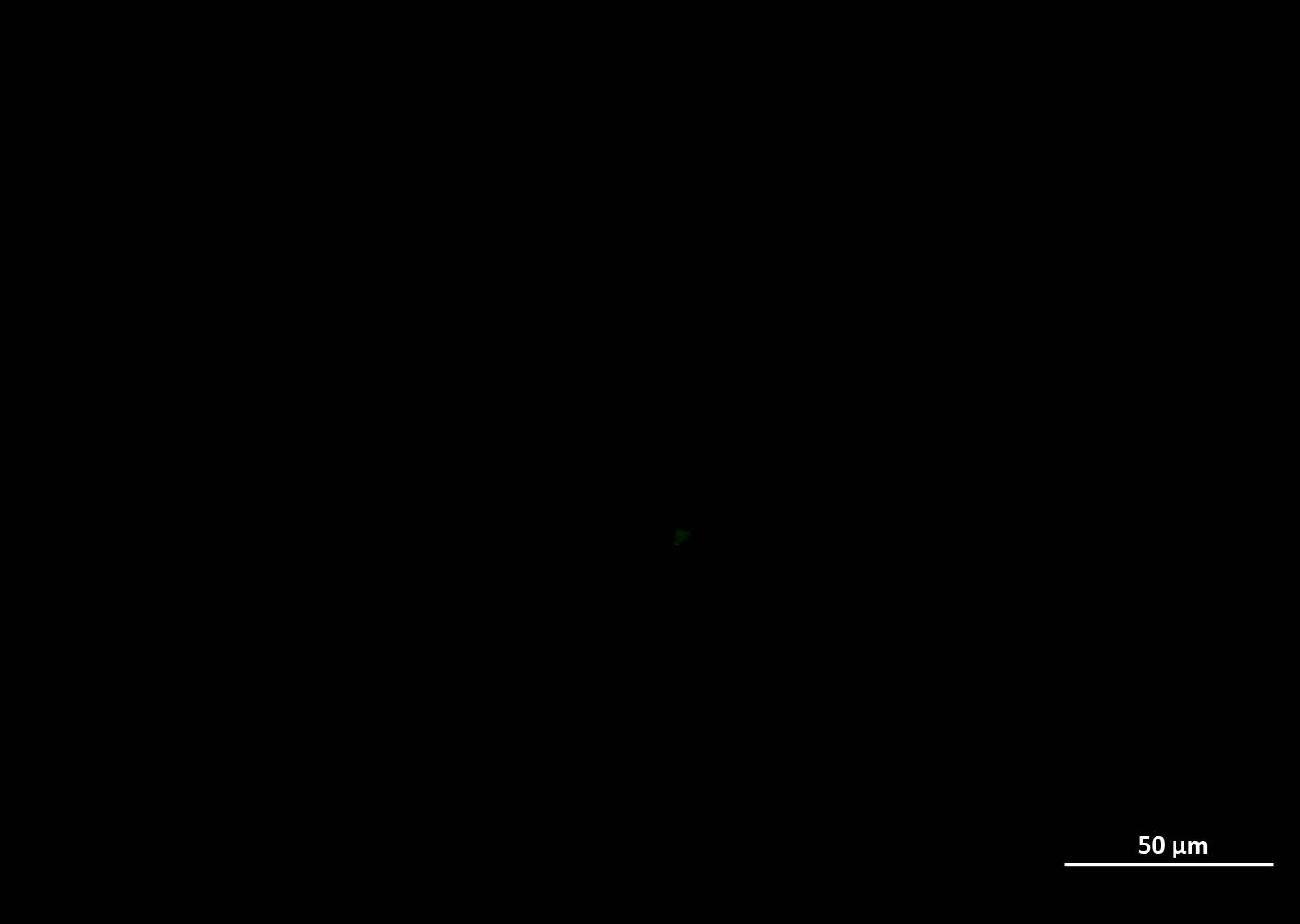

Supplement: Supplementary file 5 [file DataSheet6.zip › Tunel tissue/Control/1-2.jpg]

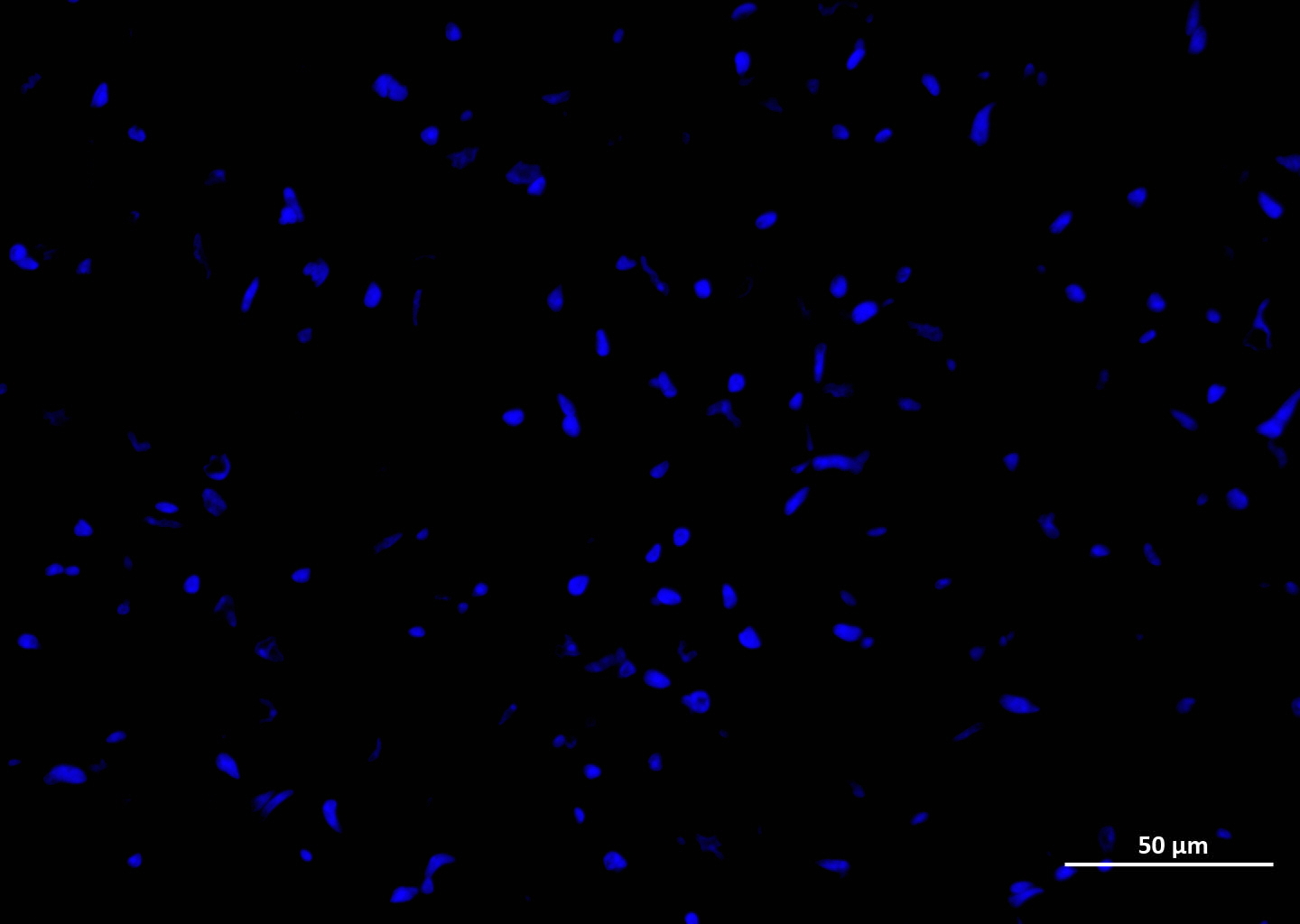

Supplement: Supplementary file 5 [file DataSheet6.zip › Tunel tissue/Control/1-3.jpg]

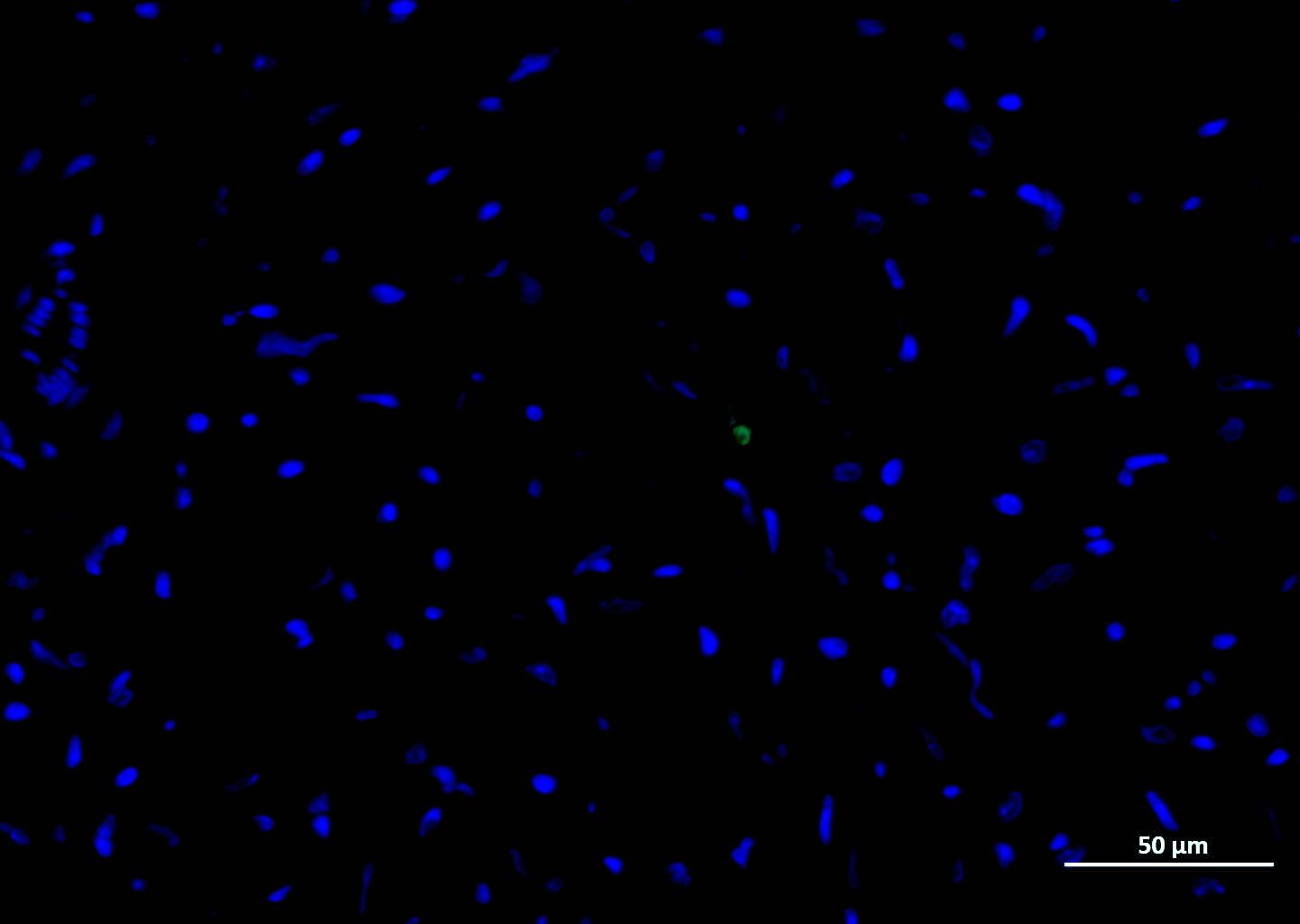

Supplement: Supplementary file 5 [file DataSheet6.zip › Tunel tissue/Control/2-1.jpg]

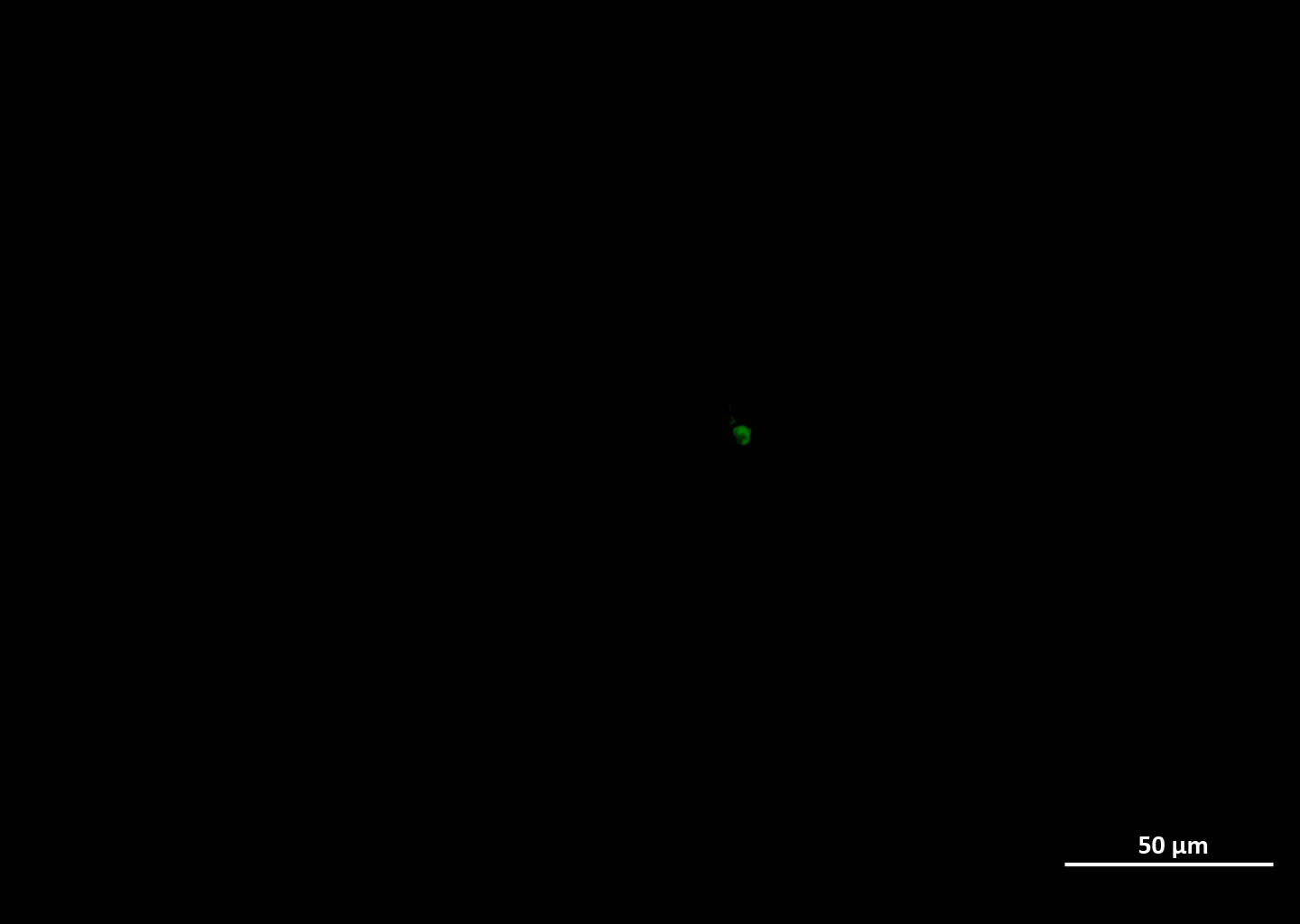

Supplement: Supplementary file 5 [file DataSheet6.zip › Tunel tissue/Control/2-2.jpg]

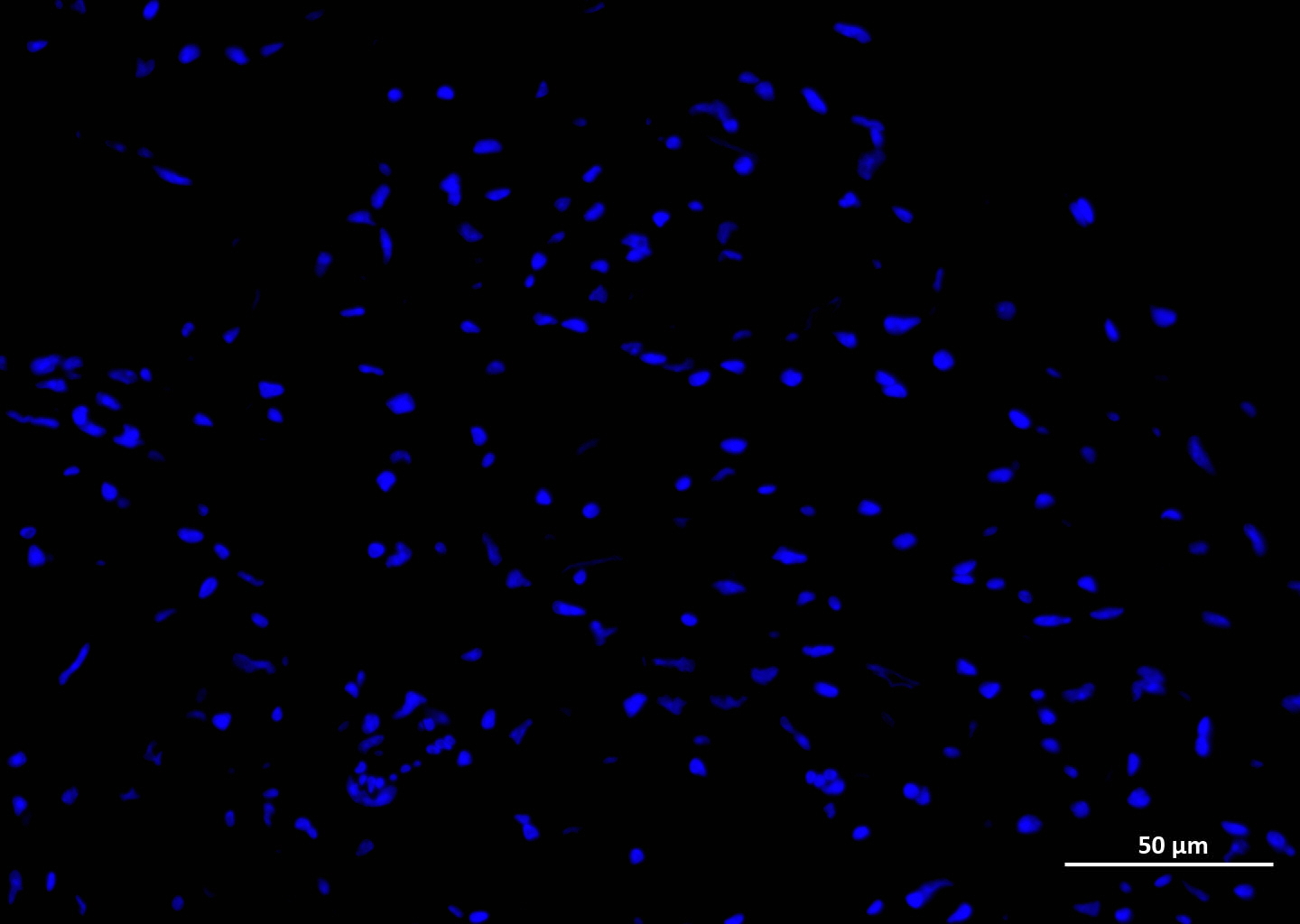

Supplement: Supplementary file 5 [file DataSheet6.zip › Tunel tissue/Control/2-3.jpg]

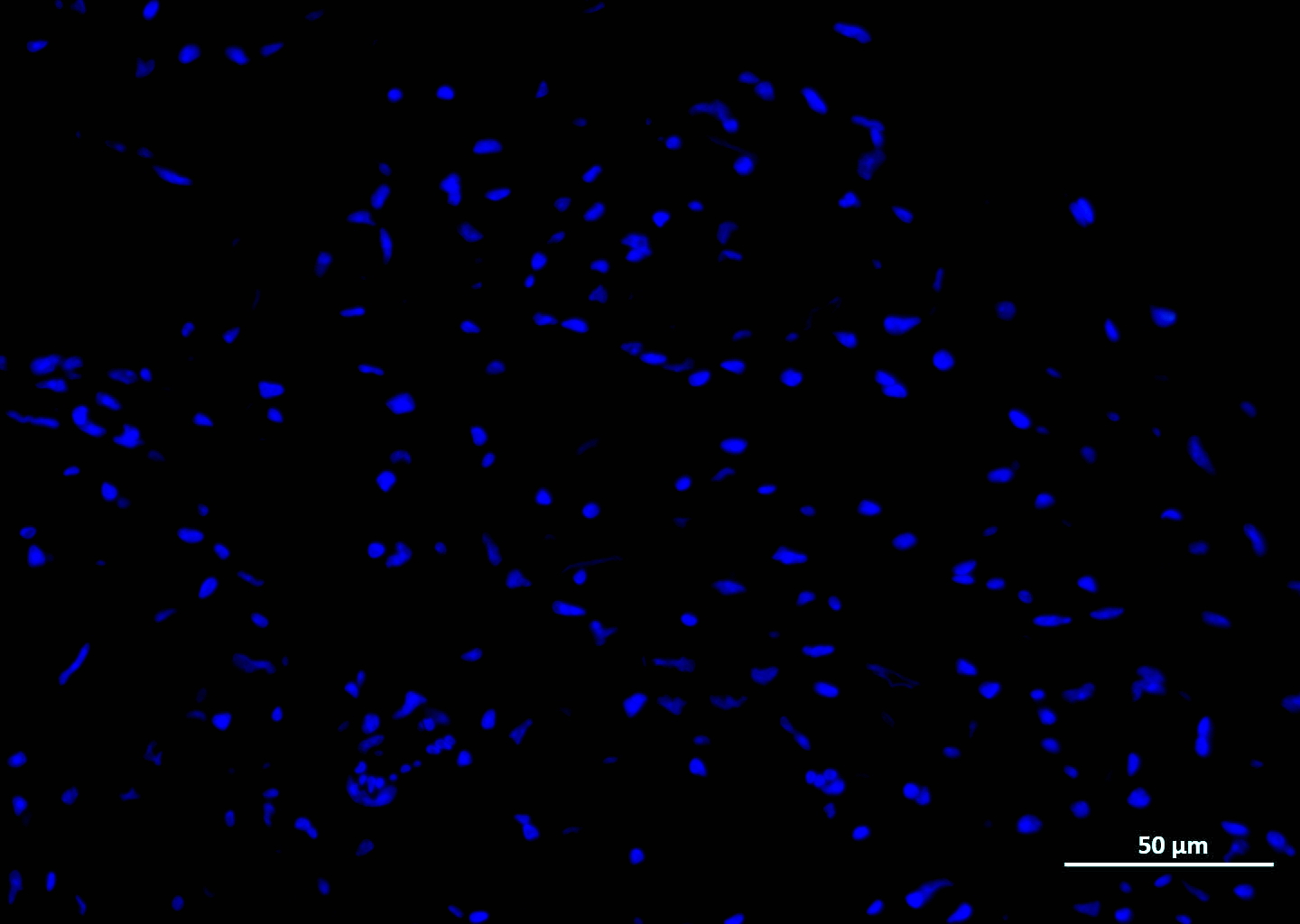

Supplement: Supplementary file 5 [file DataSheet6.zip › Tunel tissue/Control/3-1.jpg]

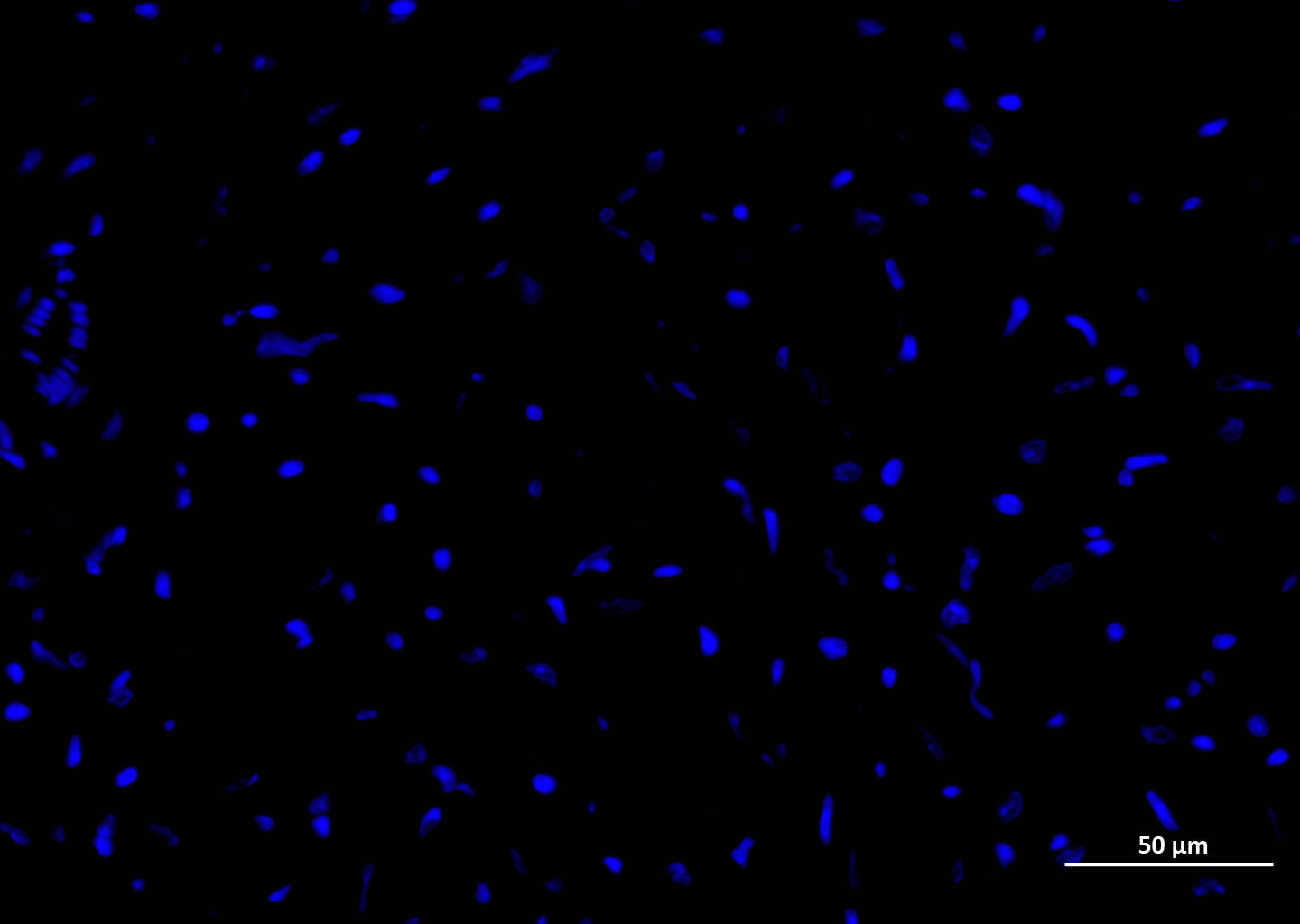

Supplement: Supplementary file 5 [file DataSheet6.zip › Tunel tissue/Control/3-2.jpg]

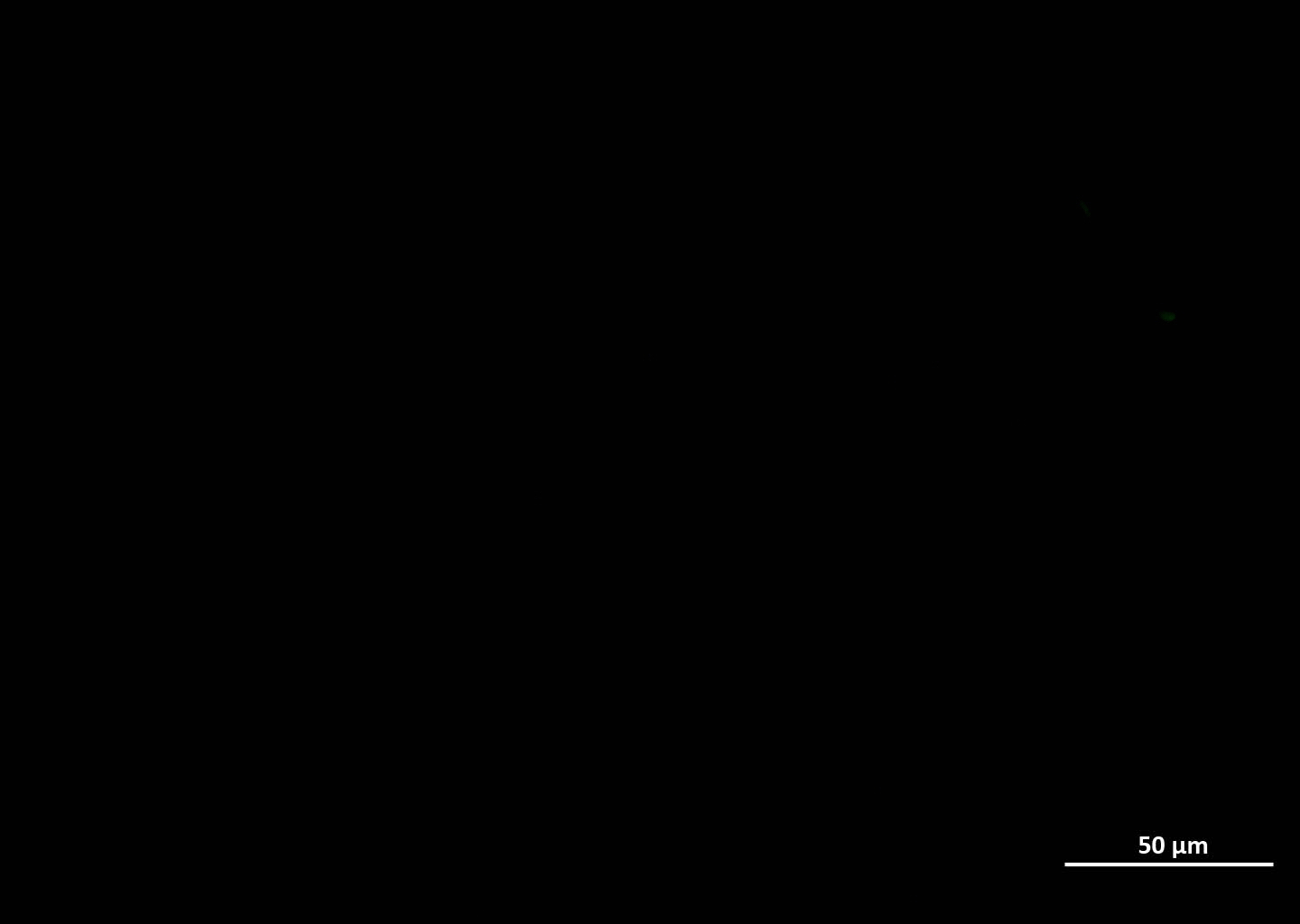

Supplement: Supplementary file 5 [file DataSheet6.zip › Tunel tissue/Control/3-3.jpg]

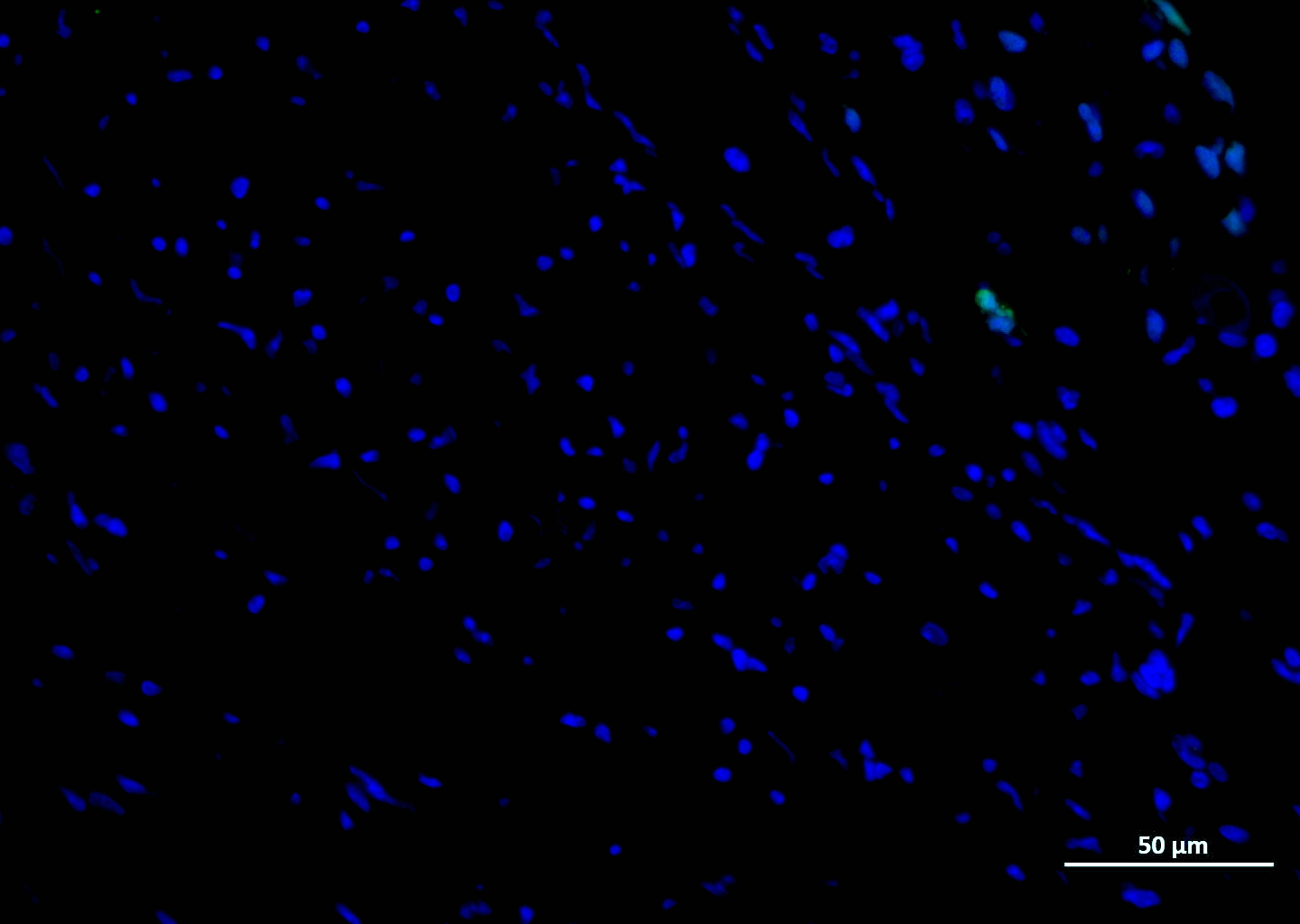

Supplement: Supplementary file 5 [file DataSheet6.zip › Tunel tissue/ISO/7-1.jpg]

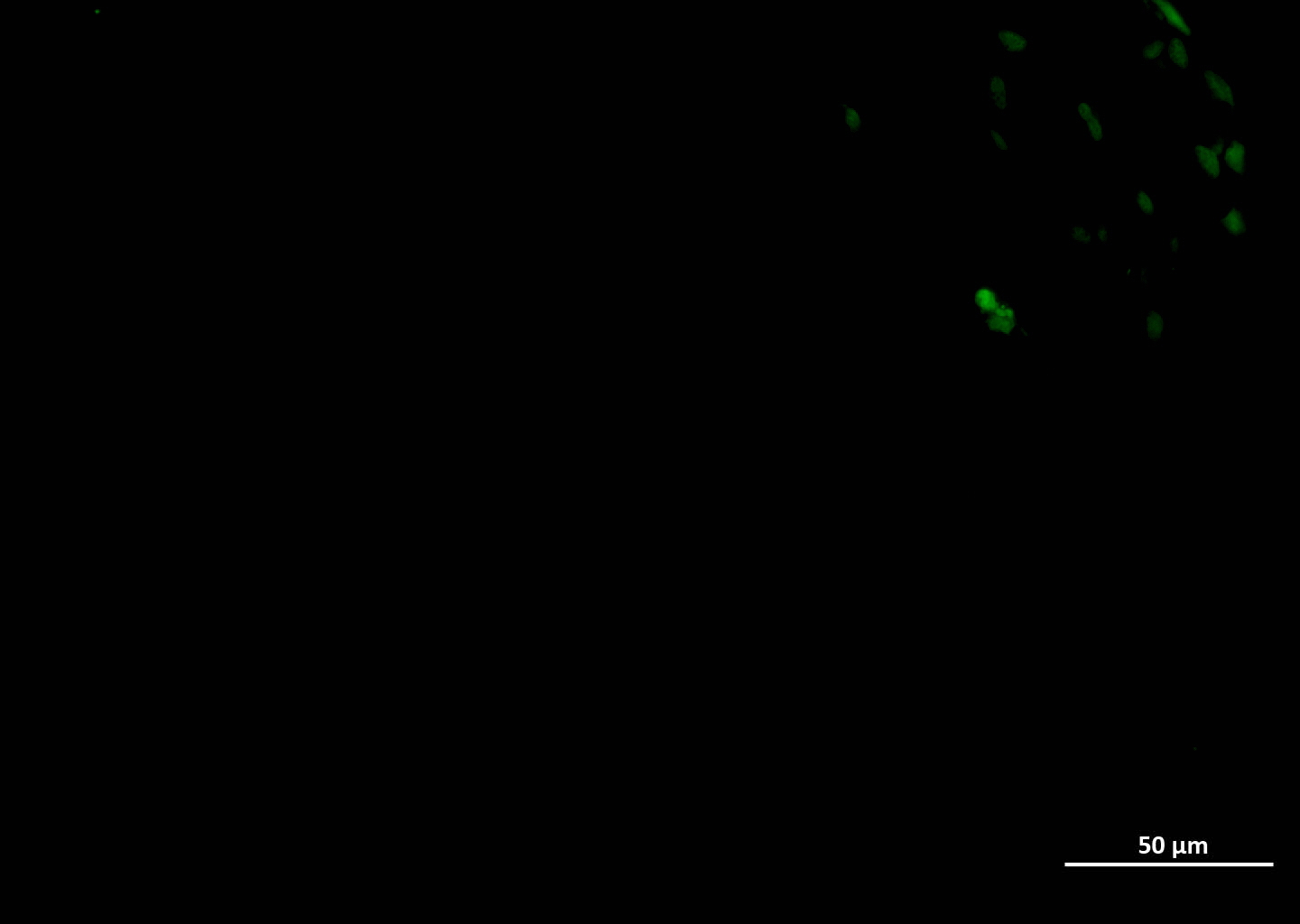

Supplement: Supplementary file 5 [file DataSheet6.zip › Tunel tissue/ISO/7-2.jpg]

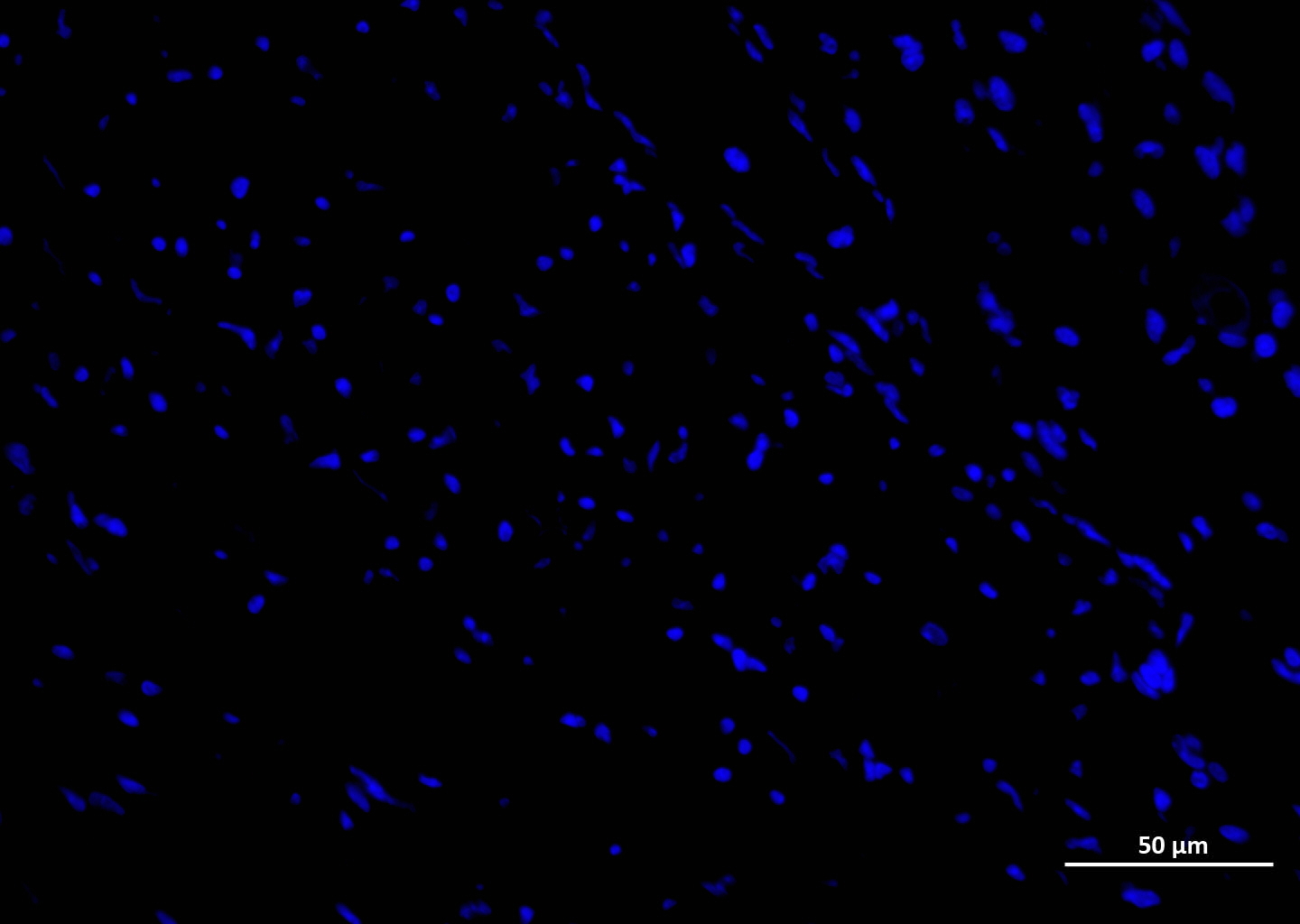

Supplement: Supplementary file 5 [file DataSheet6.zip › Tunel tissue/ISO/7-3.jpg]

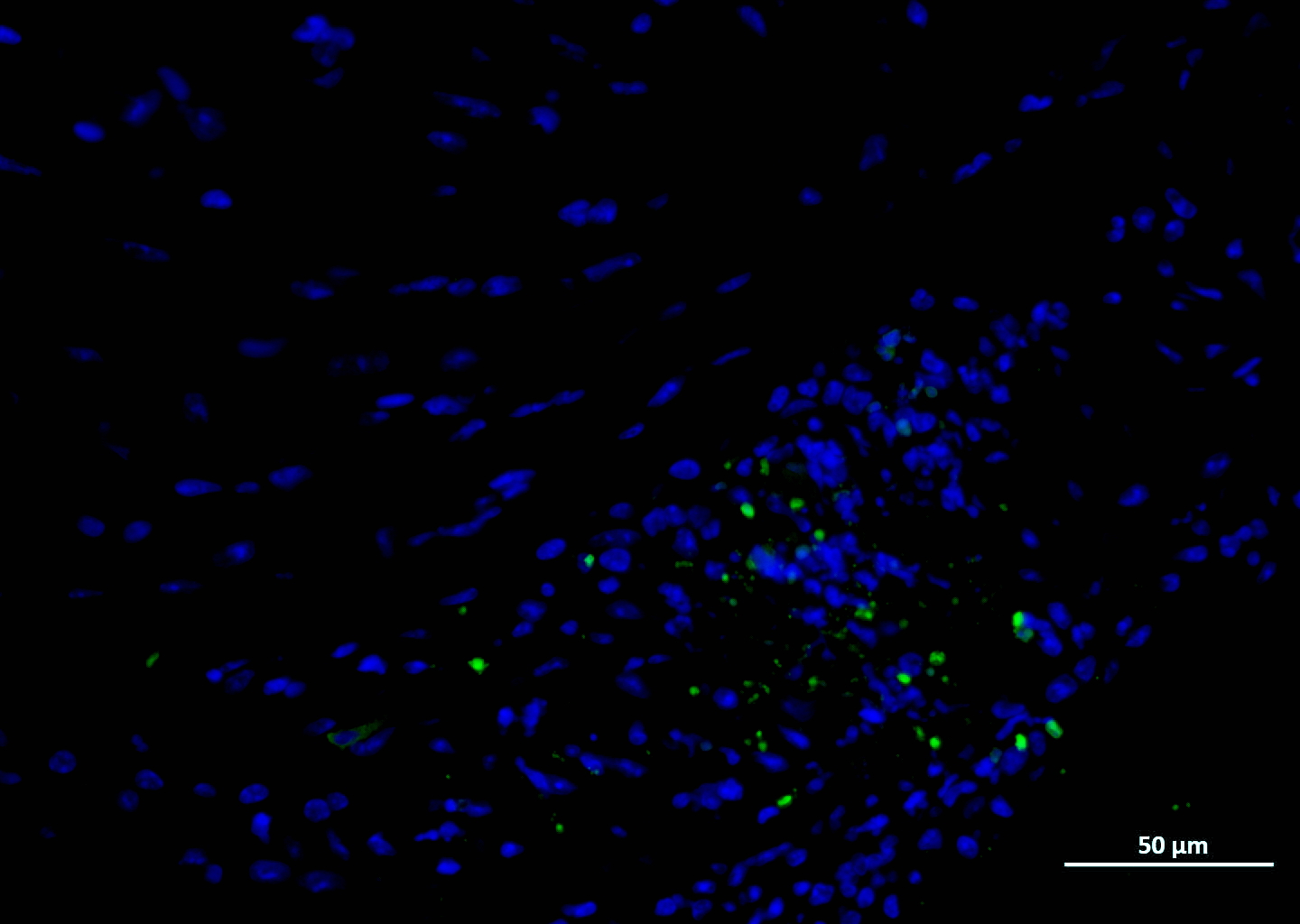

Supplement: Supplementary file 5 [file DataSheet6.zip › Tunel tissue/ISO/8-1.jpg]

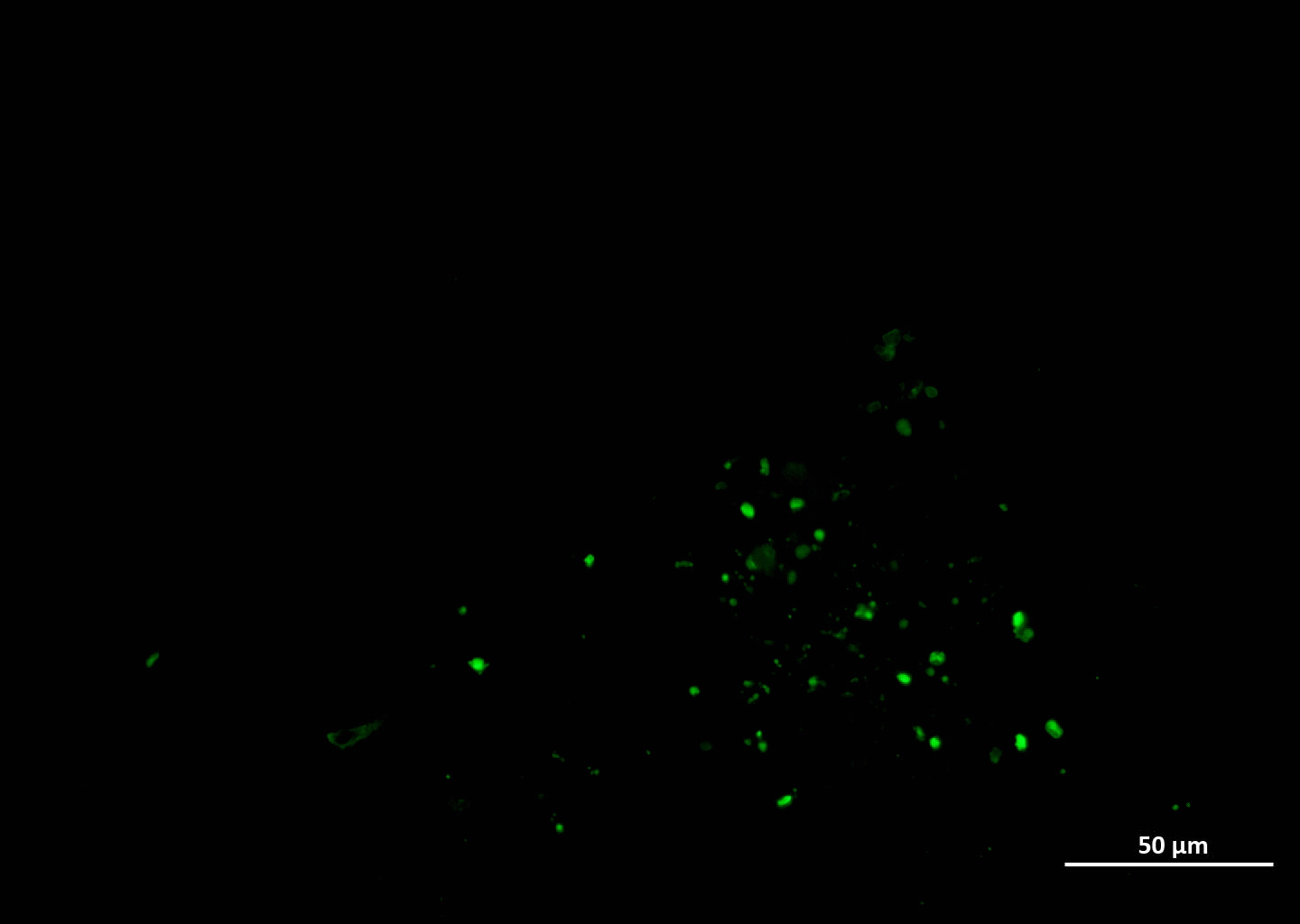

Supplement: Supplementary file 5 [file DataSheet6.zip › Tunel tissue/ISO/8-2.jpg]

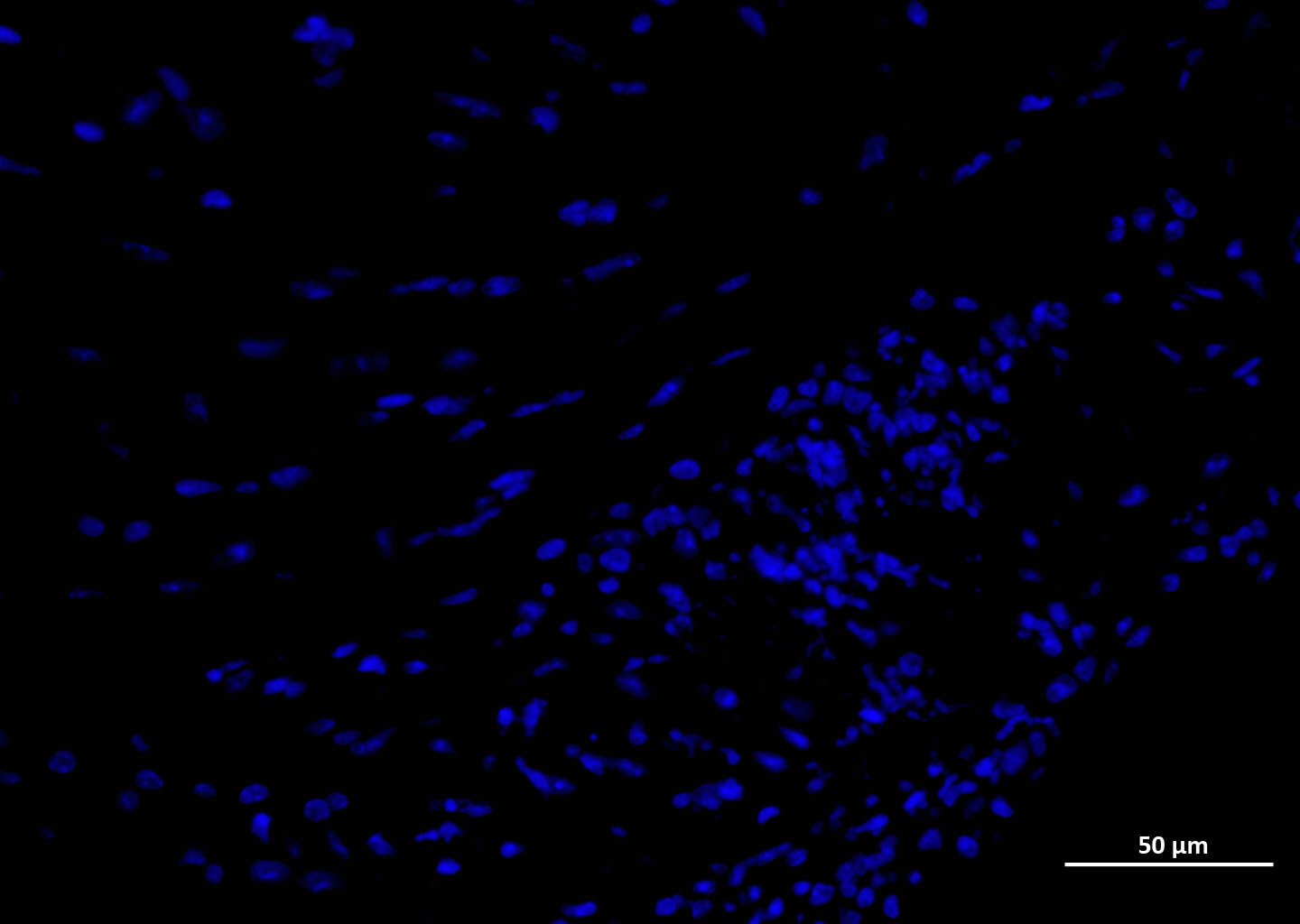

Supplement: Supplementary file 5 [file DataSheet6.zip › Tunel tissue/ISO/8-3.jpg]

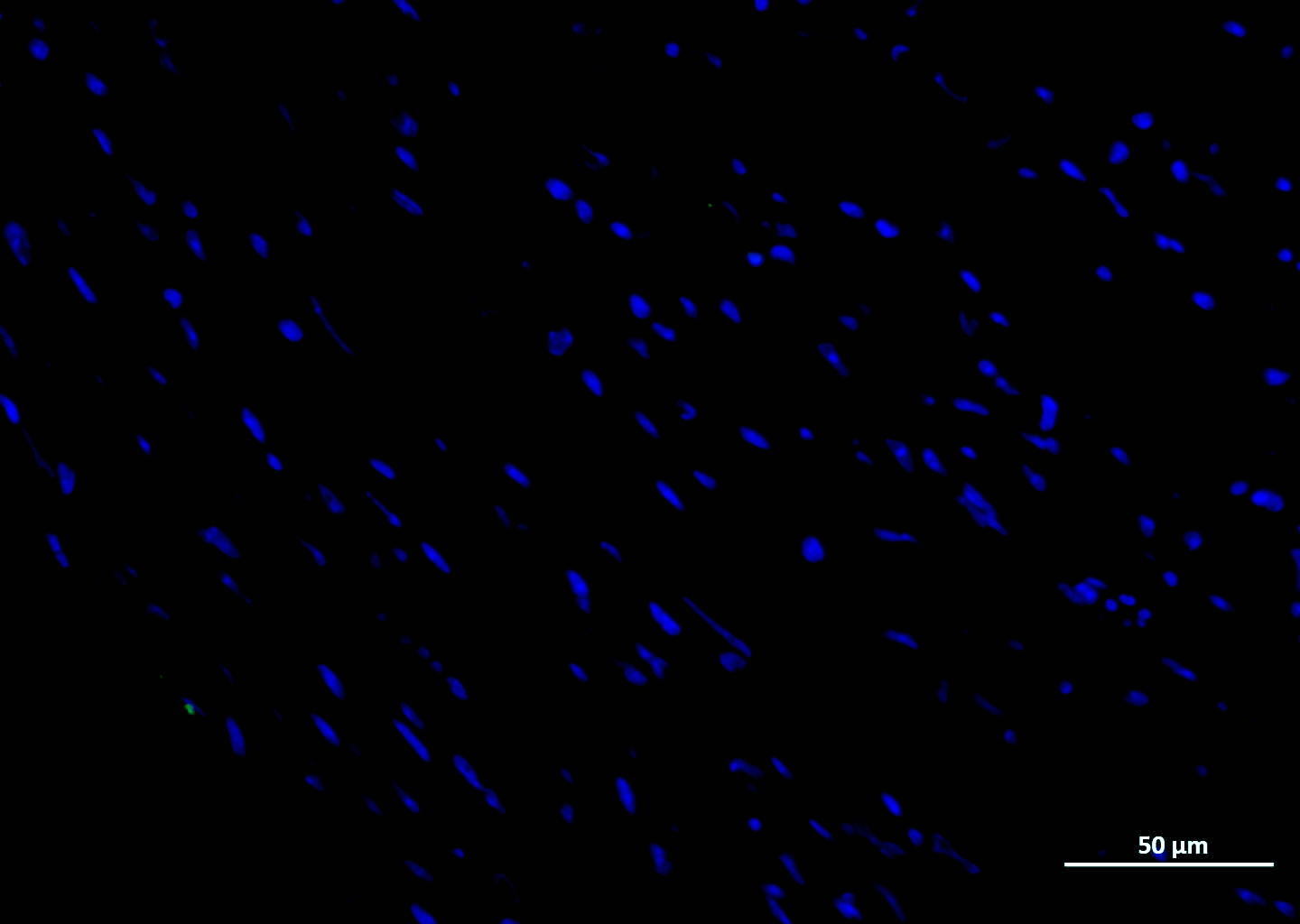

Supplement: Supplementary file 5 [file DataSheet6.zip › Tunel tissue/ISO + IDE 1/4-1.jpg]

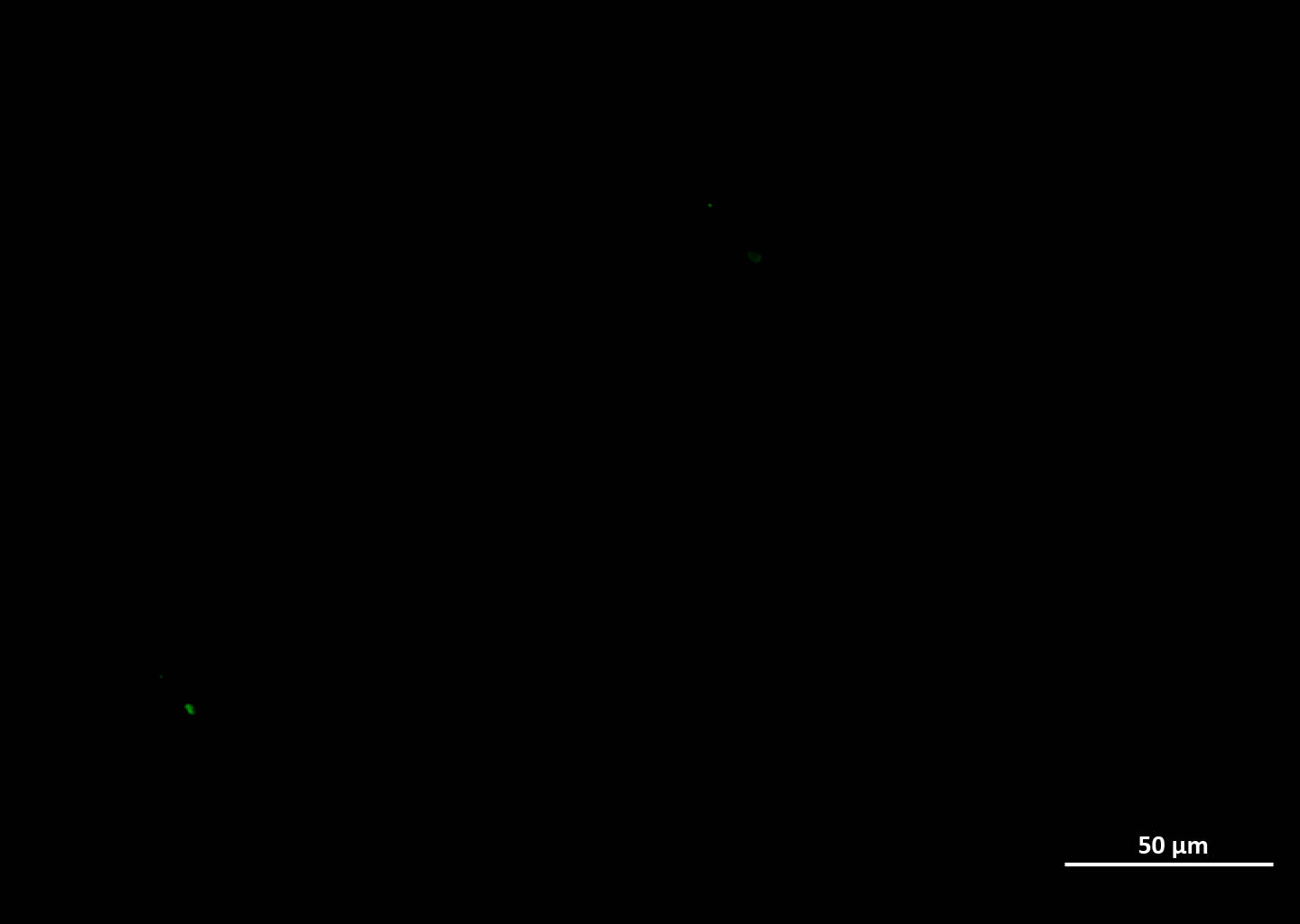

Supplement: Supplementary file 5 [file DataSheet6.zip › Tunel tissue/ISO + IDE 1/4-2.jpg]

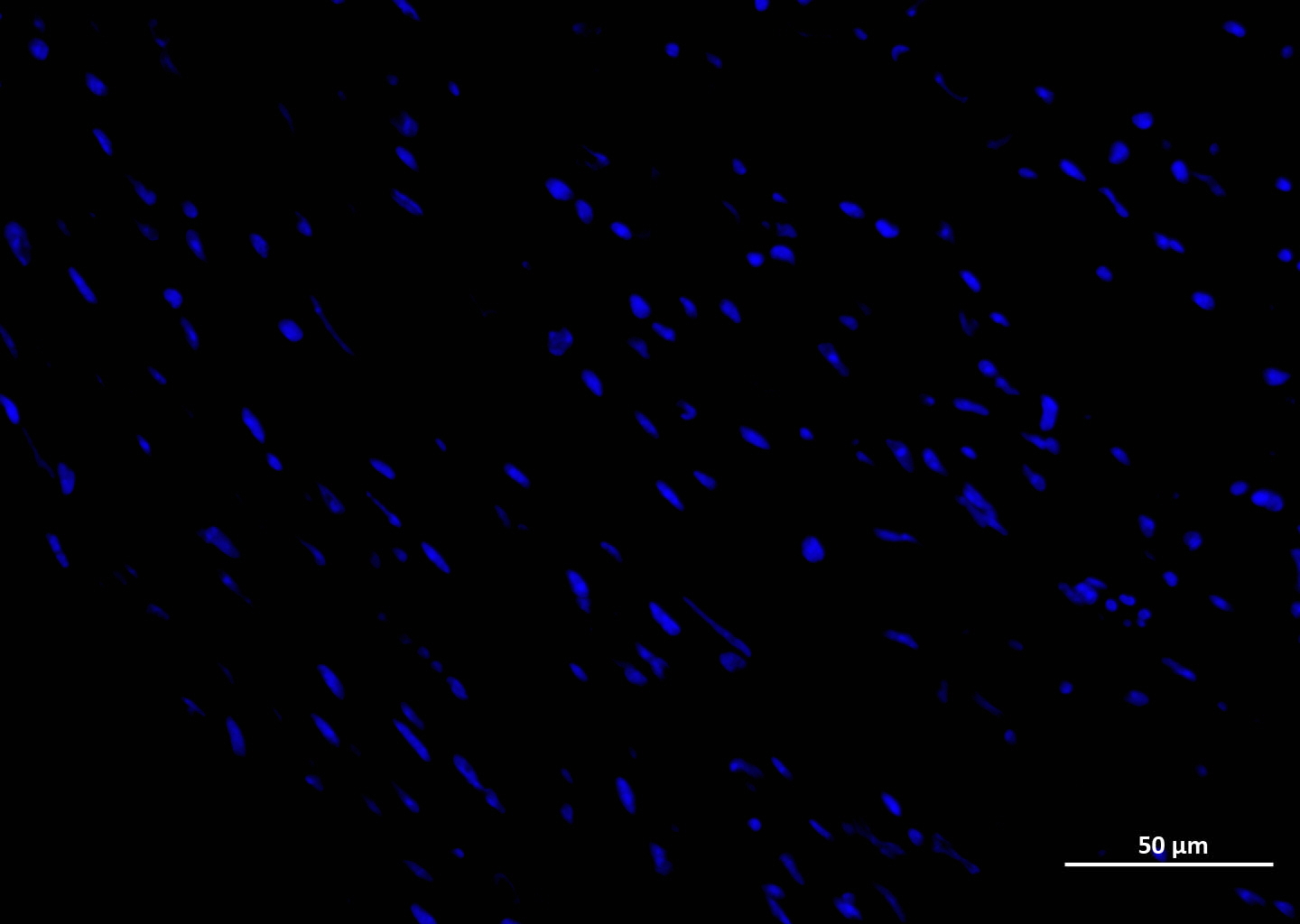

Supplement: Supplementary file 5 [file DataSheet6.zip › Tunel tissue/ISO + IDE 1/4-3.jpg]

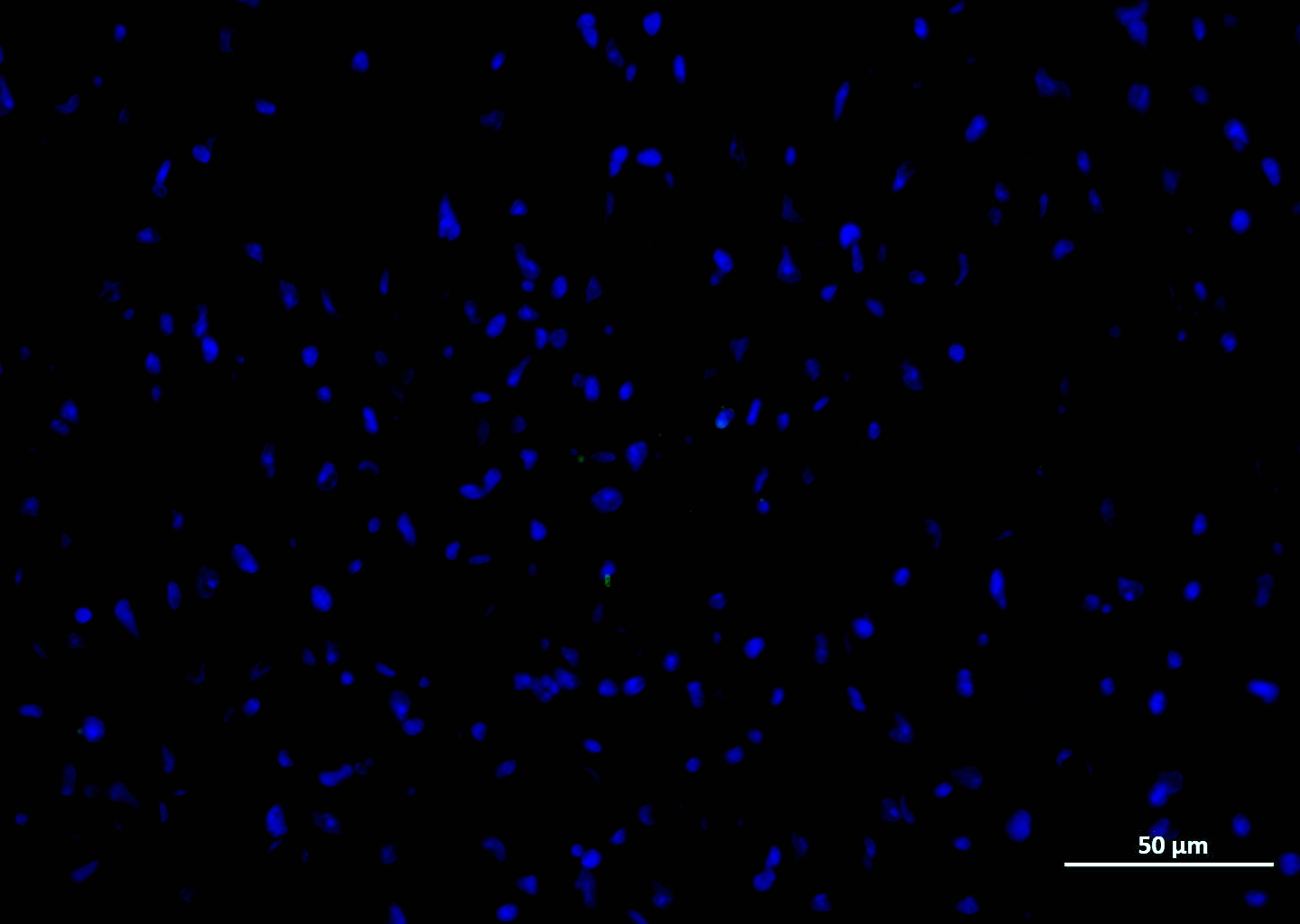

Supplement: Supplementary file 5 [file DataSheet6.zip › Tunel tissue/ISO + IDE 1/5-1.jpg]

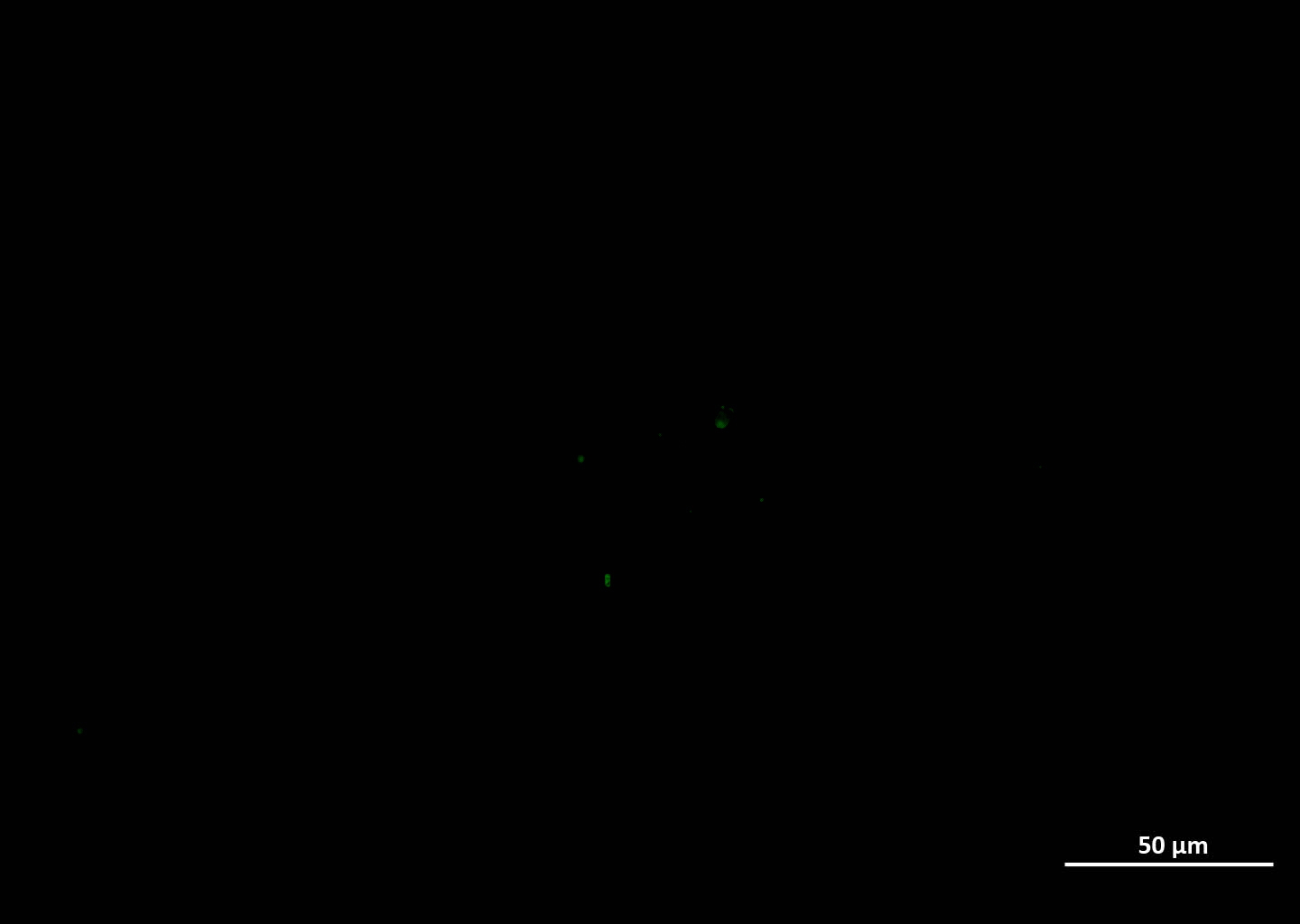

Supplement: Supplementary file 5 [file DataSheet6.zip › Tunel tissue/ISO + IDE 1/5-2.jpg]

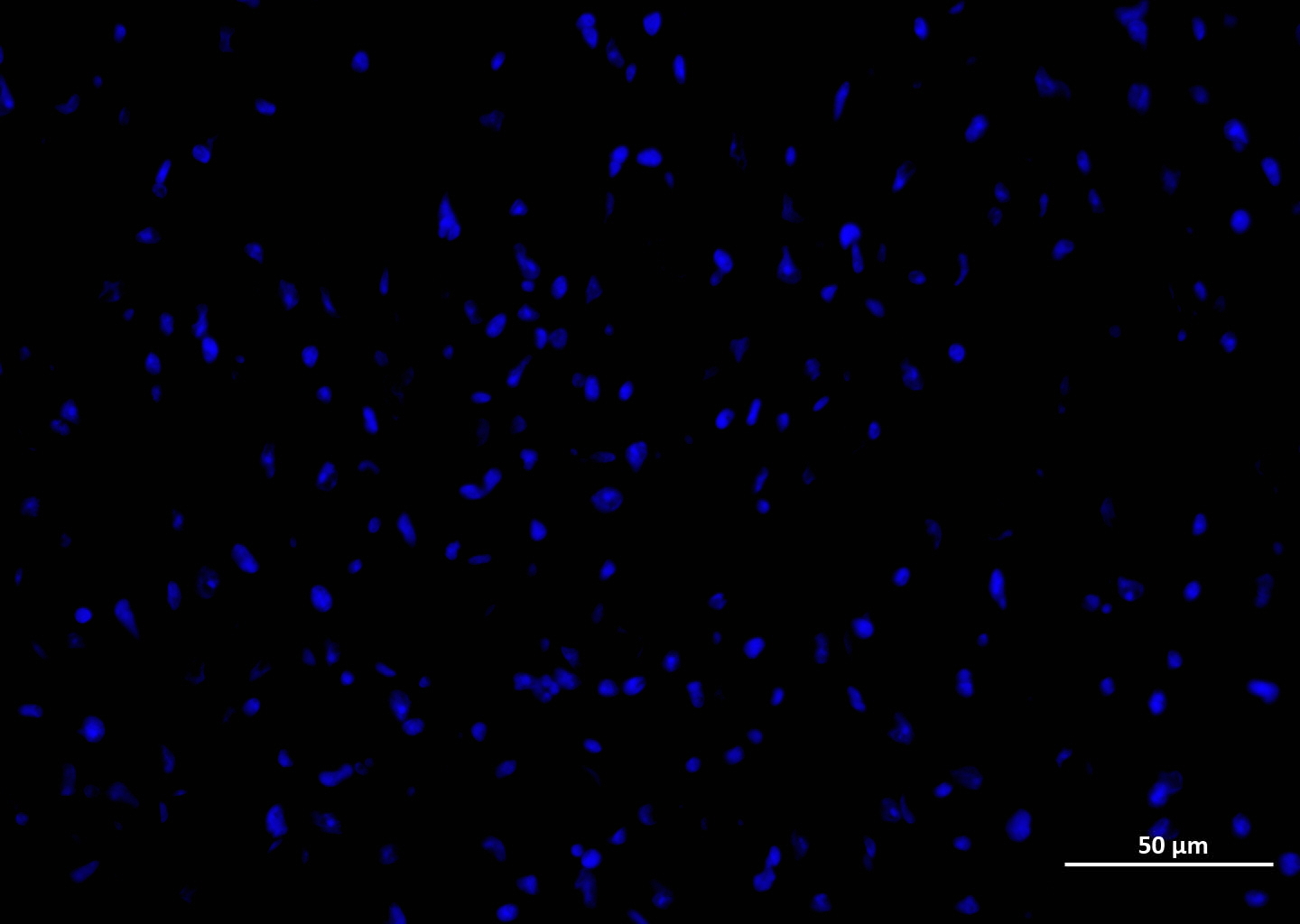

Supplement: Supplementary file 5 [file DataSheet6.zip › Tunel tissue/ISO + IDE 1/5-3.jpg]

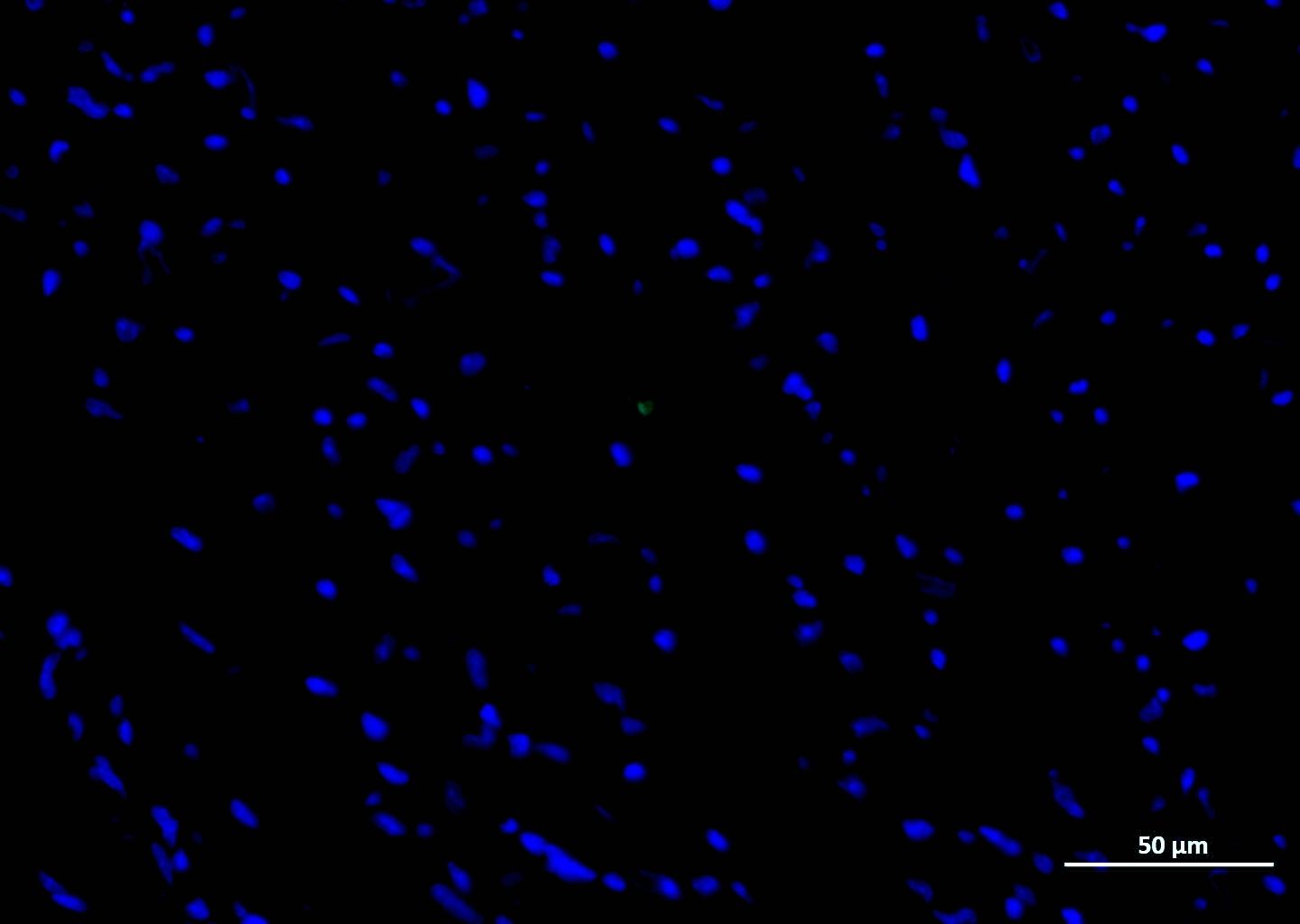

Supplement: Supplementary file 5 [file DataSheet6.zip › Tunel tissue/ISO + IDE 1/6-1.jpg]

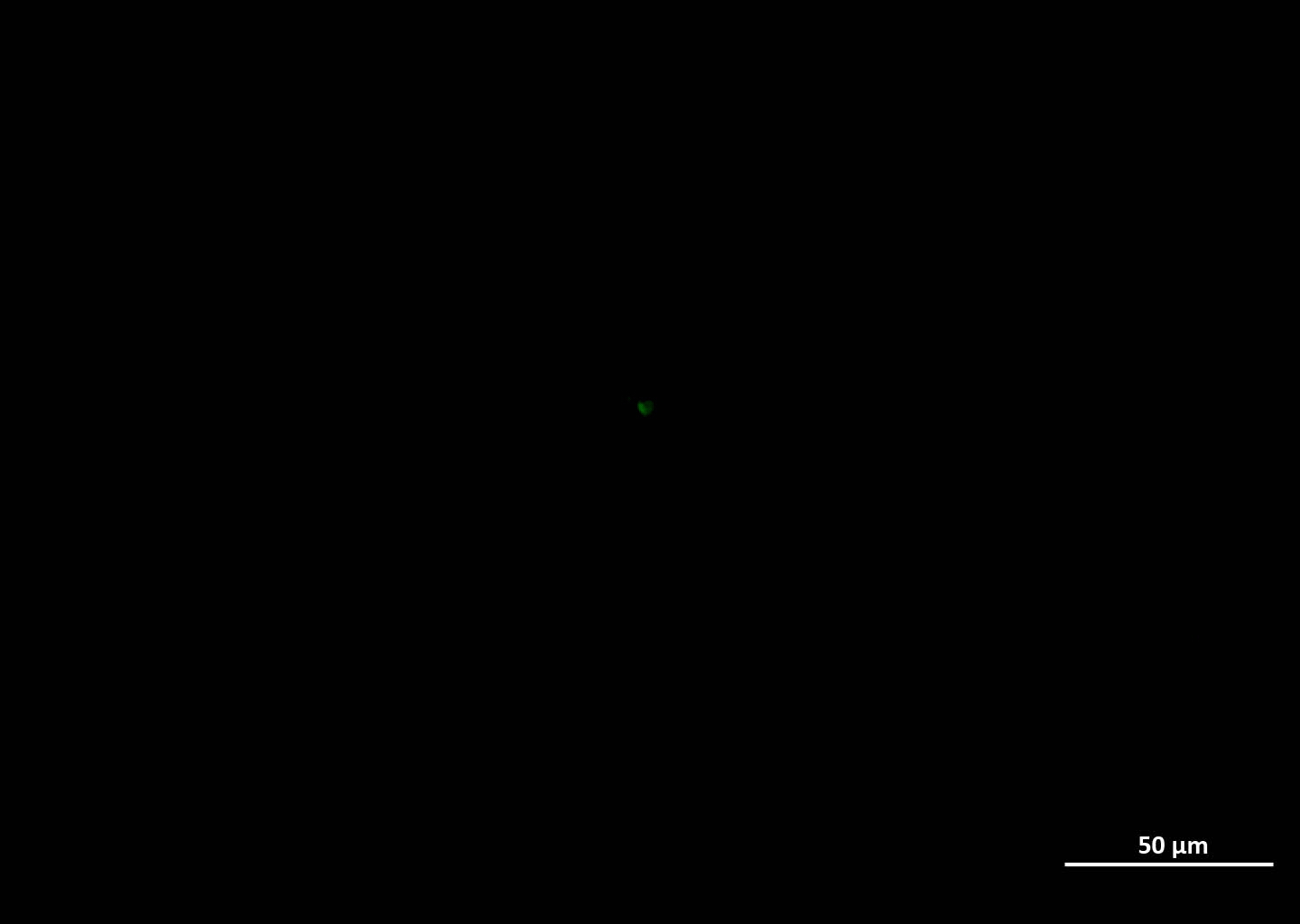

Supplement: Supplementary file 5 [file DataSheet6.zip › Tunel tissue/ISO + IDE 1/6-2.jpg]

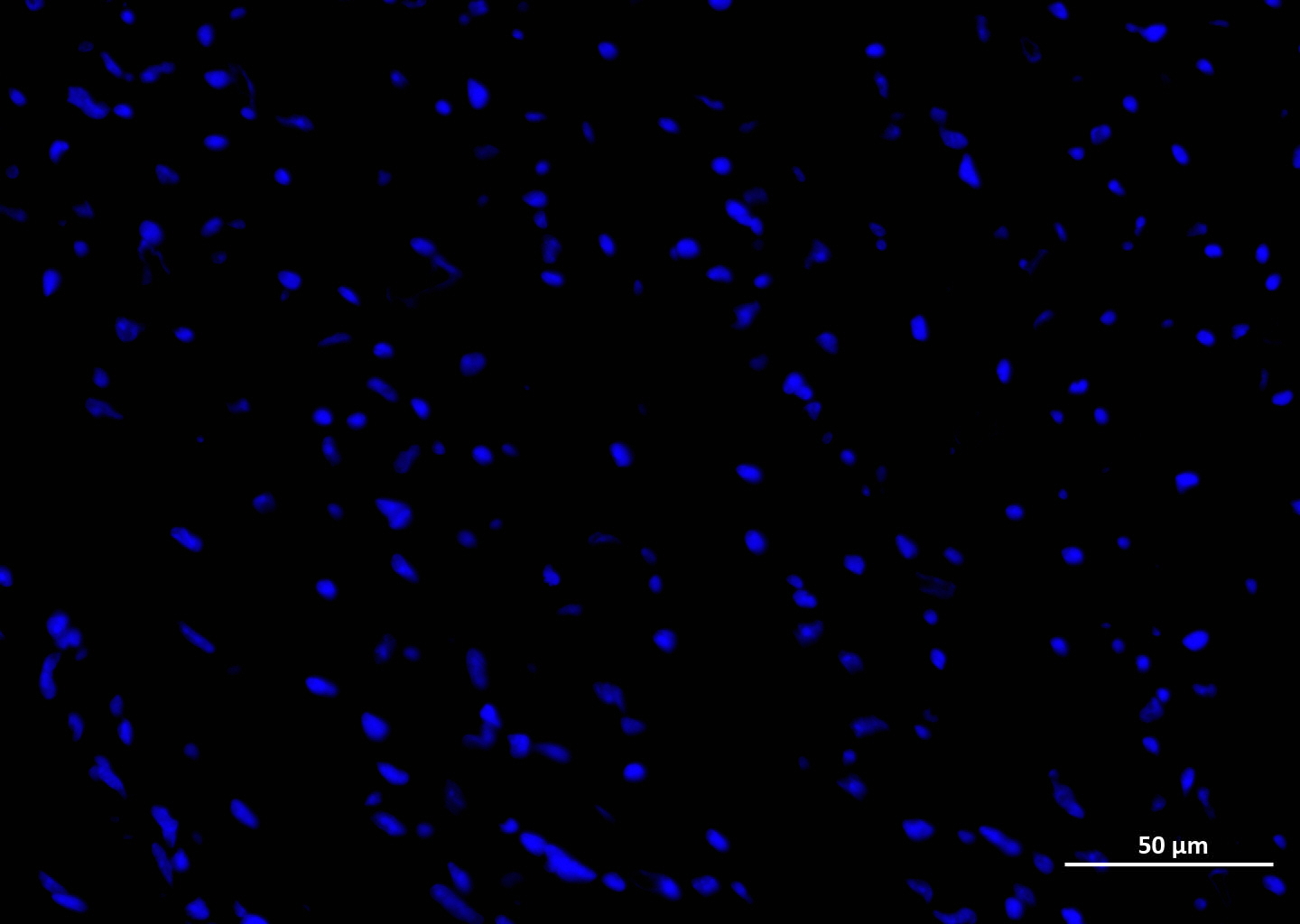

Supplement: Supplementary file 5 [file DataSheet6.zip › Tunel tissue/ISO + IDE 1/6-3.jpg]

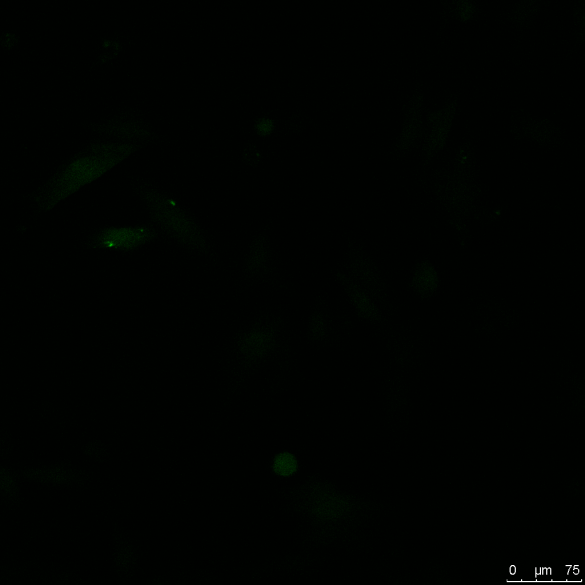

Supplement: Supplementary file 6 [file DataSheet2.zip › ROS Original data/control(1).tif]

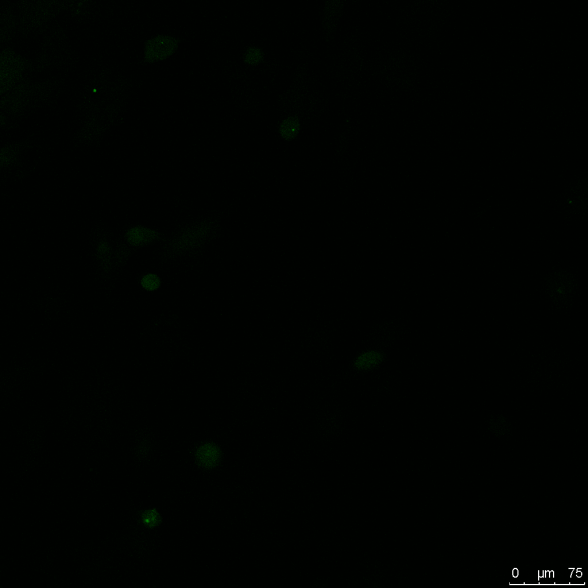

Supplement: Supplementary file 6 [file DataSheet2.zip › ROS Original data/control(2).tif]

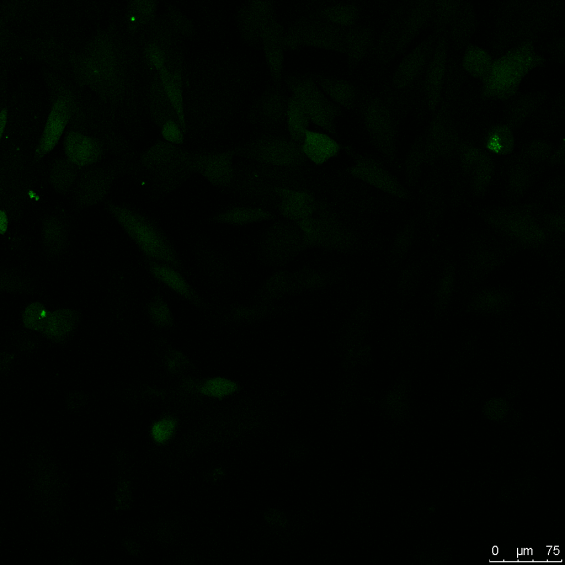

Supplement: Supplementary file 6 [file DataSheet2.zip › ROS Original data/control(3).tif]

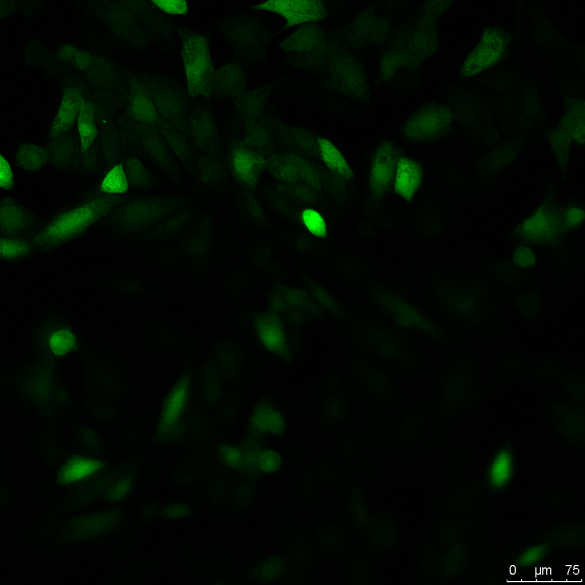

Supplement: Supplementary file 6 [file DataSheet2.zip › ROS Original data/H2O2 (1).tif]

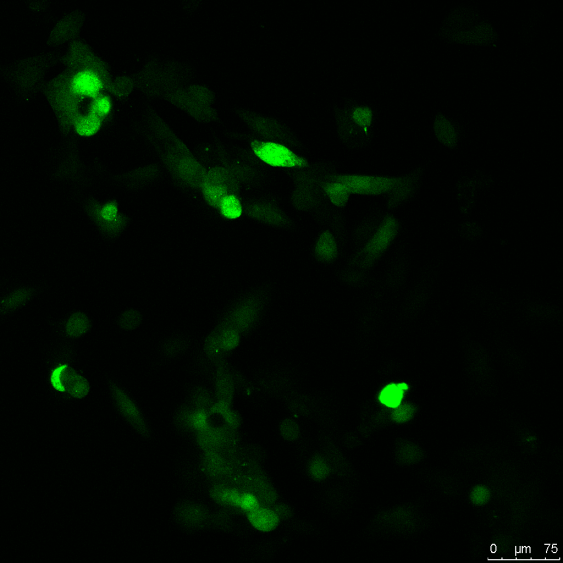

Supplement: Supplementary file 6 [file DataSheet2.zip › ROS Original data/H2O2 (2).tif]

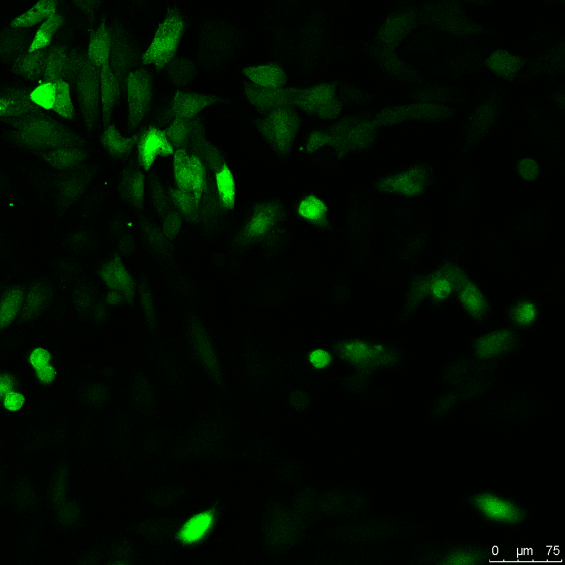

Supplement: Supplementary file 6 [file DataSheet2.zip › ROS Original data/H2O2 (3).tif]

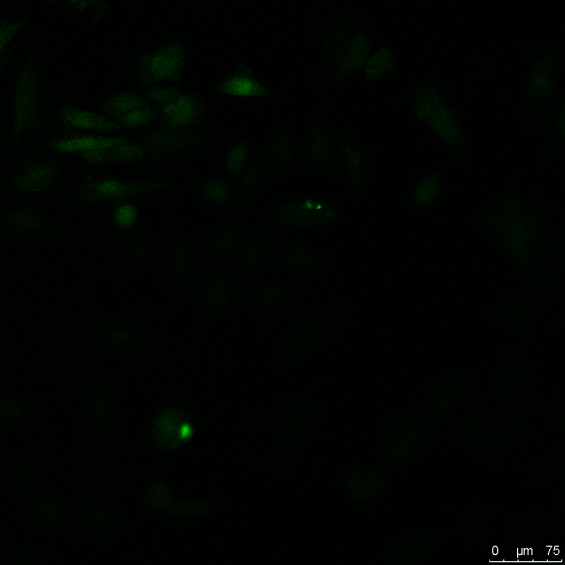

Supplement: Supplementary file 6 [file DataSheet2.zip › ROS Original data/H2O2+IDE-1(1).tif]

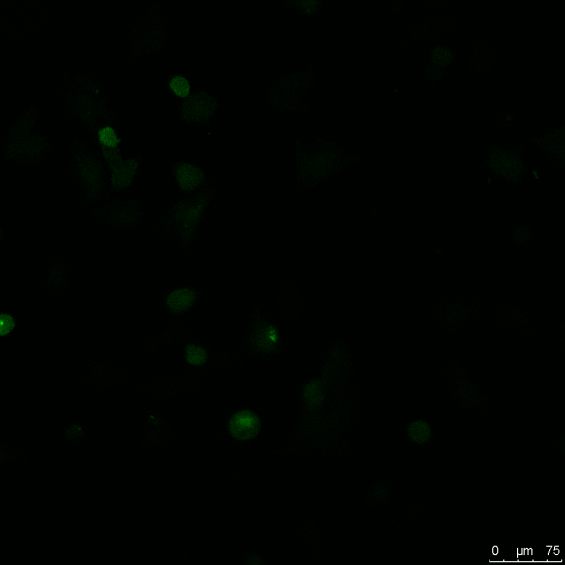

Supplement: Supplementary file 6 [file DataSheet2.zip › ROS Original data/H2O2+IDE-1(2).tif]

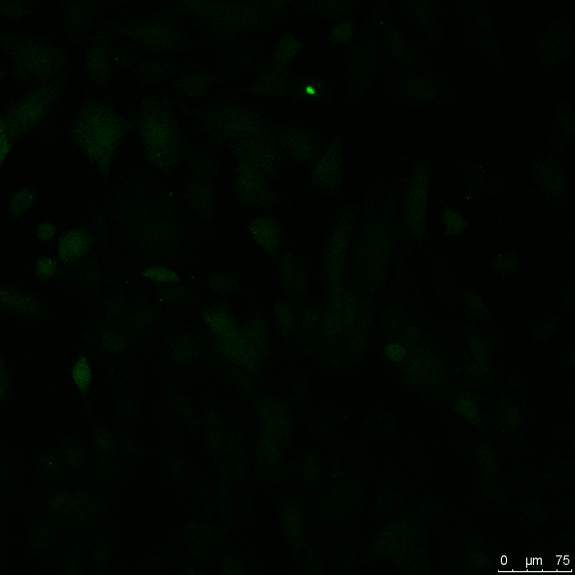

Supplement: Supplementary file 6 [file DataSheet2.zip › ROS Original data/H2O2+IDE-1(3).tif]

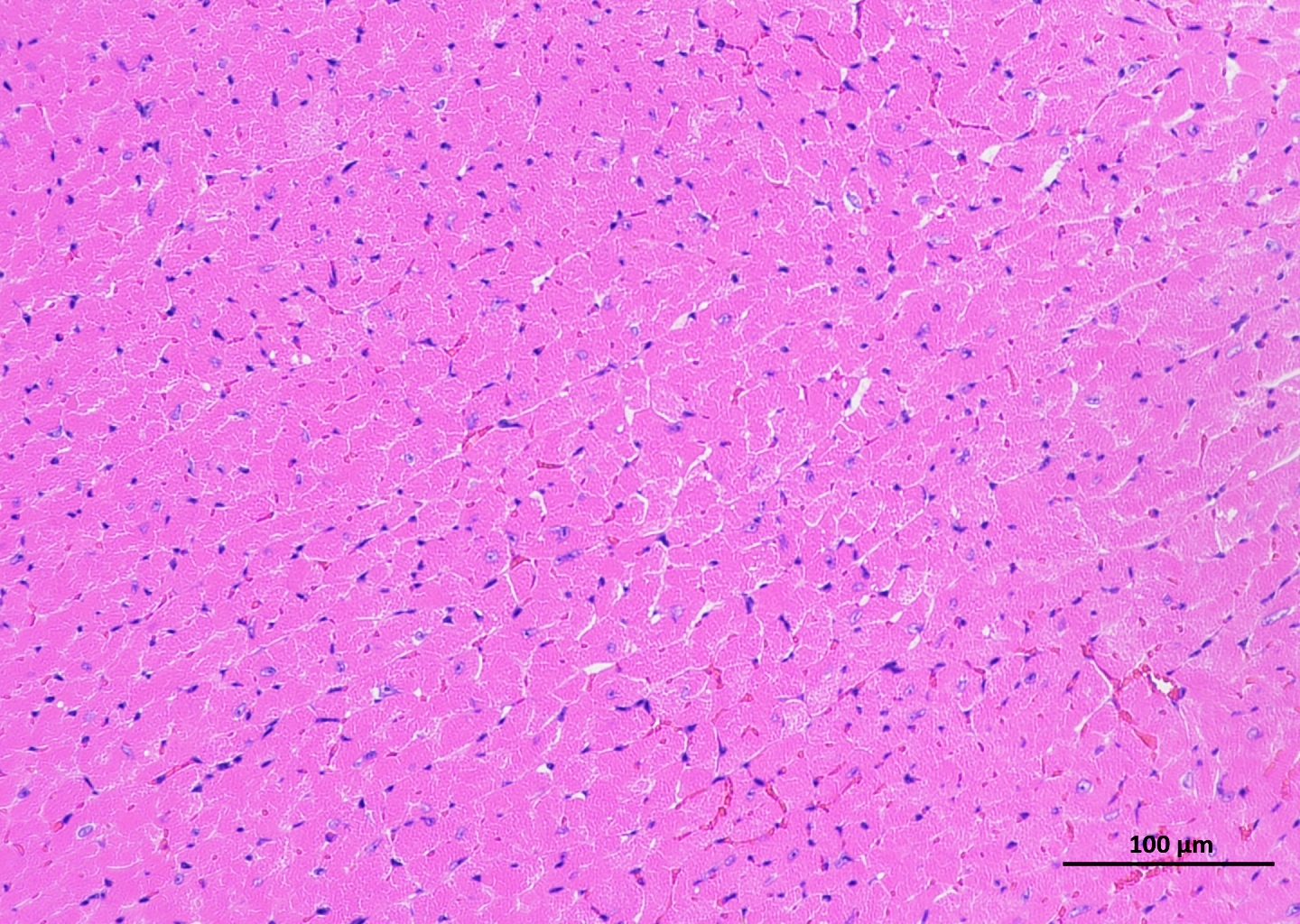

Supplement: Supplementary file 7 [file DataSheet5.zip › HE tissue/control.tif]

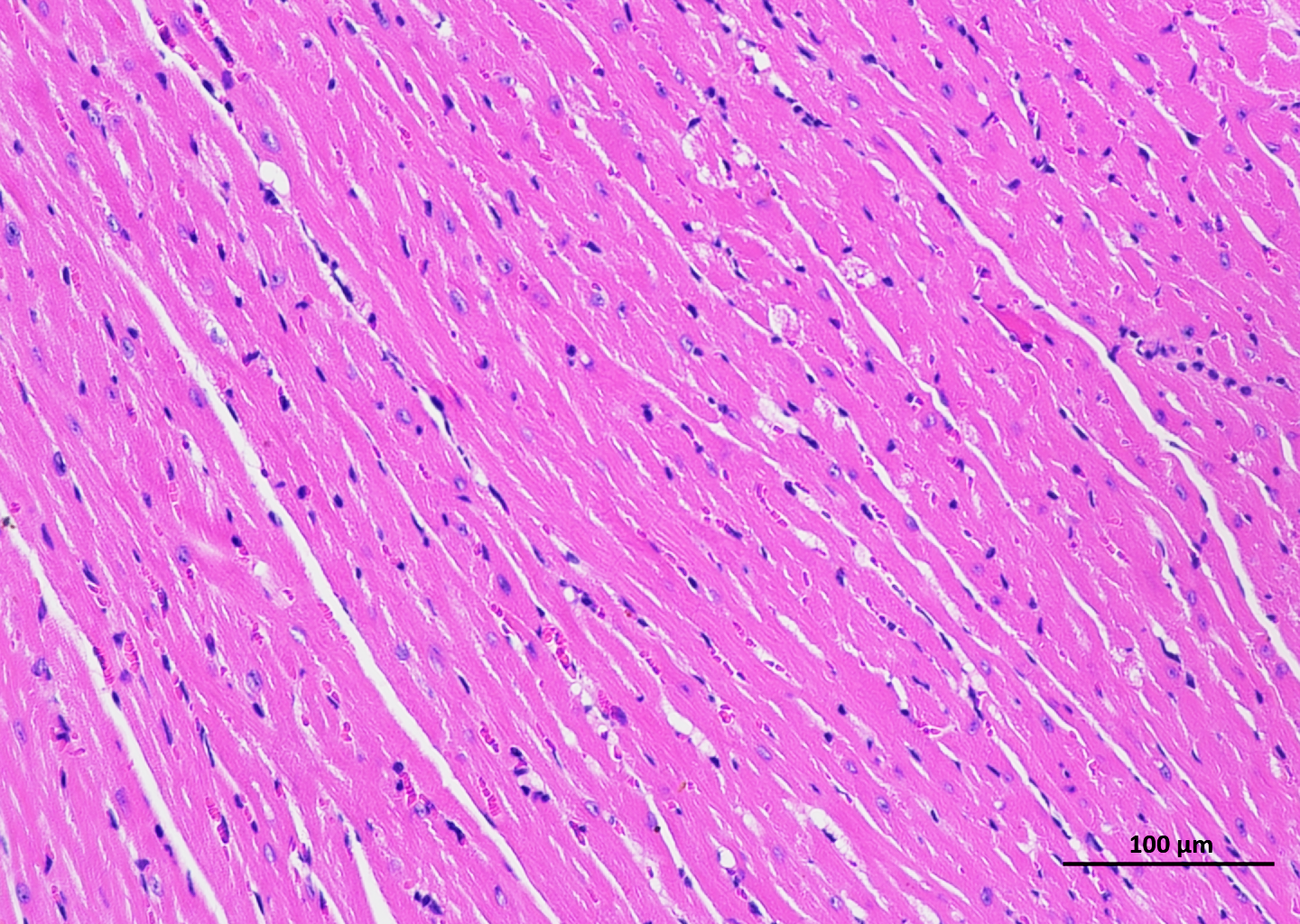

Supplement: Supplementary file 7 [file DataSheet5.zip › HE tissue/ISO + IDE-1.tif]

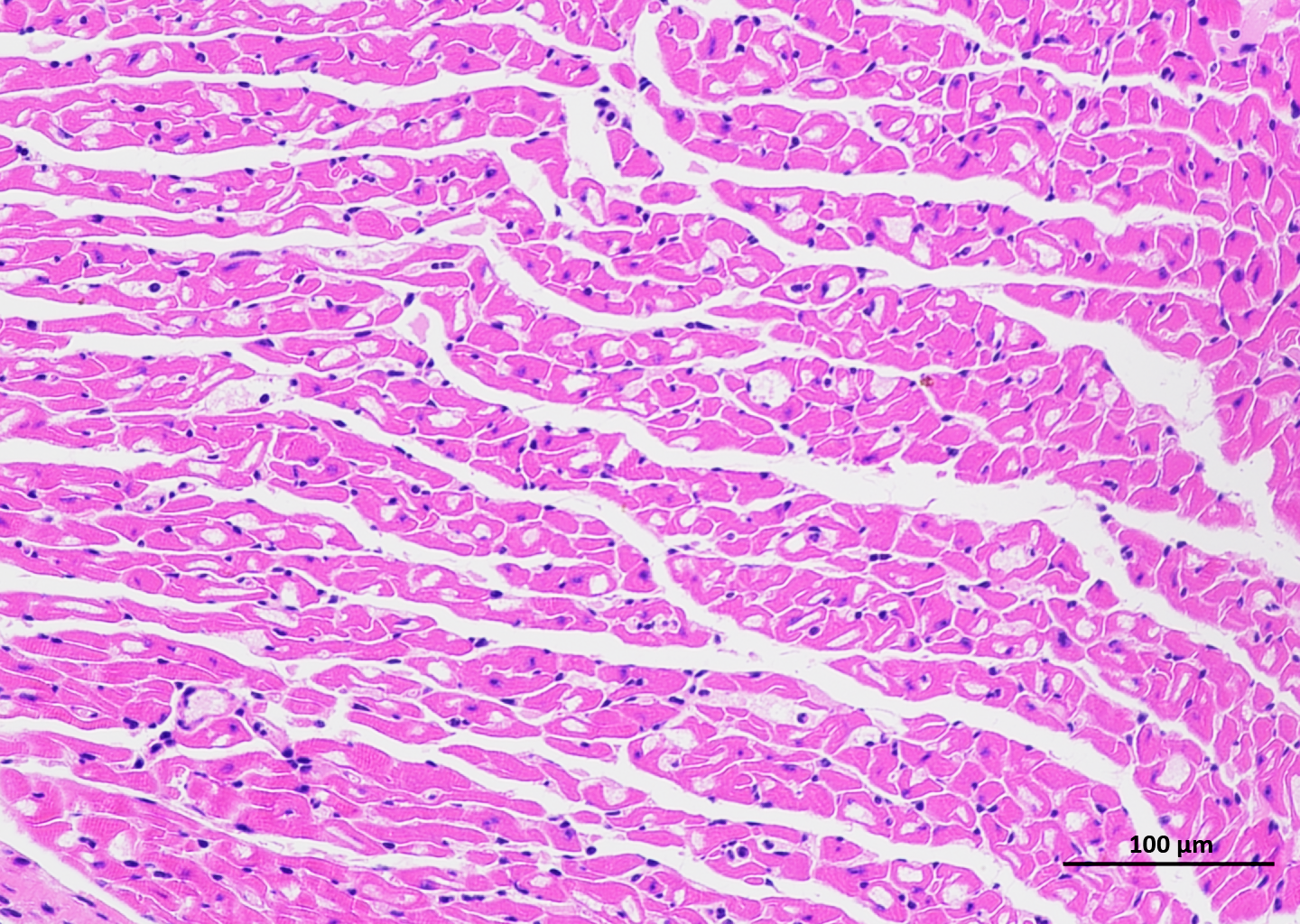

Supplement: Supplementary file 7 [file DataSheet5.zip › HE tissue/ISO.tif]
