# Supplementary material for: Novel idebenone derivatives attenuated oxidative stress injury and myocardial damage
Source: Front Chem. 2025 Feb 24;13:1544616. doi: 10.3389/fchem.2025.1544616 (PMC11891201; doi:10.3389/fchem.2025.1544616)
Supplement: Supplementary file 2 [file Supplementaryfile1.docx]

Novel Idebenone Derivatives Attenuated Oxidative Stress Injury And Myocardial Damage

Yuwei Peng^a,1^, Yishan Guo^a,1^, Xinyi Yang^a,1^, Yulan Liu^a^, Xun Xu^a^, Junhong Chen^a^, Xueyi Liu^a^, Zhiqiang Yu^b^, Dudu Wu^a*^, Zhi Chen^a*^

^a^ School of Pharmacy, Guangdong Medical University, Dongguan, 523808, China

^b^ Institute of Biomedical Health Technology and Engineering, Shenzhen Bay Laboratory, Shenzhen 518132, China

^1^ These authors contributed equally to this work.

* Corresponding authors at: Guangdong Provincial Key Laboratory of Research and Development of Natural Drugs, and School of Pharmacy, Guangdong Medical University, Dongguan 523808, P.R. China.

Email address: [wududuwdd@126.com](mailto:wududuwdd@126.com) (D. Wu)


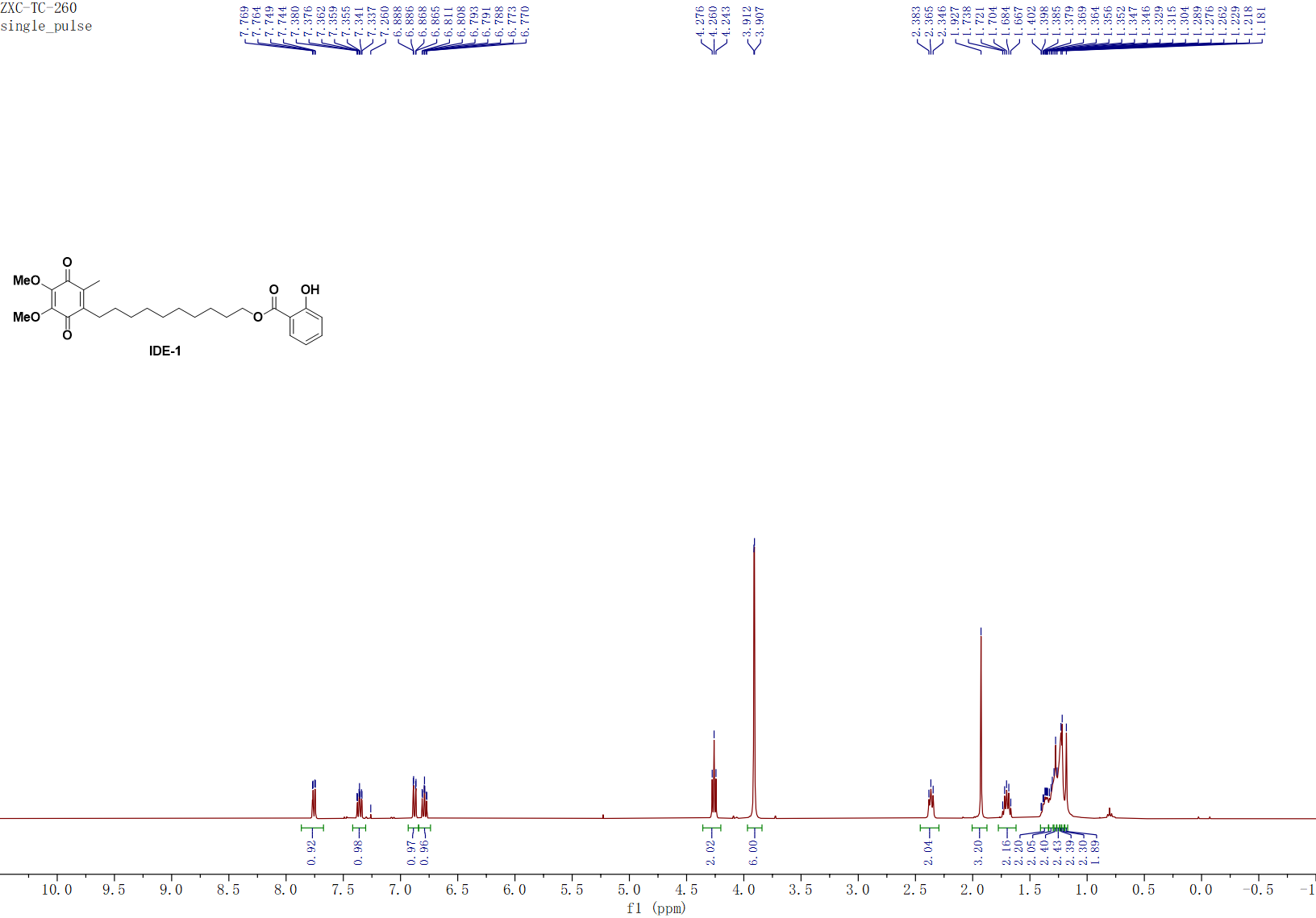


Figure S1. ^1^H NMR of IDE-1 (CDCl_3_, 400 MHz)


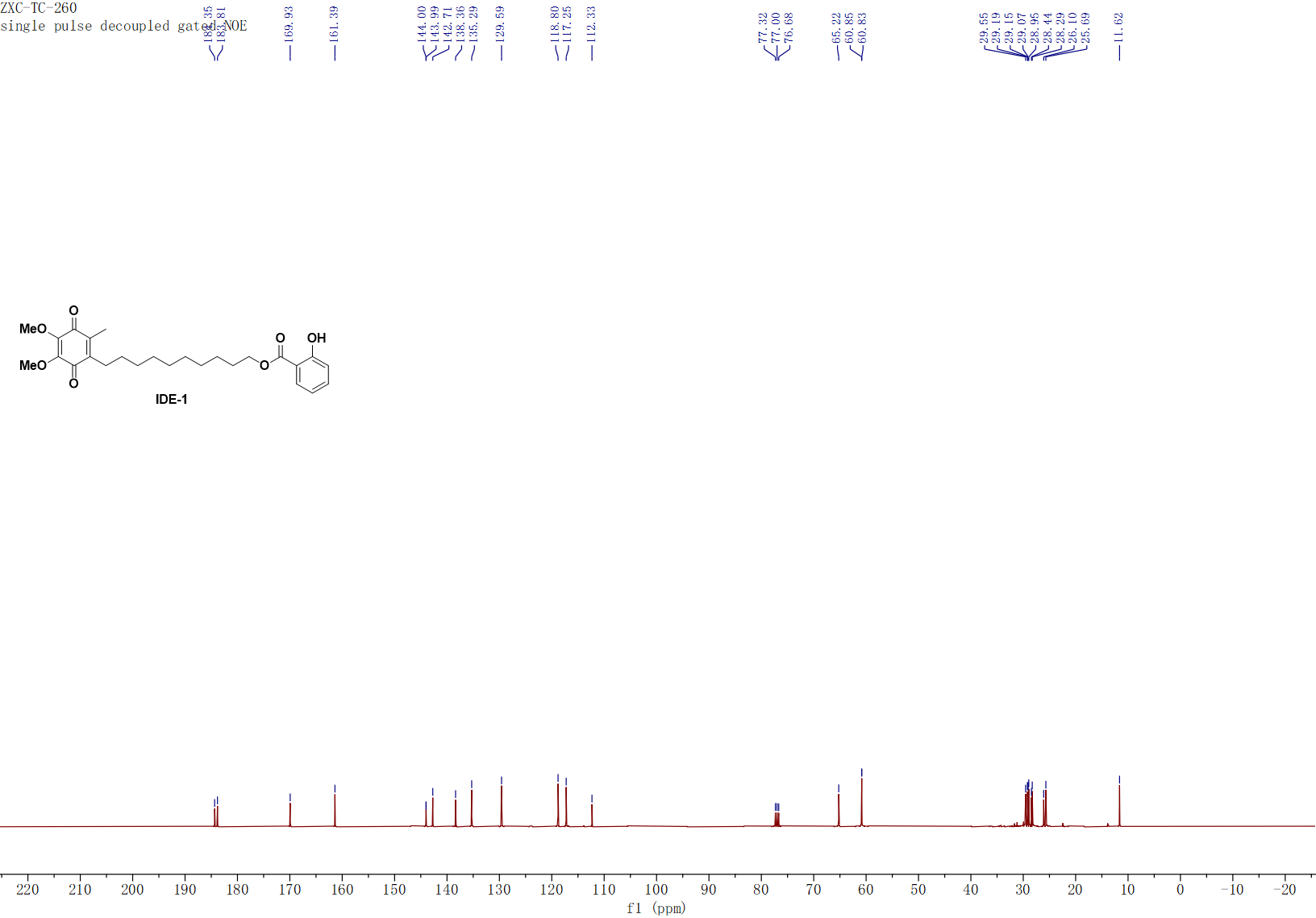
Figure S2. ^13^C NMR of IDE-1 (CDCl_3_, 100 MHz)

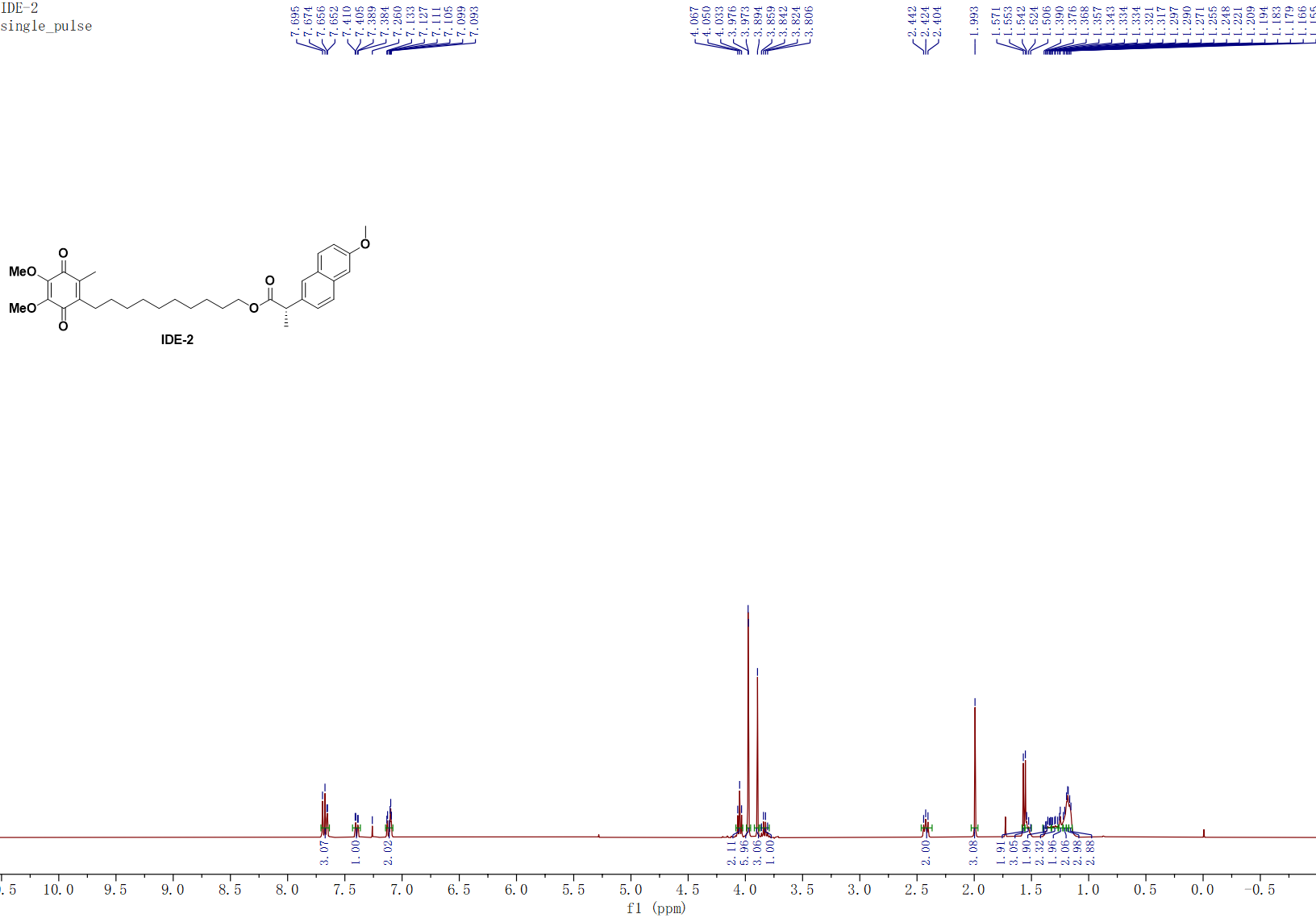


Figure S3. ^1^H NMR of IDE-2 (CDCl_3_, 400 MHz)


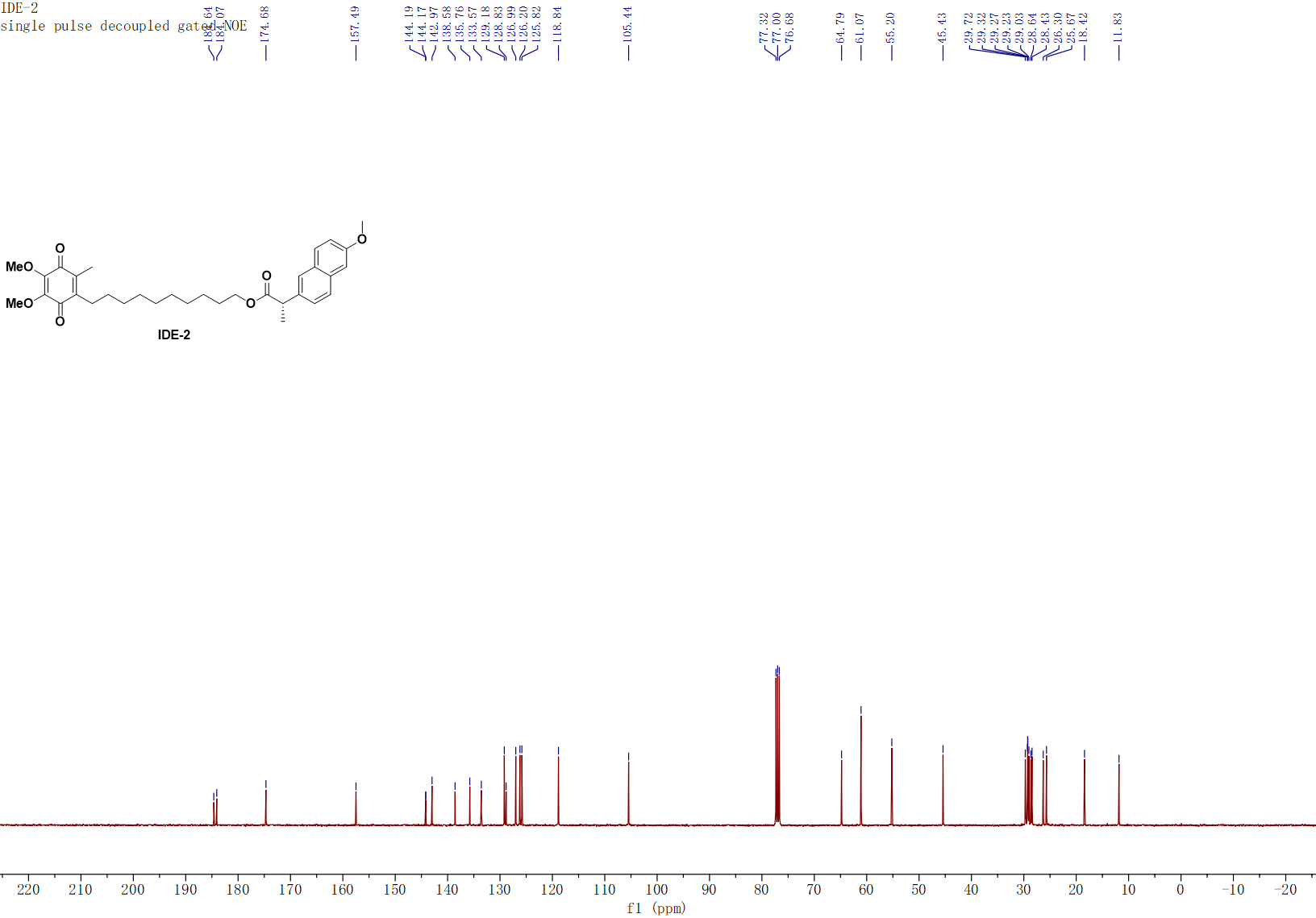


Figure S4. ^13^C NMR of IDE-2 (CDCl_3_, 100 MHz)


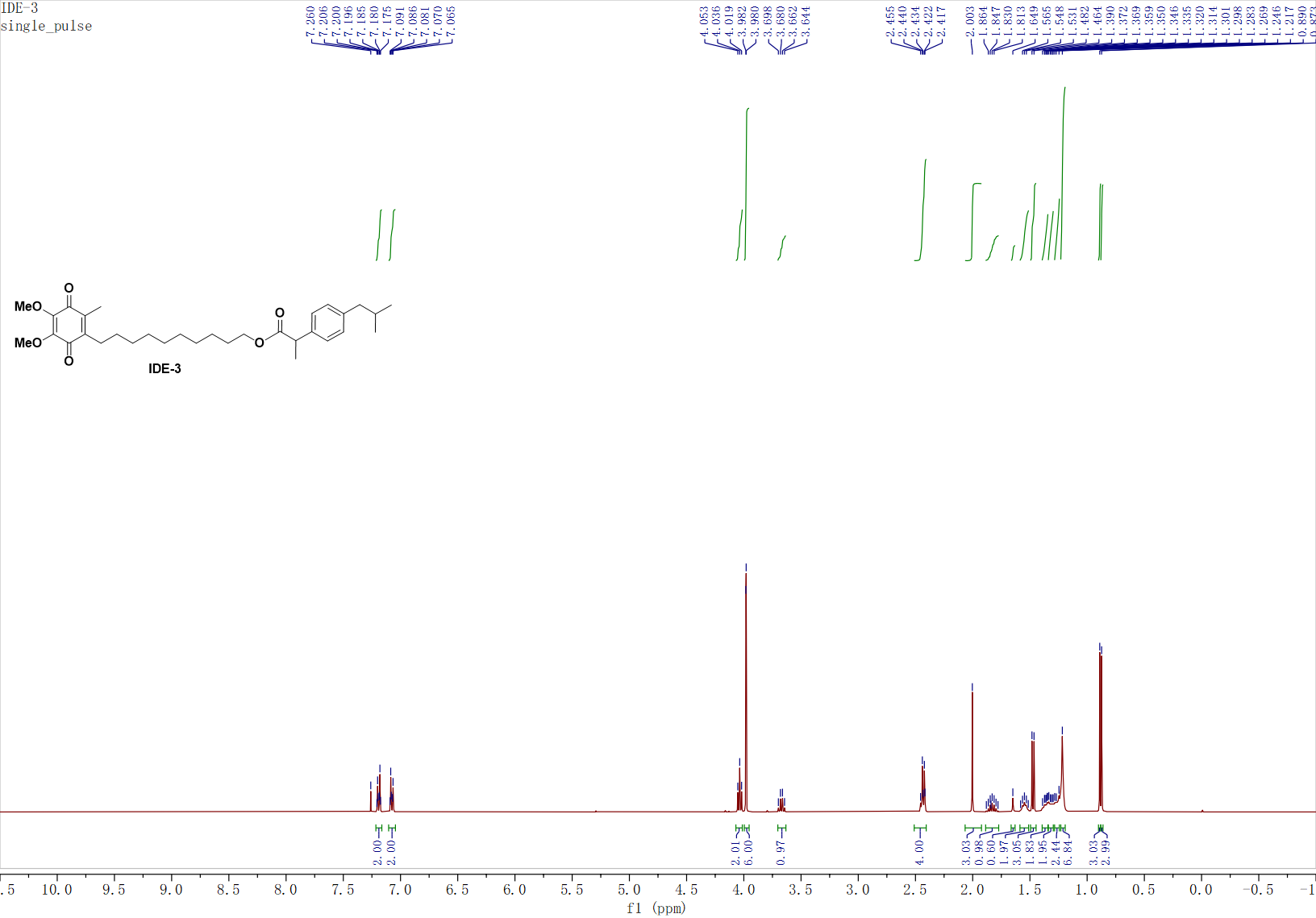


Figure S5. ^1^H NMR of IDE-3 (CDCl_3_, 400 MHz)


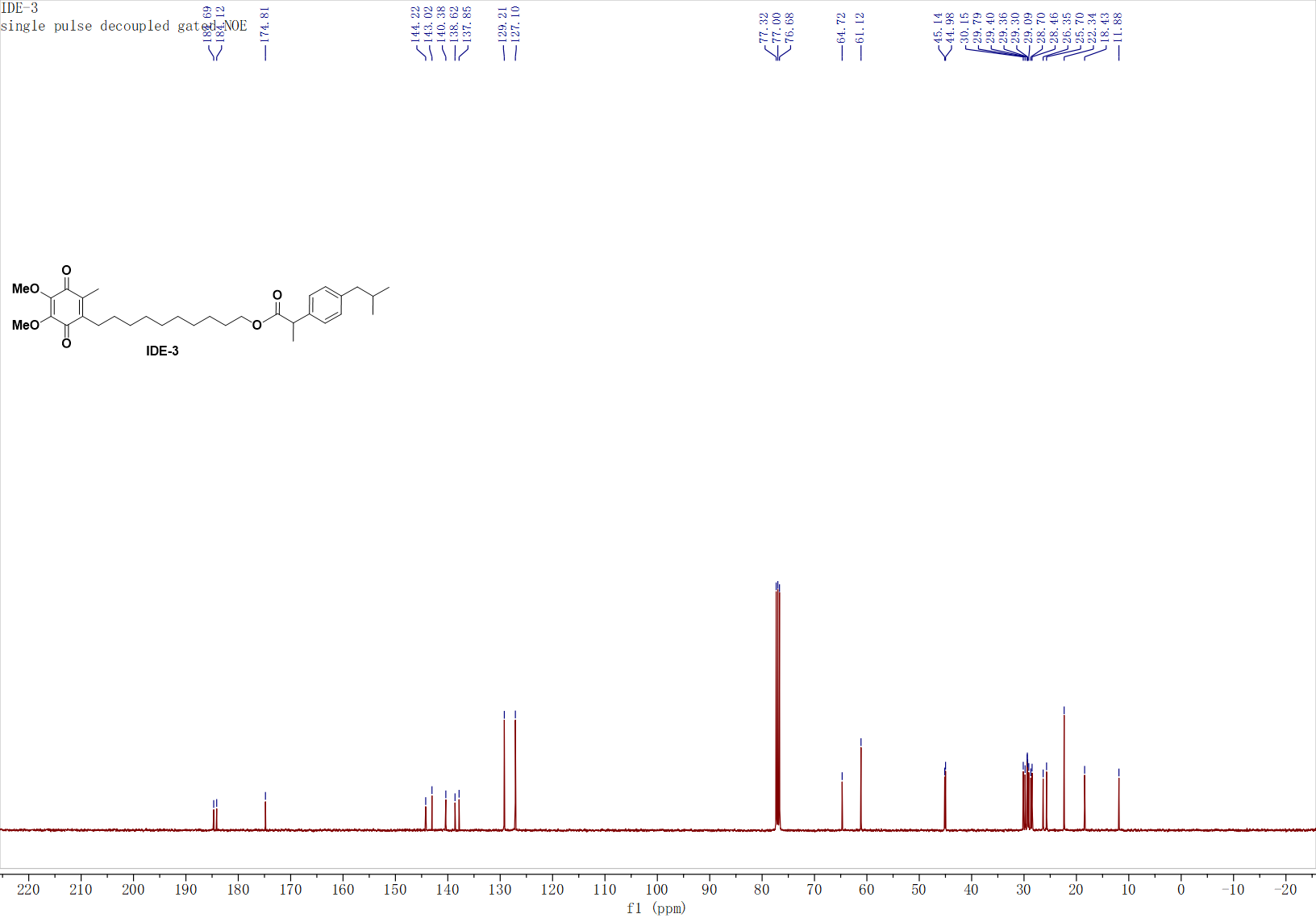
Figure S6. ^13^C NMR of IDE-3 (CDCl_3_, 100 MHz)


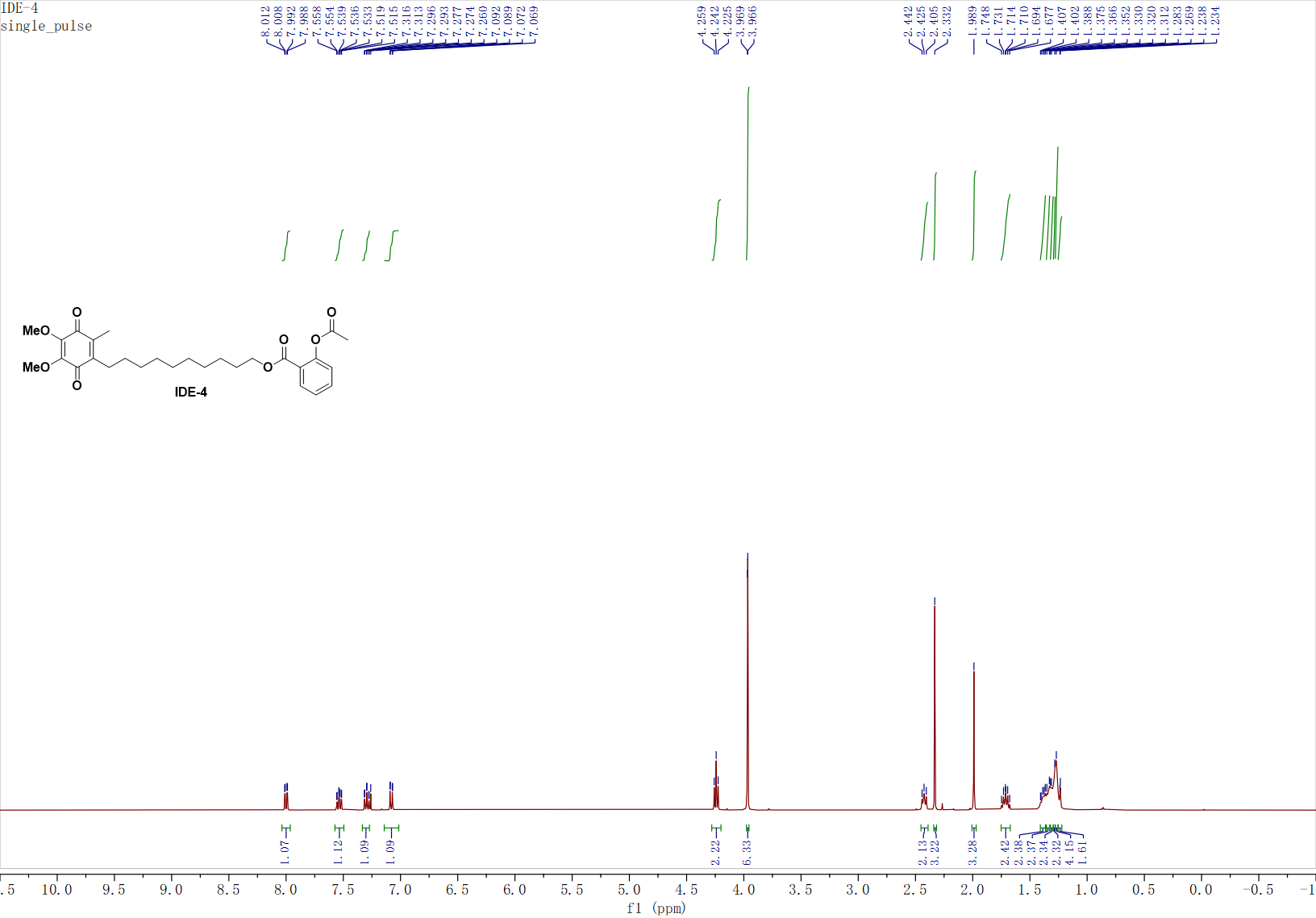


Figure S7. ^1^H NMR of IDE-4 (CDCl_3_, 400 MHz)


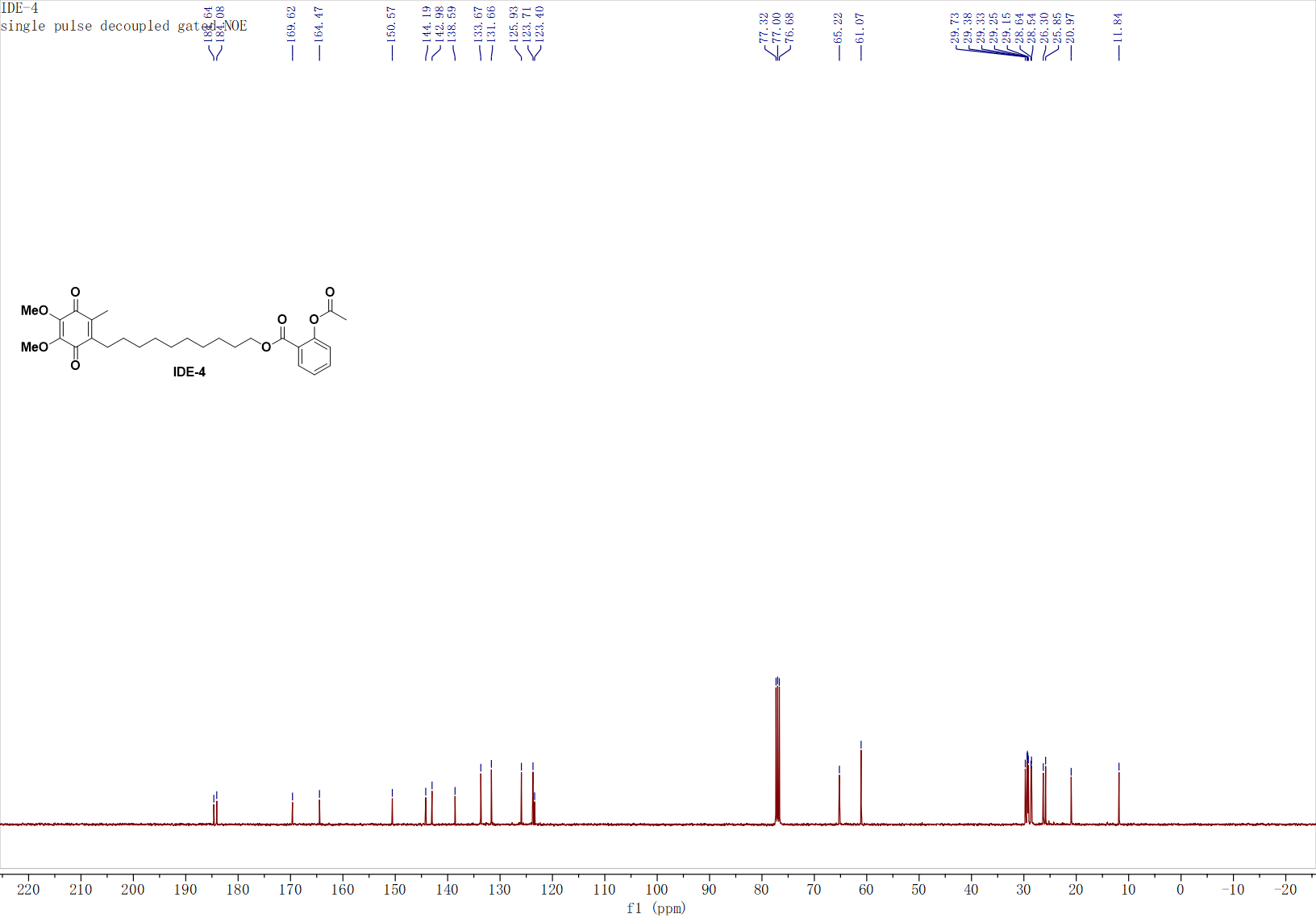
Figure S8. ^13^C NMR of IDE-4 (CDCl_3_, 100 MHz)


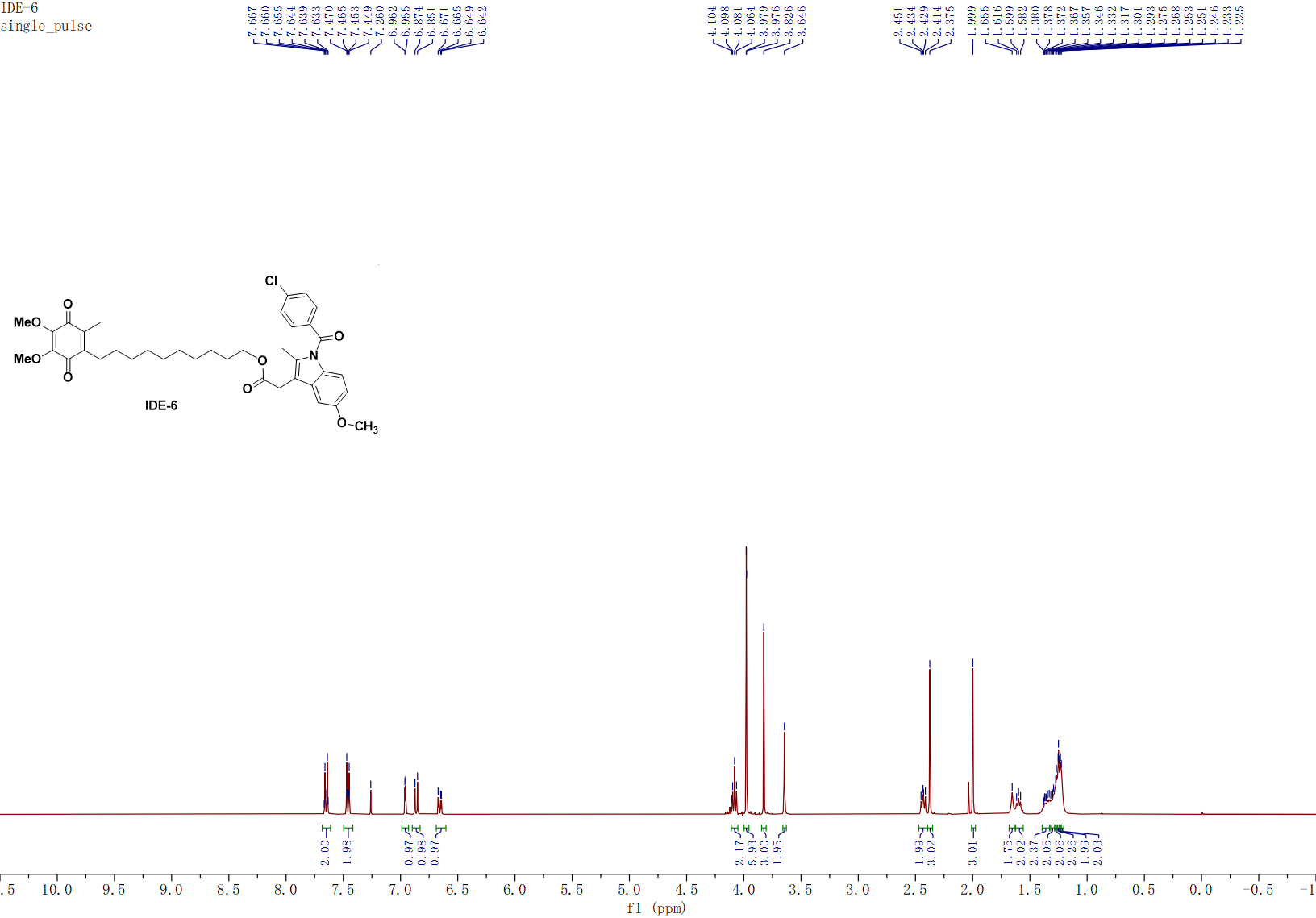


Figure S9. ^1^H NMR of IDE-6 (CDCl_3_, 400 MHz)


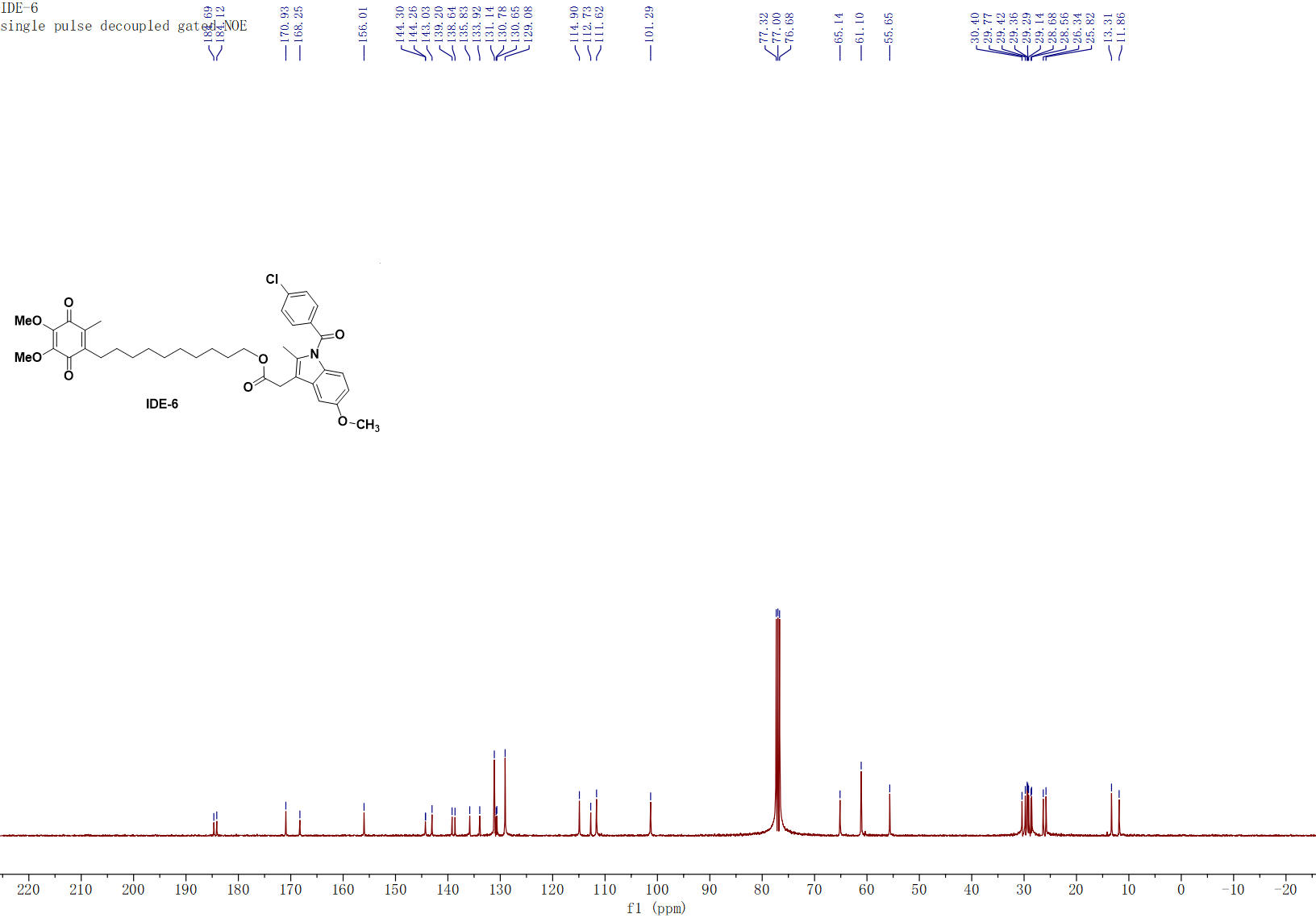
Figure S10. ^13^C NMR of IDE-6 (CDCl_3_, 100 MHz)


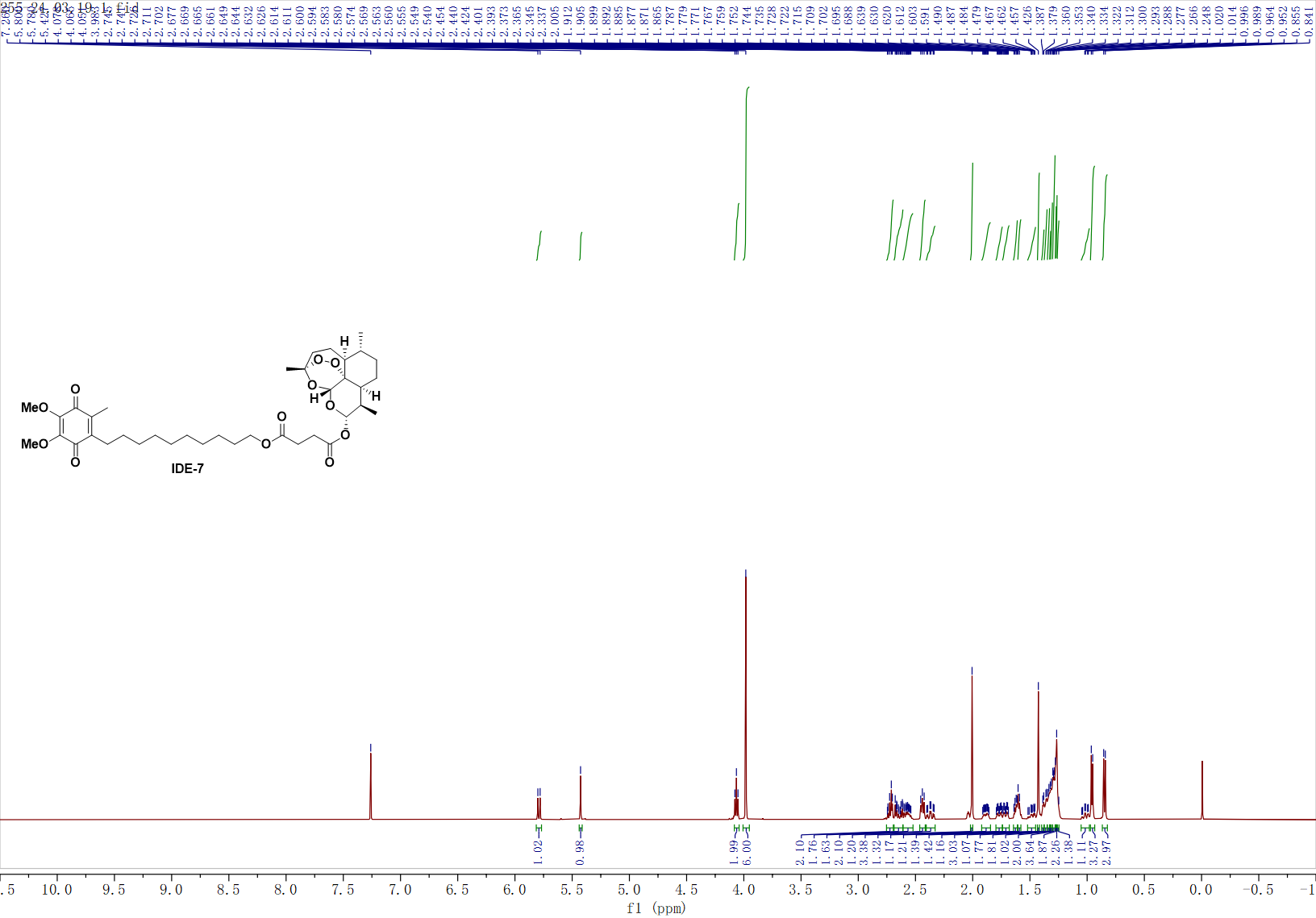
Figure S11. ^1^H NMR of IDE-7 (CDCl_3_, 500 MHz)


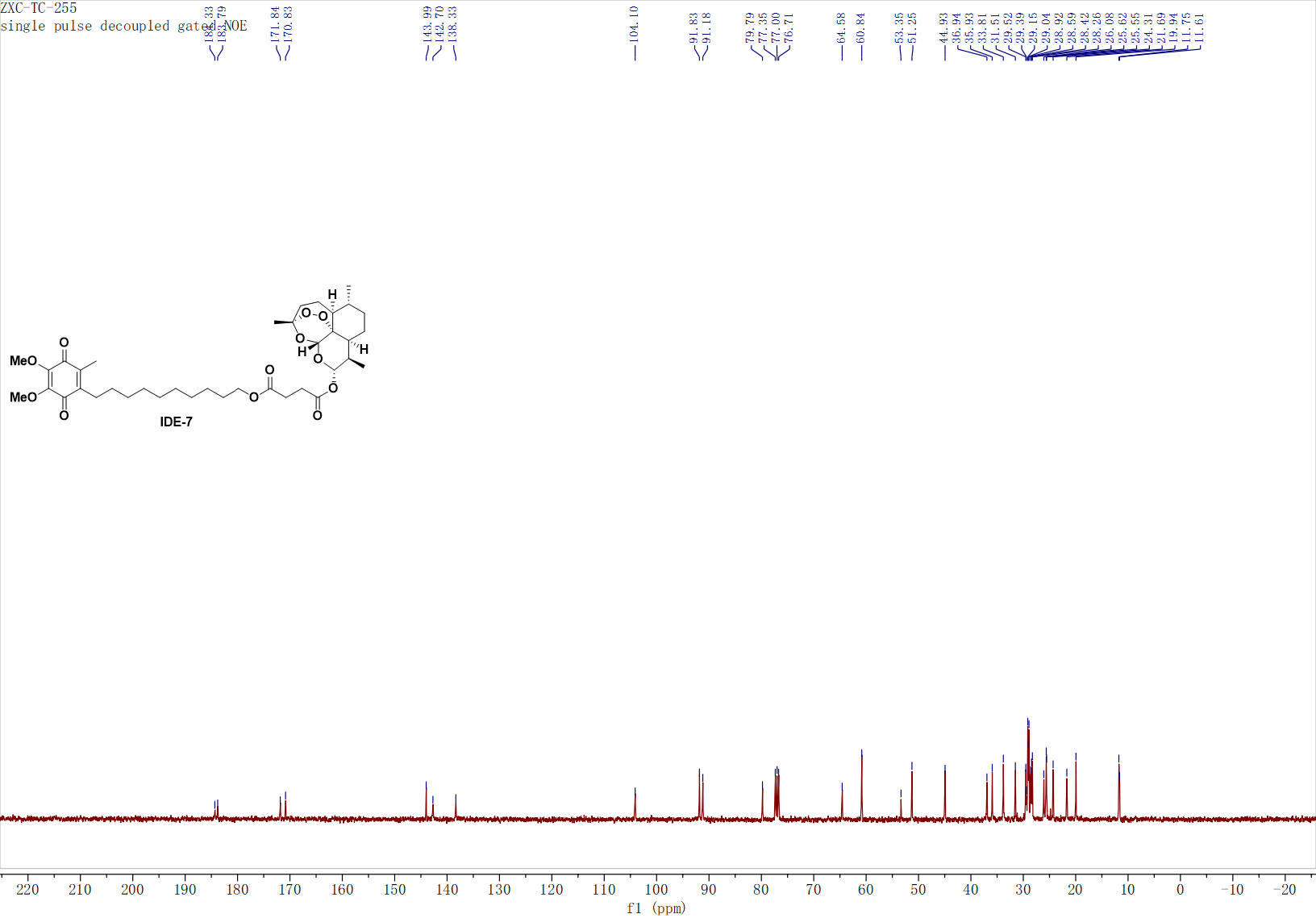
Figure S12. ^13^C NMR of IDE-7 (CDCl_3_, 100 MHz)
